# Supplementary material for: Impact of the COVID-19 pandemic on timeliness and equity of measles, mumps and rubella vaccinations in North East London: a longitudinal study using electronic health records
Source: BMJ Open. 2022 Nov 29;12(12):e066288. doi: 10.1136/bmjopen-2022-066288 (PMC9723415; doi:10.1136/bmjopen-2022-066288)
Supplement: Supplementary data [file bmjopen-2022-066288supp001.pdf]

COVID-19 and childhood MMR immunisation study protocol

Preliminary title: Assessing the impact of the COVID-19 pandemic on timeliness of MMR uptake in east London: cross-sectional analysis of primary care electronic health records

Writing group: Nicola Firman, Milena Marszalek, Ana Gutierrez, Kate Homer, Crystal Williams, Gill Harper, Isabel Dostal, Zaheer Ahmed, John Robson, Carol Dezateux

Background/rationale

The COVID-19 pandemic has disrupted routine health care and services across the UK, through rising COVID infections as well as the introduction of social distancing measures and lockdowns.(1) The United Kingdom (UK) Joint Committee on Vaccination and Immunisation (JCVI) has emphasised the importance of children continuing to receive routine childhood vaccinations according to the national schedule throughout periods of lockdown.(2)

In the UK, childhood vaccine coverage is routinely assessed quarterly using the COVER (Cover of vaccination evaluated rapidly) programme for children who have reached their first, second, or fifth birthdays. Given the timing of data extractions for COVER, the UK Health Security Agency (HAS) has indicated that this system cannot provide reliable information of the impact of the pandemic on vaccine coverage for England. However, evidence suggests that vaccine coverage measured at 24 months among children scheduled to receive Measles, Mumps and Rubella (MMR) vaccination from March 2020 onwards in England was 90.3%, around 0.3% lower than 2019, with average levels in both years well below the WHO coverage target of 95%.(2, 3) This is broadly in line with data from approximately 38% of GP practices in England, which suggests that vaccination counts for MMR vaccine were 2.1% lower by the end of 2020 than for the equivalent period in 2019. Qualitative studies in the UK looking at attitudes towards routine vaccination during the pandemic report parental fear of attending routine vaccination appointments, reduced concern about catching vaccine-preventable diseases during lockdown, and routine appointment re-scheduling. These findings support emerging research that demonstrates disruption across routine child vaccination schedules.(4)

The measures currently used lack information on vaccination timeliness, impacts on existing vaccine coverage inequalities and do not include areas of historic MMR low uptake. This is important as it has been acknowledged that area-level averages conceal geographical clusters of susceptibility which fuel outbreaks.(5) A systematic review found that there is currently no published literature looking at both routine vaccination schedule disruption and consequential impacts on vaccination inequalities during pandemics.(6) More specific literature exists around uptake trends for particular vaccinations - Macdonald et al. demonstrated that MMR uptake during April 2020 dropped by 42·5% in London in comparison to the same time period during 2019.(7) Overall, MMR uptake decreased by 19.8% in England, before recovering over the following months.(8) Most recent local authority coverage data demonstrates that on London, MMR rates have fallen from 83.6% to 82.4% at 24 months from 2019-2021.(9) Areas previously demonstrating lower MMR uptake were worst affected, including London and the Midlands.(7) UK Cover of vaccination evaluated rapidly (COVER) reports look at vaccine coverage at 12, 24 months, and five years, but do not encompass timeliness measures.

These trends reflect pre-existing inequalities observed in MMR uptake in the UK.(10) Historically, the UK lost its measles ‘elimination status’ in 2019 due to consistently low MMR uptake.(11) Further reduction in MMR vaccinations will increase the risk of measles outbreaks, particularly in London where a significant proportion of children start school without the full protection offered by MMR vaccination.(12)

Objectives

This protocol sets out to understand the wider impact of COVID-19 on timeliness of MMR vaccinations in seven east London Clinical Commissioning Groups (CCGs). Specifically:

1. To understand the impact of COVID-19 and periods of lockdown on MMR vaccination timeliness
2. To describe geographical clustering of MMR vaccination timeliness
3. To investigate if COVID-19 has amplified inequalities in receipt of timely MMR vaccination

Methods

Study design

A cross-sectional study using primary care electronic health records (EHRs) from child patients registered at 285 general practices in seven geographically contiguous east London CCGs: Barking & Dagenham, City & Hackney, Havering, Newham, Redbridge, Tower Hamlets, and Waltham Forest.

Study sample

The study population will include all children eligible to receive their first MMR vaccination at age 12 to 18 months old, respectively, in the 19 months before and after 23rd March 2020. All children ever registered at one of the 285 general practices between September 2001 and October 2021 will be included.

Data

Data will be extracted from the North East London (NEL) Discovery Data Service (DDS) which receives primary care electronic health record data on a daily basis from all general practices in NEL, on 23rd November 2021. Events recorded up until 1st November 2021 will be included. All data will be extracted and managed according to UK NHS information governance requirements.

We will analyse person-level data for children born between 22nd August 2017 and 22nd September 2018 (pre-pandemic cohort) and 23rd March 2019 and 1st May 2020 (pandemic cohort). These children would be eligible to receive timely MMR vaccination between 12 and 18 months of age in the 19 months before and after England entered the first lockdown on 23rd March 2020, respectively. For each child, the person-level dataset will contain: a pseudonymised person identifier; calendar week, month and year of birth; ethnic background; sex; 2011 lower super output area (LSOA) of the child's address; CCG; MMR clinical code (Read or SNOMED); and date of MMR vaccination. To avoid disclosure of identifiable data, a proxy date of birth will be derived by assigning the week commencing date using the calendar week and year of birth. Consequently, date of birth could be up to six days earlier than the child's actual date of birth. We will merge 2015 Index of Multiple Deprivation (IMD) score and rank(13) into the datafile using LSOA as the linkage field, and categorise IMD rank into quintiles from most to least deprived.

Following guidance from the Green Book, MMR vaccinations given before a child's first birthday will be ignored.(14) Where available, subsequent MMR records will be treated as the child's first vaccination, otherwise early MMR vaccinations will be retained in the dataset and later considered as "not timely".

The following records will be excluded from analyses:

- Records with an MMR clinical code but no date
- Exact duplicate records
- Duplicate records where the date is the same but MMR clinical code differs
- Latter of multiple MMR events

Statistical methods

We will identify timely MMR vaccination among children aged between 12 and 18 months when they received their MMR vaccination. We will explore variation in the proportion of children receiving timely MMR vaccination by cohort, sex, CCG, ethnic background and IMD quintile, and investigate differences in the proportion of children receiving timely MMR vaccination in each cohort, by these covariates.

To describe geographical clustering of MMR vaccination timeliness we will plot in choropleth maps the proportion of children receiving timely MMR vaccination in each cohort, by LSOA.

We will conduct binary logistic regression to estimate the odds (odds ratio [OR] and 95% CI) of timely MMR vaccination after adjustment for covariates and, to assess whether COVID-19 has widened inequalities in timely vaccination, explore an interaction between cohort and IMD quintile.

Ethics

This study was approved by the Discovery board for service evaluation (measuring what standard of care this service achieved) and analysed routinely acquired de-identified data: hence no research ethics committee approval was required by the Health Research Authority. Access to general practice data is enabled by data sharing agreements between the Discovery Data Service and general practice data controllers. The Discovery Programme Board has approved data access by the REAL Child Health programme.

Strengths and limitations

Strengths of this study include:

- Use of person-level data to comprehensively document the impact of COVID-19 on timely MMR vaccine uptake and coverage
- EHR from seven geographically contiguous CCGs, serving a whole population with a large sample size, including children who have left/deregistered from a practice in NEL, or died
- Access to historic data "pre-pandemic" cohort to use as control group
- Implementation of robust statistical methods

Limitations of this study include:

- Use of calendar week of birth to generate a proxy date of birth
- Primary purpose of EHRs is not for research – so it is possible there will be some data quality issues
    - Large proportions of children with missing ethnic background recorded in their EHR

Funding

This research was funded by a grant from Barts Charity ref: MGU0419. This work was supported by Health Data Research UK (award reference: GPPB1C2), which is funded by the UK Medical Research Council, Engineering and Physical Sciences Research Council, Economic and Social Research Council, Department of Health and Social Care (England), Chief Scientist Office of the Scottish Government Health and Social Care Directorates, Health and Social Care Research and Development Division (Welsh Government), Public Health Agency (Northern Ireland), British Heart Foundation and Wellcome.

References

1. Saxena S, Skirrow H, Bedford H. Routine vaccination during covid-19 pandemic response. Bmj. 2020;369:m2392.
2. Public Health England. Impact of physical distancing measures due to COVID-19 pandemic in England on childhood vaccination counts up to week 41, and vaccine coverage up to August 2020. 2020. Contract No.: 20.
3. Public Health England. Impact of COVID-19 on childhood vaccination counts to week 47, and vaccine coverage to October 2020 in England: interim analyses. Public Health England; 2020 8 December 2020. Contract No.: 23.
4. Bell SL, Clarke R, Paterson P, Mounier-Jack S. Parents' and guardians' views and experiences of accessing routine childhood vaccinations during the coronavirus (COVID-19) pandemic: A mixed-methods study in England 2020.
5. O'Leary ST, Trefren L, Roth H, Moss A, Severson R, Kempe A. Number of Childhood and Adolescent Vaccinations Administered Before and After the COVID-19 Outbreak in Colorado. JAMA pediatrics. 2020.
6. Spencer N, Nathawad R, Arpin E, Johnson S. Pandemics, epidemics and inequities in routine childhood vaccination coverage: a rapid review. BMJ Paediatrics Open. 2020;4(1).
7. McDonald HI, Tessier E, White JM, Woodruff M, Knowles C, Bates C, et al. Early impact of the coronavirus disease (COVID-19) pandemic and physical distancing measures on routine childhood vaccinations in England, January to April 2020. Euro Surveill. 2020;25(19).

8. Skirrow H, Flynn C, Heller A, Heffernan C, Mounier-Jack S, Chantler T. Delivering routine immunisations in London during the COVID-19 pandemic: lessons for future vaccine delivery. A mixed-methods study. BJGP Open. 2021;5(4):BJGPO.2021.0021.
9. Childhood Vaccination Coverage Statistics [Internet]. National Statistics. 2021. Available from: https://app.powerbi.com/view?r=eyJrIjoiZTI3NWZhNzItMTIyZS00OWM2LTg0MzMtOGY5YTJjMGY0MjI1IiwidCI6IjUwZjYwNzFmLWJiZmUtNDAxYS04ODAzLTY3Mzc0OGU2MjllMiIsImMiOjh9.
10. Keenan A, Ghebrehewet S, Vivancos R, Seddon D, MacPherson P, Hungerford D. Measles outbreaks in the UK, is it when and where, rather than if? A database cohort study of childhood population susceptibility in Liverpool, UK. BMJ Open. 2017;7(3):e014106.
11. National Audit Office. Investigation into pre-school vaccinations. 2019. Contract No.: 007836.
12. Tiley KS, White JM, Andrews N, Ramsay M, Edelstein M. Inequalities in childhood vaccination timing and completion in London. Vaccine. 2018;36(45):6726-35.
13. Department for Communities and Local Government. The English Indices of Deprivation 2015 - Frequently Asked Questions (FAQs). 2016.
14. Public Health England. Measles: the green book, chapter 21. In: Public Health England, editor. The Green Book2019.
