# Supplementary material for: Impact of the COVID-19 pandemic on timeliness and equity of measles, mumps and rubella vaccinations in North East London: a longitudinal study using electronic health records
Source: BMJ Open. 2022 Nov 29;12(12):e066288. doi: 10.1136/bmjopen-2022-066288 (PMC9723415; doi:10.1136/bmjopen-2022-066288)
Supplement: Supplementary data [file bmjopen-2022-066288supp002.pdf]

STROBE 2007 (v4) Statement—Checklist of items that should be included in reports of *cross-sectional studies*

Section/Topic	Item #	Recommendation	Reported on page #/in section
**Title and abstract**	1	(*a*) Indicate the study's design with a commonly used term in the title or the abstract	Title
		(*b*) Provide in the abstract an informative and balanced summary of what was done and what was found	Abstract
**Introduction**			
Background/rationale	2	Explain the scientific background and rationale for the investigation being reported	Introduction
Objectives	3	State specific objectives, including any prespecified hypotheses	Introduction
**Methods**			
Study design	4	Present key elements of study design early in the paper	Methods – study design and setting
Setting	5	Describe the setting, locations, and relevant dates, including periods of recruitment, exposure, follow-up, and data collection	Methods – study design and setting
Participants	6	(*a*) Give the eligibility criteria, and the sources and methods of selection of participants	Methods – study population
Variables	7	Clearly define all outcomes, exposures, predictors, potential confounders, and effect modifiers. Give diagnostic criteria, if applicable	Methods – outcome of interest
Data sources/ measurement	8*	For each variable of interest, give sources of data and details of methods of assessment (measurement). Describe comparability of assessment methods if there is more than one group	Methods – covariates of interest
Bias	9	Describe any efforts to address potential sources of bias	Methods – statistical analyses
Study size	10	Explain how the study size was arrived at	Methods – data processing and Supplementary file 3 Figure S2
Quantitative variables	11	Explain how quantitative variables were handled in the analyses. If applicable, describe which groupings were chosen and why	Methods – covariates of interest
Statistical methods	12	(*a*) Describe all statistical methods, including those used to control for confounding	Methods – statistical analyses
		(*b*) Describe any methods used to examine subgroups and interactions	Methods – statistical analyses
		(*c*) Explain how missing data were addressed	Methods – covariates of interest and statistical analyses

		(*d*) If applicable, describe analytical methods taking account of sampling strategy	n/a
		(*e*) Describe any sensitivity analyses	n/a
**Results**			
Participants	13*	(a) Report numbers of individuals at each stage of study—eg numbers potentially eligible, examined for eligibility, confirmed eligible, included in the study, completing follow-up, and analysed	Results and Supplementary file 3 Figure S2
		(b) Give reasons for non-participation at each stage	Supplementary file 3 Figure S2
		(c) Consider use of a flow diagram	Supplementary file 3 Figure S2
Descriptive data	14*	(a) Give characteristics of study participants (eg demographic, clinical, social) and information on exposures and potential confounders	Table 1
		(b) Indicate number of participants with missing data for each variable of interest	Table 1
Outcome data	15*	Report numbers of outcome events or summary measures	Table 2
Main results	16	(*a*) Give unadjusted estimates and, if applicable, confounder-adjusted estimates and their precision (eg, 95% confidence interval). Make clear which confounders were adjusted for and why they were included	Supplementary file 3 Table S5 and Figure 4
		(*b*) Report category boundaries when continuous variables were categorized	n/a
		(*c*) If relevant, consider translating estimates of relative risk into absolute risk for a meaningful time period	n/a
Other analyses	17	Report other analyses done—eg analyses of subgroups and interactions, and sensitivity analyses	Results
**Discussion**			
Key results	18	Summarise key results with reference to study objectives	Discussion – summary of key findings
Limitations	19	Discuss limitations of the study, taking into account sources of potential bias or imprecision. Discuss both direction and magnitude of any potential bias	Discussion – strengths and limitations
Interpretation	20	Give a cautious overall interpretation of results considering objectives, limitations, multiplicity of analyses, results from similar studies, and other relevant evidence	Discussion – implications for research, policy and practice
Generalisability	21	Discuss the generalisability (external validity) of the study results	Discussion – comparison with existing literature
**Other information**			
Funding	22	Give the source of funding and the role of the funders for the present study and, if applicable, for the original study on which the present article is based	Funding

*Give information separately for cases and controls in case-control studies and, if applicable, for exposed and unexposed groups in cohort and cross-sectional studies.

Note: An Explanation and Elaboration article discusses each checklist item and gives methodological background and published examples of transparent reporting. The STROBE checklist is best used in conjunction with this article (freely available on the Web sites of PLoS Medicine at http://www.plosmedicine.org/, Annals of Internal Medicine at http://www.annals.org/, and Epidemiology at http://www.epidem.com/). Information on the STROBE Initiative is available at www.strobe-statement.org.
