# Supplementary material for: Impact of the COVID-19 pandemic on timeliness and equity of measles, mumps and rubella vaccinations in North East London: a longitudinal study using electronic health records
Source: BMJ Open. 2022 Nov 29;12(12):e066288. doi: 10.1136/bmjopen-2022-066288 (PMC9723415; doi:10.1136/bmjopen-2022-066288)
Supplement: Supplementary data [file bmjopen-2022-066288supp003.pdf]

Supplementary tables and figures

Figure S1 – Identifying registrations at the time of first birthday

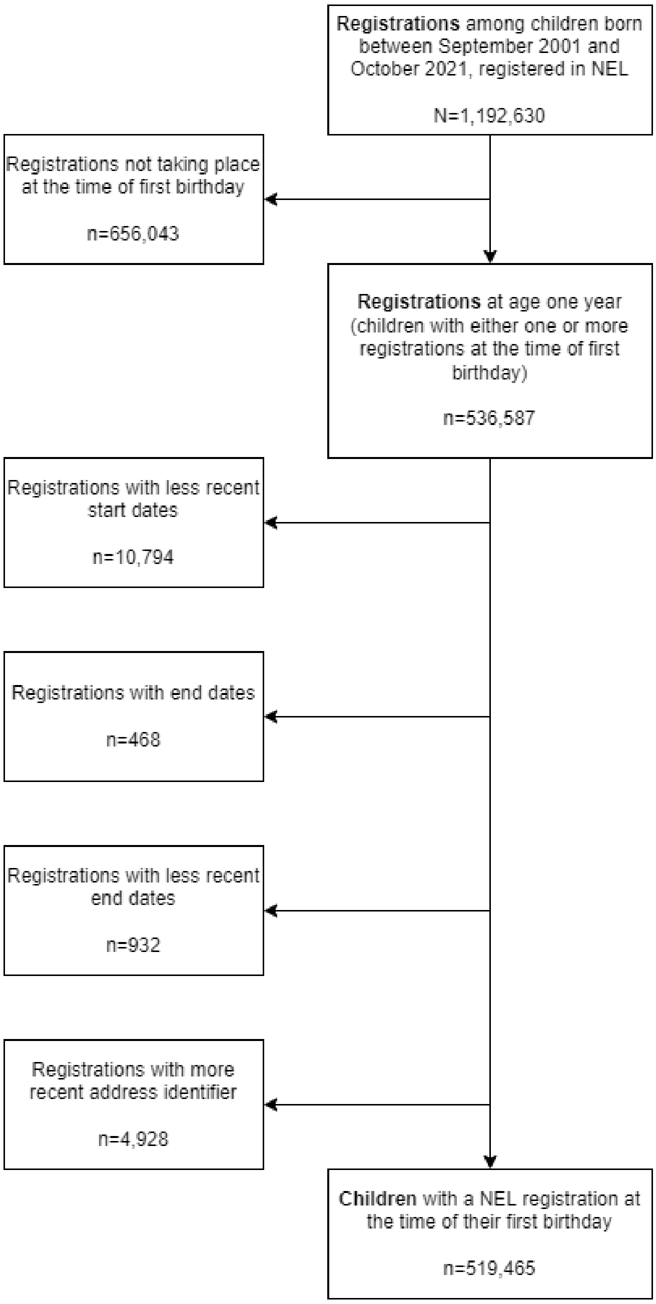

We excluded 656,043 (55.0% of 1,192,630) registrations, which were not current at 12 months of age. For children with multiple concurrent registrations at the time of their first birthday, we applied the following sequence of hierarchical rules to determine which registration should be retained:

Retain

1. *registration with the most recent start date*
2. *registration with a NULL end date (considered more recent/ongoing)*
3. *registration with the most recent end date*
4. *registration with the lowest address identification number (children may have multiple registrations starting and ending on the same date as a result of practice mergers and closures. The lower address identifier is assumed to relate to the original registration)*

Figure S2 – Processing the MMR data file

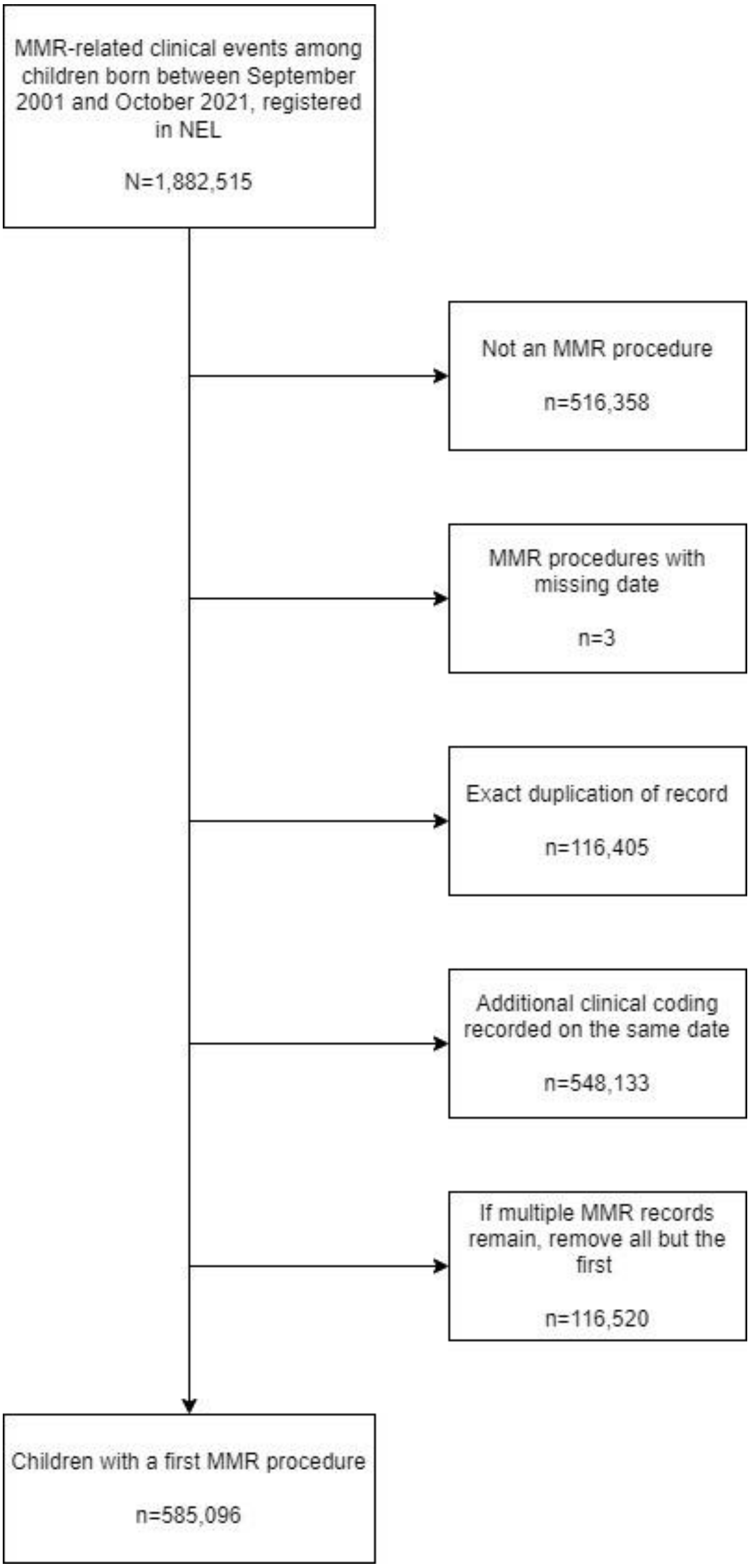

Data relating to MMR vaccinations were extracted separately. The data file was in long format where each row was a unique clinical code, therefore children often have multiple rows indicating various MMR administrative processes and procedures throughout their life, including vaccination invitations, declines, procedures, contraindications, appointment non-attendance, as well as clinical codes relating to the second MMR vaccination. For the purposes of this study, it was necessary to identify the ***first MMR procedure*** *for each child. Of 1,882,515 events, 516,358 recorded events were removed as they were not considered to be a first MMR procedure (see table S1). Records without a date (n=3), as well as duplicate records were removed (n=664,538). Finally, all but the earliest event if a child had more than one MMR vaccination recorded were removed (n=116,520). MMR vaccinations given before the child's first birthday were excluded if the child received another dose after turning 12 months of age, as per guidance from The Green Book.*[1] *Where available, subsequent MMR records were treated as the child's first vaccination, otherwise early MMR vaccinations were retained in the dataset and later considered as "not timely".*

[1] Public Health England. Measles: the green book, chapter 21. In: Public Health England, ed. The Green Book; 2019.

Table S1 - SNOMED clinical codes for first MMR procedures

Events recorded in the primary care electronic heath record using another clinical coding system (e.g. Read v2 or EMIS local codes) have been mapped to relevant SNOMED codes within the Discovery Data Service. This ensures that searching the database using SNOMED codes captured all events regardless of the clinical coding system used.

SNOMED concept ID	Other code	Clinical coding scheme	Code description
38598009	38598009	SNOMED	Measles-mumps-rubella vaccination (procedure)
	65M1.	Read v2	Measles/mumps/rubella vaccn.
	^ESCT1405772	EMIS local	Administration of measles and mumps and rubella vaccine
47435007	47435007	SNOMED	Measles vaccination (procedure)
	65A..	Read v2	Measles vaccination
	65A1.	Read v2	Measles vaccination
	ZV042	Read v2	[V]Measles vaccination
	^ESCT1405845	EMIS local	Administration of measles vaccine
50583002	50583002	SNOMED	Mumps vaccination (procedure)
	65F5.	Read v2	Mumps vaccination
	ZV046	Read v2	[V]Mumps vaccination
	^ESCT1405876	EMIS local	Administration of mumps vaccine
82314000	65B..	Read v2	Rubella vaccination
	ZV043	Read v2	[V]Rubella vaccination
	^ESCT1406118	EMIS local	Administration of rubella vaccine
170364006	65A2.	Read v2	Measles vaccin.+immunoglobulin
432636005	^ESCT1408534	EMIS local	Administration of measles and mumps and rubella and varicella virus vaccine
871909005	^ESCT1397548	EMIS local	Administration of first dose of measles and mumps and rubella and varicella virus vaccine
150971000119104	ZV064	Read v2	[V]Measles-mumps-rubella (MMR) vaccination
308081000000105	65M10	Read v2	First MMR (measles mumps and rubella) vaccination
	Xaeec	Read v3	First MMR (measles mumps and rubella) vaccination
	^ESCTME809974	EMIS local	Measles mumps and rubella vaccination - first dose
505001000000109	9ki1.	Read v2	MMR catch-up vaccination - enhanced services administration
	XaQPr	Read v3	Measles mumps rubella catch-up vaccination
571591000119106	^ESCT1409651	EMIS local	Administration of live attenuated measles mumps and rubella vaccine
1037251000000100	65M11	Read v2	First MMR vaccination given by other healthcare provider
	Xaeeq	Read v3	First MMR vaccination given by other healthcare provider

We included clinical codes relating to administration of mono-components of the first MMR vaccination. After removal of duplicate data entries and merging to the study cohort, 533 children had a clinical code for measles vaccination, and two for mumps vaccination, as opposed to a combined MMR vaccination.

Table S2 – Proportion of children within each cohort who had a MMR procedure recorded in their electronic primary care record

The two datafiles (registrations and MMR procedures) were merged. 531,469 children with an MMR procedure were not matched to the study denominator because they were not eligible for a timely MMR vaccination in the 19 months before or after 23rd March 2020.

	MMR procedure	No MMR procedure	Total
Pre-pandemic (%)	28,173 (84.8)	5,053 (15.2)	33,226 (100)
Pandemic (%)	25,454 (78.5)	6,992 (21.5)	32,446 (100)

Table S3 – SNOMED clinical codes for measles and mumps diagnoses

Code term	SNOMED Concept ID
Measles (disorder)	14189004
Mumps (disorder)	36989005

Table S4 – Ethnic background distribution by Clinical Commissioning Group

	White (%)	Mixed and Other (%)	South Asian (%)	Black (%)	Missing (%)	Total (%)
**Barking & Dagenham**	2,136 (27.6)	567 (7.3)	1,164 (15.1)	772 (10.0)	3,096 (40.0)	7,735 (100)
**City & Hackney**	2,713 (28.9)	1,942 (20.7)	162 (1.7)	576 (6.1)	4,009 (42.6)	9,402 (100)
**Havering**	3,171 (43.2)	500 (6.8)	484 (6.6)	353 (4.8)	2,833 (38.6)	7,341 (100)
**Newham**	2,698 (21.3)	1,656 (13.1)	3,572 (28.2)	1,083 (8.6)	3,659 (28.9)	12,668 (100)
**Redbridge**	2,595 (26.6)	1,032 (10.6)	2,821 (28.9)	404 (4.1)	2,912 (29.8)	9,764 (100)
**Tower Hamlets**	1,223 (13.3)	585 (6.4)	2,419 (26.3)	261 (2.8)	4,715 (51.2)	9,203 (100)
**Waltham Forest**	3,981 (41.7)	1,297 (13.6)	1,061 (11.1)	597 (6.3)	2,623 (27.4)	9,559 (100)
**Total**	18,517 (28.2)	7,579 (11.5)	11,683 (17.8)	4,046 (6.2)	23,847 (36.3)	65,672 (100)

Table S5 – Index of Multiple Deprivation quintile associated with LSOAs with less than 60% of children receiving timely MMR vaccination in the pre-pandemic and pandemic cohorts

	LSOAs with <60% timely vaccination in pre-pandemic cohort (*n*=90)		LSOAs with <60% timely vaccination in pandemic cohort (*n*=153)		All LSOAs (*n*=1,203)	
	**n**	**%**	**n**	**%**	**n**	**%**
**Most deprived**	30	33.3	63	41.2	401	33.3
**2**	45	50.0	61	39.9	445	37.0
**3**	9	10.0	19	12.4	187	15.5
**4**	2	2.2	6	3.9	107	8.9
**Least deprived**	2	2.2	4	2.6	58	4.8
**Missing**	2	2.2	0	0.0	5	0.4
**Total**	90	100	153	100	1203	100

Table S6 – Unadjusted and adjusted odds of timely MMR vaccination

	**Unadjusted**		**Adjusted[1] (*n*=65572[2])**	
	**OR[3]**	**95% CI[4]**	**OR[3]**	**95% CI[4]**
**Cohort**				
Control (ref.)	1		1	
COVID-19	0.80	0.77,0.83	0.79	0.76,0.82
**Sex**				
Male (ref.)	1		1	
Female	1.07	1.03,1.11	1.07	1.03,1.11
**CCG[5]**				
Barking & Dagenham	0.84	0.78,0.89	0.88	0.82,0.94
City & Hackney	0.60	0.56,0.63	0.67	0.63,0.71
Havering	1.78	1.64,1.92	1.53	1.40,1.66
Newham (ref.)	1		1	
Redbridge	0.82	0.77,0.87	0.69	0.64,0.73
Tower Hamlets	1.52	1.42,1.63	1.52	1.42,1.64
Waltham Forest	1.21	1.13,1.29	1.21	1.14,1.30
**Ethnic background**				
White (ref.)	1		1	
Mixed and Other	0.68	0.64,0.73	0.77	0.72,0.82
South Asian	1.33	1.25,1.41	1.39	1.30,1.48
Black	0.64	0.59,0.69	0.70	0.65,0.76
Missing	0.75	0.72,0.79	0.77	0.74,0.81
**IMD[6] quintile**				
Most deprived (ref.)	1		1	
2	1.03	0.99,1.07	1.03	0.99,1.08
3	1.26	1.18,1.33	1.24	1.16,1.32
4	1.87	1.71,2.06	1.78	1.61,1.98
Least deprived	2.42	2.07,2.82	2.09	1.78,2.46

[1] Model mutual adjusting for cohort, sex, clinical commissioning group, ethnic background and Index of Multiple Deprivation quintile. [2] 98 children with missing Index of Multiple Deprivation quintile and two children with "Other" sex were excluded from the adjusted model. [3] Odds ratio. [4] 95% confidence interval. [5] Clinical Commissioning Group. [6] Index of Multiple Deprivation.

Table S7 – Measles and mumps cases recorded in primary care in the pre-pandemic and pandemic periods by Clinical Commissioning Group

	**Pre-pandemic**	**Pandemic**
**Measles**	325	20
**Mumps**	140	34
