# Supplementary material for: Impact of the COVID-19 pandemic on timeliness and equity of measles, mumps and rubella vaccinations in North East London: a longitudinal study using electronic health records
Source: BMJ Open. 2022 Nov 29;12(12):e066288. doi: 10.1136/bmjopen-2022-066288 (PMC9723415; doi:10.1136/bmjopen-2022-066288)
Supplement: Author's manuscript [file bmjopen-2022-066288.draft_revisions.pdf]

BMJ Open is committed to open peer review. As part of this commitment we make the peer review history of every article we publish publicly available.

When an article is published we post the peer reviewers' comments and the authors' responses online. We also post the versions of the paper that were used during peer review. These are the versions that the peer review comments apply to.

The versions of the paper that follow are the versions that were submitted during the peer review process. They are not the versions of record or the final published versions. They should not be cited or distributed as the published version of this manuscript.

BMJ Open is an open access journal and the full, final, typeset and author-corrected version of record of the manuscript is available on our site with no access controls, subscription charges or pay-per-view fees (http://bmjopen.bmj.com).

If you have any questions on BMJ Open's open peer review process please email info.bmjopen@bmj.com

BMJ Open

Impact of the COVID-19 pandemic on timeliness and equity of measles, mumps and rubella vaccinations in north east London: a longitudinal study using electronic health records

Journal:	*BMJ Open*
Manuscript ID	bmjopen-2022-066288
Article Type:	Original research
Date Submitted by the Author:	04-Jul-2022

Complete List of Authors:	Firman, Nicola; Queen Mary University of London, Centre for Primary Care and Public Health<br>Marszalek, Milena; Queen Mary University of London, Centre for Primary Care and Public Health<br>Gutierrez, Ana; Queen Mary University of London, Centre for Primary Care and Public Health<br>Homer, Kate; Queen Mary University of London, Centre for Primary Care and Public Health<br>Williams, Crystal; Queen Mary University of London, Centre for Primary Care and Public Health<br>Harper, Gill; Queen Mary University of London, Centre for Primary Care and Public Health<br>Dostal, Isabel; Queen Mary University of London, Centre for Primary Care and Public Health<br>Ahmed, Zaheer; Queen Mary University of London, Centre for Primary Care and Public Health<br>Robson, John; Queen Mary University of London, Centre for Primary Care and Public Health<br>Dezateux, Carol; Queen Mary University of London, Centre for Primary Care and Public Health

Keywords:	COVID-19, Paediatric infectious disease & immunisation < PAEDIATRICS, PRIMARY CARE, Public health < INFECTIOUS DISEASES, PUBLIC HEALTH

SCHOLARONE™
Manuscripts

I, the Submitting Author has the right to grant and does grant on behalf of all authors of the Work (as defined in the below author licence), an exclusive licence and/or a non-exclusive licence for contributions from authors who are: i) UK Crown employees; ii) where BMJ has agreed a CC-BY licence shall apply, and/or iii) in accordance with the terms applicable for US Federal Government officers or employees acting as part of their official duties; on a worldwide, perpetual, irrevocable, royalty-free basis to BMJ Publishing Group Ltd ("BMJ") its licensees and where the relevant Journal is co-owned by BMJ to the co-owners of the Journal, to publish the Work in this journal and any other BMJ products and to exploit all rights, as set out in our licence.

The Submitting Author accepts and understands that any supply made under these terms is made by BMJ to the Submitting Author unless you are acting as an employee on behalf of your employer or a postgraduate student of an affiliated institution which is paying any applicable article publishing charge ("APC") for Open Access articles. Where the Submitting Author wishes to make the Work available on an Open Access basis (and intends to pay the relevant APC), the terms of reuse of such Open Access shall be governed by a Creative Commons licence – details of these licences and which Creative Commons licence will apply to this Work are set out in our licence referred to above.

Other than as permitted in any relevant BMJ Author's Self Archiving Policies, I confirm this Work has not been accepted for publication elsewhere, is not being considered for publication elsewhere and does not duplicate material already published. I confirm all authors consent to publication of this Work and authorise the granting of this licence.

For peer review only

Impact of the COVID-19 pandemic on timeliness and equity of measles, mumps and rubella vaccinations in north east London: a longitudinal study using electronic health records

Authors

Nicola Firman[1] MSc, Health Data Scientist (0000-0001-5213-5044)*

Milena Marszalek[1], NIHR Academic Clinical Fellow (0000-0001-5825-0609)*

Ana Gutierrez[1], Senior Primary Care Facilitator

Kate Homer[1], Senior Health Data Scientist

Crystal Williams[1] MSc, Senior Data Analyst

Gill Harper[1] PhD, Post-Doctoral Research Fellow in Health Data Science (0000-0002-3492-2076)

Isabel Dostal[1] MSc, Research Assistant

Zaheer Ahmed[1], Informatics Lead

John Robson[1] MD, Clinical Reader in Primary Care Research & Development (0000-0001-6889-0415)

Carol Dezateux[1] MD, Professor of Clinical Epidemiology and Health Data Science (0000-0001-9787-6276)

[1]Centre for Primary Care, Wolfson Institute of Population Health, Barts and the London School of Medicine and Dentistry, Queen Mary University of London, Yvonne Carter Building, 58 Turner Street, London, E1 2AB

*Joint corresponding authors. Nicola Firman (nicola.firman@qmul.ac.uk) and Dr Milena Marszalek (m.marszalek@qmul.ac.uk). Centre for Primary Care, Wolfson Institute of Population Health, Barts and the London School of Medicine and Dentistry, Queen Mary University of London, Yvonne Carter Building, 58 Turner Street, London, E1 2AB

For peer review only

Abstract

Objectives

To quantify the effect of the COVID-19 pandemic on the timeliness of, and geographic and sociodemographic inequalities in, receipt of first Measles Mumps and Rubella (MMR) vaccination.

Design

Longitudinal study using primary care electronic health records.

Setting

285 general practices in north-east London.

Participants

Children born between 23/08/2017 and 22/09/2018 (pre-pandemic cohort) or between 23/03/2019 and 1/05/2020 (pandemic cohort).

Main outcome measure

Receipt of timely MMR vaccination between 12 and 18 months of age.

Methods

We used logistic regression to estimate the odds ratios (OR; 95% confidence intervals [CI]) of receipt of a timely vaccination adjusting for sex, deprivation, ethnic background, and Clinical Commissioning Group. We plotted choropleth maps of the proportion receiving timely vaccinations.

Results

Timely MMR receipt fell by 4.0% (95% CI: 3.4,4.6) from 79.2% (78.8,79.6) to 75.2% (74.7,75.7) in the pre-pandemic (*n*=33,226; 51.3% boys) and pandemic (*n*=32,446; 51.4%) cohorts, respectively. After adjustment, timely vaccination was less likely in the pandemic cohort (0.79; 0.76,0.82), children from Black (0.70; 0.65,0.76), Mixed/Other (0.77; 0.72,0.82) or with missing (0.77; 0.74,0.81) ethnic backgrounds, and more likely in girls (1.07; 1.03,1.11) and those from South Asian backgrounds (1.39; 1.30,1.48). Children living in the least deprived areas were more likely to receive a timely MMR (2.09; 1.78,2.46) but there was no interaction between cohorts and deprivation (Wald statistic: 3.44; *p*-value=0.49). The proportion of neighbourhoods where less than 60% of children received timely vaccination increased from 7.5% to 12.7% during the pandemic.

Conclusions

The COVID-19 pandemic was associated with a significant fall in timely MMR receipt and increased geographic clustering of measles susceptibility in an area of historically low and inequitable MMR coverage. Immediate action is needed to avert measles outbreaks and support primary care to deliver timely and equitable vaccinations.

For peer review only

For peer review only - http://bmjopen.bmj.com/site/about/guidelines.xhtml

Strengths and limitations of this study

- We used routine primary care electronic health records available in near real time for an entire population of children registered with all NHS general practices in one region of London.
- Coding of routine childhood vaccinations by primary care teams in north east London is enabled by data entry templates with standardised coding enabling high quality recording of childhood vaccinations at the point of care.
- We used robust statistical methods to investigate inequalities in MMR timeliness and the impact of the COVID-19 pandemic.
- Ethnic background was not recorded in the primary care electronic health records of more than one third of children in our study sample.

For peer review only

For peer review only - http://bmjopen.bmj.com/site/about/guidelines.xhtml

Introduction

The COVID-19 pandemic disrupted routine healthcare and services across the United Kingdom (UK), through rising COVID infections as well as the introduction of social distancing measures and lockdowns.(1) The UK Joint Committee on Vaccination and Immunisation emphasised the importance of continued receipt of routine vaccinations throughout periods of lockdown.(2)

In the 12 months to March 2021, an average of 90.3% of children scheduled to receive a first Measles, Mumps and Rubella (MMR) vaccination had been vaccinated by 24 months of age in England. This was approximately 0.3% lower than for the same period to March 2020, with average levels in both years well below the World Health Organization (WHO) coverage target of 95%.(3)

These national averages conceal significant geographic inequity. The most recent annual local authority coverage data demonstrates that in London, MMR coverage fell from 83.6% to 82.4% at 24 months from 2019-2021.(3) These findings indicate significant disruption across routine child vaccination schedules which are worse in regions with historically low uptake,(4) reflecting pre-existing inequalities observed in MMR uptake in the UK(5) resulting in a measles outbreak and loss of measles 'elimination status' in 2019.(6) Little is known about the routine vaccination schedule disruption and consequential impacts on vaccination inequalities during pandemics,(7) but further reductions in MMR uptake will increase the risk of further measles outbreaks, particularly in London where a significant proportion of children start school without the full protection offered by MMR vaccination.(8)

Methods currently used to assess vaccine coverage lack information on timeliness, as well as social, ethnic and geographic inequalities. Because of their retrospective nature, these methods are not actionable. This is important as it has been acknowledged that regional averages conceal geographical clusters of susceptibility in smaller areas which fuel outbreaks.(9) We examined the impact of the COVID-19 pandemic on timeliness of the first MMR vaccinations in north east London. Specifically, we aimed to quantify the impact of COVID-19 on timeliness of the first MMR vaccination and investigate whether inequalities in receipt of timely MMR vaccination and its geographic clustering were amplified during the pandemic.

Methods

Study design and setting

We carried out a longitudinal study using primary care electronic health records (EHRs) from 285 general practices (GP) in seven geographically contiguous North East London (NEL) Clinical Commissioning Groups (CCGs): Barking & Dagenham, City & Hackney, Havering, Newham, Redbridge, Tower Hamlets, and Waltham Forest. The study protocol can be found in supplementary file 1 and STrengthening the Reporting of OBservational studies in Epidemiology (STROBE) checklist in supplementary file 2.

Study population

We defined two cohorts of children eligible to receive their first MMR vaccination between 12 and 18 months of age in the 19 months before and after 23rd March 2020 – the date at which the first national lockdown commenced in the UK. The pre-pandemic cohort comprised those born between 23rd August 2017 and 22nd September 2018, and the pandemic cohort those born between 23rd March 2019 and 1st May 2020.

Data Sources

Pseudonymised data were provided from the NEL Discovery Data Service which receives primary care EHR data on a daily basis from all general practices in NEL. Demographic and clinical data were extracted for 1,192,630 children born between September 2001 and October 2021, ever registered with a NEL general practice and

For peer review only - http://bmjopen.bmj.com/site/about/guidelines.xhtml

including children who may have died or left the area. Data were extracted on 23rd November 2021 and included all clinical events up to 1st November 2021. All data were extracted and managed according to UK National Health Service (NHS) information governance requirements.

Data processing

We identified 519,465 children with a NEL GP registration at the time of their first birthday (supplementary file 3 figure S1) and retained only those eligible for the pandemic and pre-pandemic cohorts (figure 1).

We extracted sociodemographic and geographic data for each child, together with - for each child - all clinical events relating to MMR procedures. Documentation of the processing of MMR events can be found in supplementary file 3 - figure S2 and tables S1 and S2. As we did not have access to date of birth, a proxy date of birth was derived by assigning day of birth to first day of the week using the calendar week and year of birth.

Outcome of interest

We defined timely MMR vaccination as receipt of the first MMR vaccination between 12 and 18 months of age, which is consistent with NHS England's definition of a timely MMR vaccination for the Quality and Outcomes Framework (QOF).(10) A vaccination considered not timely may have been given too early (before 12 months of age), late (after 18 months of age) or not recorded (never or not yet received).

Covariates of interest

We merged 2019 Index of Multiple Deprivation (IMD) rank(11) into the datafile using the 2011 Lower Super Output Area (LSOA) - an area with an average population of 1500 people or 650 households - as the linkage field, and categorised IMD rank into the national quintiles from most to least deprived. Ethnic background was categorised using the NHS classification from information recorded in the primary care record. We grouped ethnic background into four mutually exclusive groups: White ('White British', 'White Irish', or 'any other White background'); Black ('Black African', 'Black Caribbean', or 'any other Black background'); South Asian ('Indian', 'Pakistani', 'Bangladeshi' or 'Sri Lankan'); and Mixed/Other ('any other ethnic background', 'mixed ethnicity', 'Chinese' or 'Asian other'). A Missing category was created where ethnicity was not coded in the primary care record.

Statistical analyses

We explored variation in the proportion of children receiving a timely MMR vaccination by cohort, sex, CCG, ethnic background and IMD quintile, and described the differences in the proportion of children receiving timely MMR vaccination in each cohort by these covariates.

For children with a GP recorded address with an associated LSOA in NEL, we plotted choropleth maps of the proportion of children receiving timely MMR vaccination in each cohort and the change in proportion between the two cohorts by LSOA, to visualise geographical clustering of MMR vaccination timeliness. LSOAs with fewer than ten eligible children in either the pre-pandemic or pandemic cohorts were suppressed.

We conducted binary logistic regression to estimate the adjusted odds (odds ratio [OR] and 95% confidence interval [CI]) of timely MMR vaccination after adjustment for covariates. We tested an interaction between cohort and IMD quintile to assess whether COVID-19 had widened inequalities in timely vaccination. All analyses were conducted using Stata (MP/15.0).

Patient and public involvement

For peer review only

For peer review only - http://bmjopen.bmj.com/site/about/guidelines.xhtml

We involved patients and the public in the communication of study results and dissemination within the local community using accepted principles from the UK Standards for Public Involvement.(12) The aim was to raise awareness of the importance of inequalities in timely childhood vaccinations. We established a patient advisory group, comprising six parents, to co-produce dissemination materials.

Results

The pre-pandemic and pandemic cohorts comprised 33,226 (51.3% boys) and 32,446 (51.4% boys) children respectively (figure 1). The cohorts were similar with respect to demographic characteristics: the majority lived in the most deprived areas and were from diverse ethnic backgrounds (table 1 and supplementary file 3 table S3). Timely MMR receipt was 4.0% (95% CI: 3.4,4.6) lower in the pandemic compared with the pre-pandemic cohort.

Table 1 – Sample characteristics

	**Pre-pandemic cohort (*n*=33,226)**			**Pandemic cohort (*n*=32,446)**			**All (*n*=65,672)**		
	n	**%**	**95% CI[1]**	***n***	**%**	**95% CI[1]**	***n***	**%**	**95% CI[1]**
**Sex**									
Male	17055	51.3	50.8,51.9	16665	51.4	50.8,49.2	33720	51.4	51.0,51.7
Female	16169	48.7	48.1,49.2	15781	48.6	48.1,49.2	31950	48.6	48.3,49.0
Other	2			0			2		
**CCG[2]**									
Barking & Dagenham	3916	11.8	11.4,12.1	3819	11.8	11.4,12.1	7735	11.8	11.5,12.0
City & Hackney	4771	14.4	14.0,14.7	4631	14.3	13.9,14.7	9402	14.3	14.1,14.6
Havering	3684	11.1	10.8,11.4	3657	11.3	10.9,11.6	7341	11.2	10.9,11.4
Newham	6458	19.4	19.0,19.9	6210	19.1	18.7,19.6	12668	19.3	19.0,19.6
Redbridge	4971	15.0	14.6,15.3	4793	14.8	14.4,15.2	9764	14.9	14.6,15.1
Tower Hamlets	4605	13.9	13.5,14.2	4598	14.2	13.8,14.6	9203	14.0	13.8,14.3
Waltham Forest	4821	14.5	14.1,14.9	4738	14.6	14.2,15.0	9559	14.6	14.3,14.8
**Ethnic background**									
White	9579	28.8	28.3,29.3	8938	27.6	27.1,28.0	18517	28.2	27.9,28.5
Mixed and Other	3813	11.5	11.1,11.8	3766	11.6	11.3,12.0	7579	11.5	11.3,11.8
South Asian	5881	17.7	17.3,18.1	5802	17.9	17.5,18.3	11683	17.8	17.5,18.1
Black	2054	6.2	5.9,6.4	1992	6.1	5.9,6.4	4046	6.2	6.0,6.3
Missing	11899	35.8	35.2,36.3	11948	36.8	36.3,37.4	23847	36.3	35.9,36.7
**IMD[3] quintile**									
Most deprived	12436	37.4	36.9,38.0	11995	37.0	36.4,37.5	24431	37.2	36.8,37.6
2	13464	40.5	40.0,41.1	13306	41.0	40.5,41.5	26770	40.8	40.4,41.1
3	4533	13.6	13.3,14.0	4400	13.6	13.2,13.9	8933	13.6	13.3,13.9
4	1883	5.7	5.4,5.9	1956	6.0	5.8,6.3	3839	5.9	5.7,6.0
Least deprived	847	2.6	2.4,2.7	754	2.3	2.2,2.5	1601	2.4	2.3,2.6
Missing	63	0.2	0.1,0.2	35	0.1	0.1,0.2	98	0.2	0.1,0.2
**Timely MMR vaccination[4]**									
Yes	26315	79.2	78.8,79.6	24402	75.2	74.7,75.7	50717	77.2	76.9,77.5
No	6911	20.8	20.4,21.2	8044	24.8	24.3,25.3	14955	22.8	22.5,23.1
Early	*180*	*0.5*	*0.5,0.6*	*120*	*0.4*	*0.3,0.4*	*300*	*0.5*	*0.4,0.5*
Late	*1678*	*5.1*	*4.8,5.3*	*932*	*2.9*	*2.7,3.1*	*2610*	*4.0*	*3.8,4.1*

For peer review only

For peer review only - http://bmjopen.bmj.com/site/about/guidelines.xhtml

Not yet received	*5053*	*15.2*	*14.8,15.6*	*6992*	*21.5*	*21.1,22.0*	*12045*	*18.3*	*18.0,18.6*

[1] 95% confidence interval. [2] Clinical Commissioning Group. [3] Index of Multiple Deprivation. [4] Receipt of first MMR vaccination between 12 and 18 months of age. Not timely is further broken down into three groups: early (before age 12 months), late (after age 18 months) and not yet received.

Children from White, Mixed/Other, and Black ethnic backgrounds had the lowest - and children from South Asian ethnic backgrounds the highest - percentage timely MMR receipt (table 2). There was a strong positive gradient in vaccination timeliness by IMD quintile: relative to those living in the least deprived quintile, the proportion of children receiving a timely MMR vaccination was 10.8% (8.6,13.0) and 14.3% lower (11.8,16.8) in the pre-pandemic and pandemic cohorts respectively.

Table 2 – Proportion of children with timely MMR vaccination[1] in each cohort, and the percentage point difference between pre-pandemic and pandemic cohorts, by sociodemographic characteristics

	**Pre-pandemic cohort (*n*=26,315)**			**Pandemic cohort (*n*=24,402)**			**Percentage point difference[2]**	
	n	**%**	**95% CI[3]**	***n***	**%**	**95% CI[3]**	**%**	**95% CI[3]**
**Sex**								
Male	13362	78.3	77.7,79.0	12480	74.9	74.2,75.5	-3.4	-4.3,-2.5
Female	12953	80.1	79.5,80.7	11922	75.5	74.9,76.2	-4.6	-5.5,-3.7
**CCG[4]**								
Barking & Dagenham	2999	76.6	75.2,77.9	2725	71.3	69.9,72.8	-5.3	-7.3,-3.3
City & Hackney	3403	71.3	70.0,72.6	2897	62.6	61.2,63.9	-8.7	-10.6,-6.8
Havering	3205	87.0	85.9,88.0	3095	84.6	83.4,85.8	-2.4	-4.0,-0.8
Newham	5077	78.6	77.6,79.6	4717	76.0	74.9,77.0	-2.6	-4.1,-1.1
Redbridge	3713	74.7	73.5,75.9	3483	72.7	71.4,73.9	-2.0	-3.7,-0.3
Tower Hamlets	3977	86.4	85.3,87.3	3737	81.3	80.1,82.4	-5.1	-6.6,-3.6
Waltham Forest	3941	81.7	80.6,82.8	3748	79.1	77.9,80.2	-2.6	-4.2,-1.0
**Ethnic background**								
White	7865	82.1	81.3,82.9	6874	76.9	76.0,77.8	-5.2	-6.4,-4.0
Mixed and Other	2872	75.3	73.9,76.7	2641	70.1	68.6,71.6	-5.2	-7.2,-3.2
South Asian	4966	84.4	83.5,85.3	4824	83.1	82.2,84.1	-1.3	-2.6,0.0
Black	1517	73.9	71.9,75.7	1374	69.0	66.9,71.0	-4.9	-7.7,-2.1
Missing	9095	76.4	75.7,77.2	8689	72.7	71.9,73.5	-3.7	-4.8,-2.6
**IMD[5] quintile**								
Most deprived	9710	78.1	77.3,78.8	8779	73.2	72.4,74.0	-4.9	-6.0,-3.8
2	10523	78.2	77.5,78.8	9865	74.1	73.4,74.9	-4.1	-5.1,-3.1
3	3675	81.1	79.9,82.2	3437	78.1	76.9,79.3	-3.0	-4.7,-1.3
4	1628	86.5	84.8,87.9	1649	84.3	82.6,85.9	-2.2	-4.4,0.0
Least deprived	753	88.9	86.6,90.8	660	87.5	85.0,89.7	-1.4	-4.6,1.8
Missing	26	41.3	29.9,53.7	12	32.3	20.6,51.2	-9.0	-28.7,10.7

[1] Receipt of MMR vaccination between 12 and 18 months of age. [2] Proportion receiving timely MMR vaccination in pandemic cohort minus the proportion receiving timely MMR vaccination in the pre-pandemic cohort. [3] 95% confidence interval. [4] Clinical Commissioning Group. [5] Index of Multiple Deprivation.

The proportion of LSOAs where fewer than 60% of children received a timely MMR vaccination increased from 7.5% (90) to 12.7% (153) in the pandemic cohort (figures 2a and 2b). These were clustered in parts of City & Hackney, Newham, Redbridge, and Barking and Dagenham. Almost half of LSOAs where fewer than 60% of

For peer review only - http://bmjopen.bmj.com/site/about/guidelines.xhtml

children received timely MMR vaccinations were assigned to the most deprived IMD quintile compared to one third in the pre-pandemic cohort (supplementary file 3 table S4). The proportion of children receiving a timely MMR vaccination fell during the pandemic period in 634 (52.7%) out of 1203 LSOAs (figure 3), and these were predominantly located in Tower Hamlets and City & Hackney. The proportion increased in 367 LSOAs (30.5%) and remained the same in 13 (1.1%).

After adjustment, timely MMR receipt was less likely in the pandemic cohort (0.79; 0.76,0.82), children from Black (0.70; 0.65,0.76), Mixed/Other (0.77; 0.72,0.82) or with Missing (0.77; 0.74,0.81) ethnic backgrounds, and more likely in girls (1.07; 1.03,1.11) and those from South Asian backgrounds (1.39; 1.30,1.48). Children living in the least deprived areas were more likely to receive a timely MMR (2.09; 1.78,2.46; Wald test statistic: 201.66; *p*-value<0.0001; figure 4 and supplementary file 3 table S5), but there was no interaction between cohorts and deprivation (Wald statistic: 3.44; *p*-value=0.49). Relative to children registered with a general practice in Newham, timely MMR receipt was less likely among children in Barking & Dagenham (0.88; 0.82,0.94), City & Hackney (0.67; 0.63,0.71) or Redbridge (0.69; 0.64,0.73) and more likely among those registered to a general practice in Havering (1.53; 1.40,1.66), Tower Hamlets (1.52; 1.42,1.64) and Waltham Forest (1.21; 1.14,1.30).

Patient and public involvement

The PPI group reflected on vaccination inequalities, the study design and how results were delivered. Participants expressed reservations about the categorisation of ethnic background and whether more granular categories could be used in future research. They discussed communication and visualisation of results. Dissemination of results is ongoing and informed by advice about accessing hard-to-reach and existing community groups.

Discussion

Summary of key findings

In the period preceding the COVID-19 pandemic in NEL, only 79% of children received their first MMR vaccination on time; this proportion fell by an average of 4% during the pandemic. The gap between the most and least deprived areas increased by 3.5% during the pandemic period. While this relative inequality did not appear to worsen during the pandemic, these average figures conceal marked inequity at a lower geographical level: the percentage of LSOAs, where fewer than 60% of children received their MMR on time increased from 7.5% to 12.7% in the pandemic, particularly in the most deprived LSOAs. Hence delayed receipt of MMR is geographically clustered in more deprived neighbourhoods and this has worsened during the pandemic. These findings are in an area of London where no CCG has met the WHO MMR target of 95% coverage.(3) In the absence of national data, our analyses show for the first time how far this region of London is from achieving the new Quality and Outcomes Framework target for MMR timeliness of 90-95%.(10)

Strengths and limitations

We used routine primary care EHRs available in near real time for an entire population of children registered with all NHS general practices in one region of London. We have been able to demonstrate – in a geographically contiguous area – neighbourhoods with very high proportions of children who are not immunised promptly. These results further highlight the inequalities in vaccination timeliness and infection risks experienced by poorer children, their families and communities.

Coding of routine childhood vaccinations by primary care teams in NEL is enabled by data entry templates with standardised coding enabling high quality recording of childhood vaccinations at the point of care. We used robust statistical methods to investigate inequalities in MMR timeliness and the impact of the COVID pandemic.

For peer review only - http://bmjopen.bmj.com/site/about/guidelines.xhtml

Limitations include missing ethnic codes in 36% of the cohort. Our analyses suggest that those with missing ethnicity were less likely to receive a timely MMR vaccine during the pandemic and highlight the importance of improving routine recording of ethnicity in primary care.

Comparison with existing literature

Our findings align with trends indicating a global decrease in uptake of MMR vaccination, both in developing and developed countries.(13-15) Some studies reported a decline in uptake of more than 50% during the height of the first wave of the pandemic.(16-18) Globally, measles-containing vaccine coverage estimates were 7.9% lower than expected had there been no COVID-19 pandemic, affecting an estimated 8.9 million children.(19) Our findings may be explained in part by evidence from qualitative research studies demonstrating that a transition to remote consultations during the pandemic caused some confusion for parents around attending services for routine vaccination.(4) Internationally, fear of COVID-19 exposure in healthcare settings was also found to play a large role in decreased vaccination uptake,(19-21) despite evidence that the risk to benefit ratio was in favour of continuing vaccination delivery during the pandemic.(22)

The link between childhood vaccination inequalities and ethnicity has been explored in other studies, demonstrating evidence of reduced timeliness in certain ethnic groups.(23) However, there is heterogeneity within these results according to geographical area of interest.(24) We found that children from Black and Mixed/Other ethnic backgrounds were less likely to receive timely vaccination, broadly consistent with findings from studies of COVID-19 vaccination uptake which have shown lowest uptake among people from Black ethnic backgrounds.(25, 26)

Relationships between socioeconomic deprivation and vaccination timeliness appear to be more consistent. Studies in Scotland examining vaccination inequity and timeliness demonstrate that the most deprived decile experienced a nearly 50% increased risk of delayed vaccination relative to the least deprived decile for both doses of MMR.(27)

Our findings are consistent with existing evidence based on Cover of Vaccination Evaluated Rapidly (COVER) programme data which confirm that London has a longstanding and disproportionately lower MMR uptake relative to the rest of the UK.(3, 28) There is recognised variation between different CCGs in NEL, with lowest uptake in City & Hackney. While particular cultural beliefs held by the Charedi Jewish population in City & Hackney are known to influence uptake of the MMR vaccination,(29) recent evidence suggests that difficulties in accessing vaccination services are also an important factor in this community.(30) The factors responsible for differences between the other CCGs merit further investigation.

Mapping measles vaccinations and outbreaks geospatially enables more granular identification of neighbourhoods requiring focussed interventions.(31) Our choropleth maps demonstrate clustering of delayed MMR vaccination in more deprived neighbourhoods – these findings align with previous studies mapping measles outbreak susceptibility(5) and underscore the importance of actionable real-time information on vaccine timeliness to avert further outbreaks of measles.

Implications for research, policy and practice

Gaps in MMR vaccination coverage increase measles-susceptibility, and in 2019, there were 800,000 confirmed cases of measles globally.(32) Measles outbreaks have occurred in 2021 in at least half of the 26 countries that suspended their measles vaccination programs.(22, 32, 33) There is evidence that the introduction of social distancing measures, school closures and travel restrictions reduced exposure to vaccine-preventable childhood infections.(34) In an analysis of English hospital admissions there was a 90% and a 53% reduction in hospital admissions for measles and mumps, respectively, among children aged 0-14 years in the pandemic period compared to the preceding three year average, although this study was unable to examine

For peer review only - http://bmjopen.bmj.com/site/about/guidelines.xhtml

infections managed in primary care.(34) In NEL, we identified 20 measles and 34 mumps cases in primary care during the pandemic period, compared to 326 and 140, respectively, in the equivalent pre-pandemic period (supplementary file 3 table S6).

With a reduction in infection and exposure to infection, measles vaccination may receive less priority in a health care system already facing multiple challenges.(35) Awareness and retention of existing WHO targets is critical to prevent measles outbreaks, especially given that measles is the most infectious virus, with a reproduction number of 12–18.(36) The need for targeted public health interventions around routine childhood vaccinations in the context of the pandemic has been recognised internationally.(37, 38) There is strong evidence to support the effectiveness of primary care led quality improvement programmes to improve vaccine uptake.(28) National measures to tackle these inequalities include NHS England's Quality and Outcomes Frameworks to incentivise timely routine childhood vaccinations in primary care.(39) In London a primary care led quality improvement programme has been launched to tackle inequalities in timeliness of routine preschool childhood vaccinations.(40)

Conclusion

Routine vaccination schedules have been disrupted during the COVID-19 pandemic. Our study adds important new evidence of the impact on timeliness of MMR vaccinations, and demonstrates unwarranted variation by neighbourhood, ethnicity and deprivation. This data provides further evidence to prioritise quality improvement and catch-up campaigns to achieve herd immunity and prevent measles outbreaks. It provides actionable information in populations and geographies experiencing significant health inequalities.

Acknowledgements

We are grateful to colleagues within the Clinical Effectiveness Group for access to and expertise in using general practice data. We are also grateful to Professor Bianca de Stavola (Great Ormond Street Institute of Child Health) for her statistical guidance and to Professor Helen Bedford (Great Ormond Street Institute of Child Health) for her clinical expertise and guidance. This work uses data provided by patients and collected by the NHS as part of their care and support. We would also like to acknowledge our PPI participants for their invaluable insights into patient perspectives around dissemination of our research to the wider community.

Contributor and guarantor information

CD and JR obtained funding for the study. NF and CD conceptualised and designed the analyses. ID and ZA provided guidance for specifying clinical codes. CW and KH extracted data. MM carried out the literature search. NF conducted the analyses, generated tables and figures and drafted the initial manuscript. GH produced the choropleth maps. All authors contributed to the interpretation of analyses and reviewed and revised the manuscript. All authors were involved in writing the paper and had final approval of the submitted and published manuscript. The corresponding authors attest that all listed authors meet authorship criteria and that no others meeting the criteria have been omitted. CD is the guarantor and accepts full responsibility for the conduct of the study, had access to the data, and controlled the decision to publish.

Funding

This research was funded by a grant from Barts Charity ref: MGU0419. This work was supported by Health Data Research UK, which is funded by the UK Medical Research Council, Engineering and Physical Sciences Research Council, Economic and Social Research Council, Department of Health and Social Care (England), Chief Scientist Office of the Scottish Government Health and Social Care Directorates, Health and Social Care Research and Development Division (Welsh Government), Public Health Agency (Northern Ireland), British

For peer review only - http://bmjopen.bmj.com/site/about/guidelines.xhtml

Heart Foundation and Wellcome. MM is supported by a locally funded National Institute for Health Research Academic Clinical Fellowship. The funders had no role in study design, data collection and analysis, decision to publish, or preparation of the manuscript.

Competing interests declaration

All authors have completed the ICMJE uniform disclosure form at http://www.icmje.org/disclosure-of-interest/ and declare: no support from any organisation for the submitted work; no financial relationships with any organisations that might have an interest in the submitted work in the previous three years; no other relationships or activities that could appear to have influenced the submitted work.

Ethics approval

This study was a service evaluation (measuring what standard of care this service achieved) and analysed routinely acquired de-identified data: hence no research ethics committee approval was required by the Health Research Authority. Access to general practice data is enabled by data sharing agreements between the Discovery Data Service and general practice data controllers. The Discovery Programme Board has approved data access by the REAL Child Health programme.

Data sharing

Access to general practice data is enabled by data sharing agreements between the Discovery Data Service and general practice data controllers. The Discovery Programme Board has approved data access by the REAL Child Health programme team for research on the condition that it is not onwardly shared.

Transparency statement

The corresponding author affirms that the manuscript is an honest, accurate, and transparent account of the study being reported; that no important aspects of the study have been omitted; and that any discrepancies from the study as planned (and, if relevant, registered) have been explained.

Copyright/license for publication

The corresponding author has the right to grant on behalf of all authors and does grant on behalf of all authors, a worldwide licence to the Publishers and its licensees in perpetuity, in all forms, formats and media (whether known now or created in the future), to i) publish, reproduce, distribute, display and store the Contribution, ii) translate the Contribution into other languages, create adaptations, reprints, include within collections and create summaries, extracts and/or, abstracts of the Contribution, iii) create any other derivative work(s) based on the Contribution, iv) to exploit all subsidiary rights in the Contribution, v) the inclusion of electronic links from the Contribution to third party material where-ever it may be located; and, vi) licence any third party to do any or all of the above.

References

1. Saxena S, Skirrow H, Bedford H. Routine vaccination during covid-19 pandemic response. Bmj. 2020;369:m2392.

2. Public Health England. Impact of physical distancing measures due to COVID-19 pandemic in England on childhood vaccination counts up to week 41, and vaccine coverage up to August 2020. 2020. Contract No.: 20.

For peer review only - http://bmjopen.bmj.com/site/about/guidelines.xhtml

3. NHS Digital. Childhood Vaccination Coverage Statistics - 2020-21 2022 [Available from: https://digital.nhs.uk/data-and-information/publications/statistical/nhs-immunisation-statistics/england---2020-21.

4. Bell S, Clarke R, Paterson P, Mounier-Jack S. Parents' and guardians' views and experiences of accessing routine childhood vaccinations during the coronavirus (COVID-19) pandemic: A mixed methods study in England. PLOS ONE. 2021;15(12):e0244049.

5. Keenan A, Ghebrehewet S, Vivancos R, Seddon D, MacPherson P, Hungerford D. Measles outbreaks in the UK, is it when and where, rather than if? A database cohort study of childhood population susceptibility in Liverpool, UK. BMJ Open. 2017;7(3):e014106.

6. National Audit Office. Investigation into pre-school vaccinations: National Audit Office; 2019 [Available from: https://www.nao.org.uk/report/investigation-into-pre-school-vaccinations/.

7. Spencer N, Nathawad R, Arpin E, Johnson S. Pandemics, epidemics and inequities in routine childhood vaccination coverage: a rapid review. BMJ Paediatrics Open. 2020;4(1):e000842.

8. Tiley KS, White JM, Andrews N, Ramsay M, Edelstein M. Inequalities in childhood vaccination timing and completion in London. Vaccine. 2018;36(45):6726-35.

9. O'Leary ST, Trefren L, Roth H, Moss A, Severson R, Kempe A. Number of Childhood and Adolescent Vaccinations Administered Before and After the COVID-19 Outbreak in Colorado. JAMA Pediatrics. 2020:305–7.

10. British Medical Association, National Health Service. Quality and Outcomes Framework guidance for 2021/22 2021 [Available from: https://www.england.nhs.uk/wp-content/uploads/2021/03/B0456-update-on-quality-outcomes-framework-changes-for-21-22-.pdf.

11. Ministry of Housing Communities & Local Government. The English Indices of Deprivation 2019 - Frequently Asked Questions (FAQs) 2016 [Available from: https://assets.publishing.service.gov.uk/government/uploads/system/uploads/attachment_data/file/853811/IoD2019_FAQ_v4.pdf.

12. NIHR Centre for Engagement and Dissemination. UK Standards for Public Involvement 2022 [Available from: https://sites.google.com/nihr.ac.uk/pi-standards/standards?authuser=0.

13. Moreno-Montoya J, Ballesteros SM, Rojas Sotelo JC, Bocanegra Cervera CL, Barrera-López P, De la Hoz-Valle JA. Impact of the COVID-19 pandemic on routine childhood immunisation in Colombia. Arch Dis Child. 2021;107(3):e4.

14. Jain R, Chopra A, Falézan C, Patel M, Dupas P. COVID-19 related immunization disruptions in Rajasthan, India: A retrospective observational study. Vaccine. 2021;39(31):4343-50.

15. Aizawa Y, Katsuta T, Sakiyama H, Tanaka-Taya K, Moriuchi H, Saitoh A. Changes in childhood vaccination during the coronavirus disease 2019 pandemic in Japan. Vaccine. 2021;39(29):4006-12.

16. Silveira MF, Tonial CT, Goretti KMA, Teixeira AMS, Hallal PC, Maria BMA, et al. Missed childhood immunizations during the COVID-19 pandemic in Brazil: Analyses of routine statistics and of a national household survey. Vaccine. 2021;39(25):3404-9.

17. Shen AK, Bramer CA, Kimmins LM, Swanson R, Vranesich P, Orenstein W. Vaccine Coverage Across the Life Course in Michigan During the COVID-19 Pandemic: January–September 2020. Am J Public Health. 2021;111(11):2027-35.

18. Bhadoria SS, Shukla DK, Joshi A. COVID-19 Pandemic: A Speed Breaker for Routine Immunisation. Journal of Clinical and Diagnostic Research. 2021;15(4):LC32-LC5.

19. Causey K, Fullman N, Sorensen RJD, Galles NC, Zheng P, Aravkin A, et al. Estimating global and regional disruptions to routine childhood vaccine coverage during the COVID-19 pandemic in 2020: a modelling study. Lancet. 2021;398(10299):522-34.

20. Piché-Renaud PP, Ji C, Farrar DS, Friedman JN, Science M, Kitai I, et al. Impact of the COVID-19 pandemic on the provision of routine childhood immunizations in Ontario, Canada. Vaccine. 2021;39(31):4373-82.

21. Huss G, Magendie C, Pettoello-Mantovani M, Jaeger-Roman E. Implications of the COVID-19 Pandemic for Pediatric Primary Care Practice in Europe. The Journal of Pediatrics. 2021;233:290-1.e2.

For peer review only

For peer review only - http://bmjopen.bmj.com/site/about/guidelines.xhtml

22. Shet A, Dhaliwal B, Banerjee P, DeLuca A, Carr K, Britto C, et al. Childhood immunisations in India during the COVID-19 pandemic. BMJ Paediatrics Open. 2021;5(1):e001061.
23. Bielecki K, Kirolos A, Willocks LJ, Pollock KG, Gorman DR. Low uptake of nasal influenza vaccine in Polish and other ethnic minority children in Edinburgh, Scotland. Vaccine. 2019;37(5):693-7.
24. Baker D, Garrow A, Shiels C. Inequalities in immunisation and breast feeding in an ethnically diverse urban area: cross-sectional study in Manchester, UK. J Epidemiol Community Health. 2011;65(4):346-52.
25. Gaughan CH, Razieh C, Khunti K, Banerjee A, Chudasama YV, Davies MJ, et al. COVID-19 vaccination uptake amongst ethnic minority communities in England: a linked study exploring the drivers of differential vaccination rates. Journal of Public Health. 2022;fdab400.
26. Hoang U, de Lusignan S, Joy M, Sherlock J, Williams J, Bankhead C, et al. National rates and disparities in childhood vaccination and vaccine-preventable disease during the COVID-19 pandemic: English sentinel network retrospective database study. Arch Dis Child. 2022.
27. Haider EA, Willocks LJ, Anderson N. Identifying inequalities in childhood immunisation uptake and timeliness in southeast Scotland, 2008-2018: A retrospective cohort study. Vaccine. 2019;37(37):5614-24.
28. Cockman P, Dawson L, Mathur R, Hull S. Improving MMR vaccination rates: herd immunity is a realistic goal. BMJ. 2011;343:d5703.
29. Henderson L, Millett C, Thorogood N. Perceptions of childhood immunization in a minority community: qualitative study. Journal of the Royal Society of Medicine. 2008;101(5):244-51.
30. Letley L, Rew V, Ahmed R, Habersaat KB, Paterson P, Chantler T, et al. Tailoring immunisation programmes: Using behavioural insights to identify barriers and enablers to childhood immunisations in a Jewish community in London, UK. Vaccine. 2018;36(31):4687-92.
31. Durrheim DN, Andrus JK, Tabassum S, Bashour H, Githanga D, Pfaff G. A dangerous measles future looms beyond the COVID-19 pandemic. Nat Med. 2021;27(3):360-1.
32. Feldman AG, O'Leary ST, Danziger-Isakov L. The Risk of Resurgence in Vaccine-Preventable Infections Due to Coronavirus Disease 2019-Related Gaps in Immunization. Clin Infect Dis. 2021;73(10):1920-3.
33. Olusanya OA, Bednarczyk RA, Davis RL, Shaban-Nejad A. Addressing Parental Vaccine Hesitancy and Other Barriers to Childhood/Adolescent Vaccination Uptake During the Coronavirus (COVID-19) Pandemic. Front Immunol. 2021;12:663074.
34. Kadambari S, Goldacre R, Morris E, Goldacre MJ, Pollard AJ. Indirect effects of the covid-19 pandemic on childhood infection in England: population based observational study. BMJ. 2022;376:e067519.
35. Bedford H, Donovan H. We need to increase MMR vaccine uptake urgently. BMJ. 2022;376:o818.
36. Guerra FM, Bolotin S, Lim G, Heffernan J, Deeks SL, Li Y, et al. The basic reproduction number (R0) of measles: a systematic review. The Lancet Infectious Diseases. 2017;17(12):e420-e8.
37. MacDonald NE, Comeau JL, Dube E, Bucci LM. COVID-19 and missed routine immunizations: designing for effective catch-up in Canada. Can J Public Health. 2020;111(4):469-72.
38. World Health Organization. Guiding principles for immunization activities during the COVID-19 pandemic: interim guidance, 26 March 2020 Geneva: World Health Organization; 2020 [updated 2020. Available from: https://apps.who.int/iris/handle/10665/331590.
39. British Medical Association, NHS England. Update to the GP contract agreement 2020/21 - 2023/24 2020 [Available from: https://www.england.nhs.uk/wp-content/uploads/2020/03/update-to-the-gp-contract-agreement-v2-updated.pdf.
40. Clinical Effectiveness Group Queen Mary University of London. Preventing future measles outbreaks: Queen Mary University of London; 2021 [Available from: https://www.qmul.ac.uk/blizard/ceg/about-us/case-studies/preventing-future-measles-outbreaks/.

For peer review only - http://bmjopen.bmj.com/site/about/guidelines.xhtml

443 *Figure 1 – Study sample*

444

445 *Figure 2a – Proportion of children receiving timely MMR vaccination in the pre-pandemic cohort, by 2011*
446 *Lower Super Output Area*[1]

447 [1] *Lower super output area of the child's home address, as recorded in their general practice electronic health*
448 *record.*

449

450 *Figure 2b – Proportion of children receiving timely MMR vaccination in the pandemic cohort, by 2011 Lower*
451 *Super Output Area*[1]

452 [1] *Lower super output area of the child's home address, as recorded in their general practice electronic health*
453 *record.*

454

455 *Figure 3 – Change*[1] *in the proportion of children receiving timely MMR vaccination between pre-pandemic and*
456 *pandemic cohorts, by 2011 Lower Super Output Area*[2]

457 [1] *Proportion receiving timely MMR vaccination in pandemic cohort minus the proportion receiving timely MMR*
458 *vaccination in the pre-pandemic cohort.* [2] *Lower super output area of the child's home address, as recorded in*
459 *their general practice electronic health record.*

460

461 *Figure 4 – Adjusted log odds of timely MMR vaccination*[1]

462 [1] *Model mutual adjusting for cohort, sex, Clinical Commissioning Group, ethnic background and Index of*
463 *Multiple Deprivation quintile.* [2] *Odds ratio.* [3] *95% confidence interval.*

464

465 *Supplementary file 1 – Study protocol*

466

467 *Supplementary file 2 – STROBE checklist*

468

469 *Supplementary file 3 – Additional tables and figures*

470

For peer review only

For peer review only - http://bmjopen.bmj.com/site/about/guidelines.xhtml

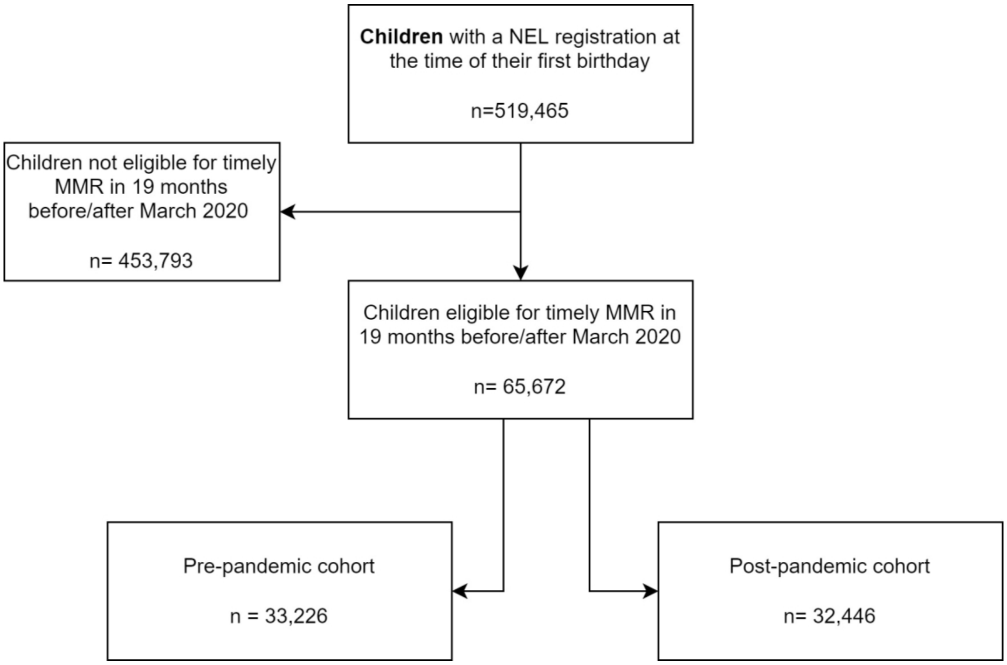

Figure 1 – Study sample

614x403mm (72 x 72 DPI)

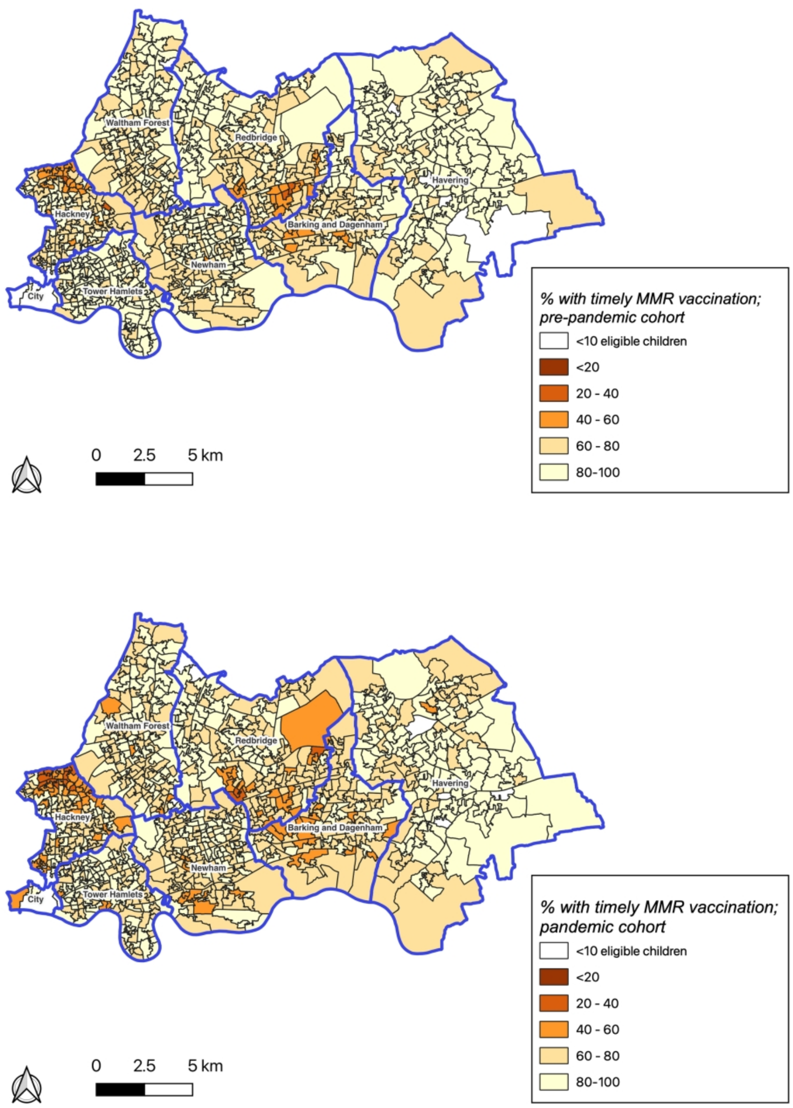

Figure 2a – Proportion of children receiving timely MMR vaccination in the pre-pandemic cohort, by 2011 Lower Super Output Area[1]

Figure 2b – Proportion of children receiving timely MMR vaccination in the pandemic cohort, by 2011 Lower Super Output Area[1]

209x296mm (300 x 300 DPI)

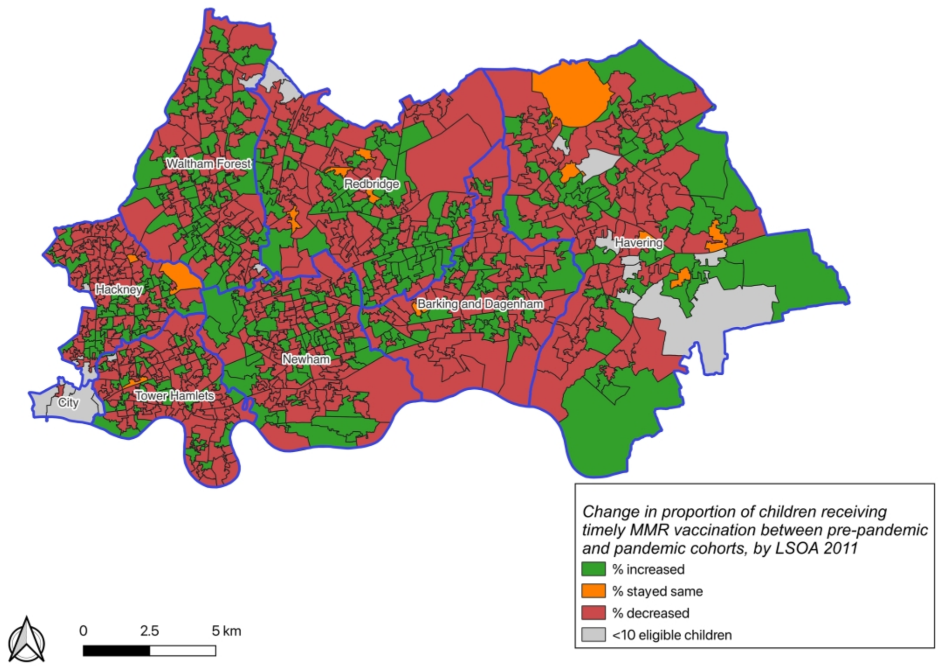

Figure 3 – Change[1] in the proportion of children receiving timely MMR vaccination between pre-pandemic and pandemic cohorts, by 2011 Lower Super Output Area[2]

296x209mm (300 x 300 DPI)

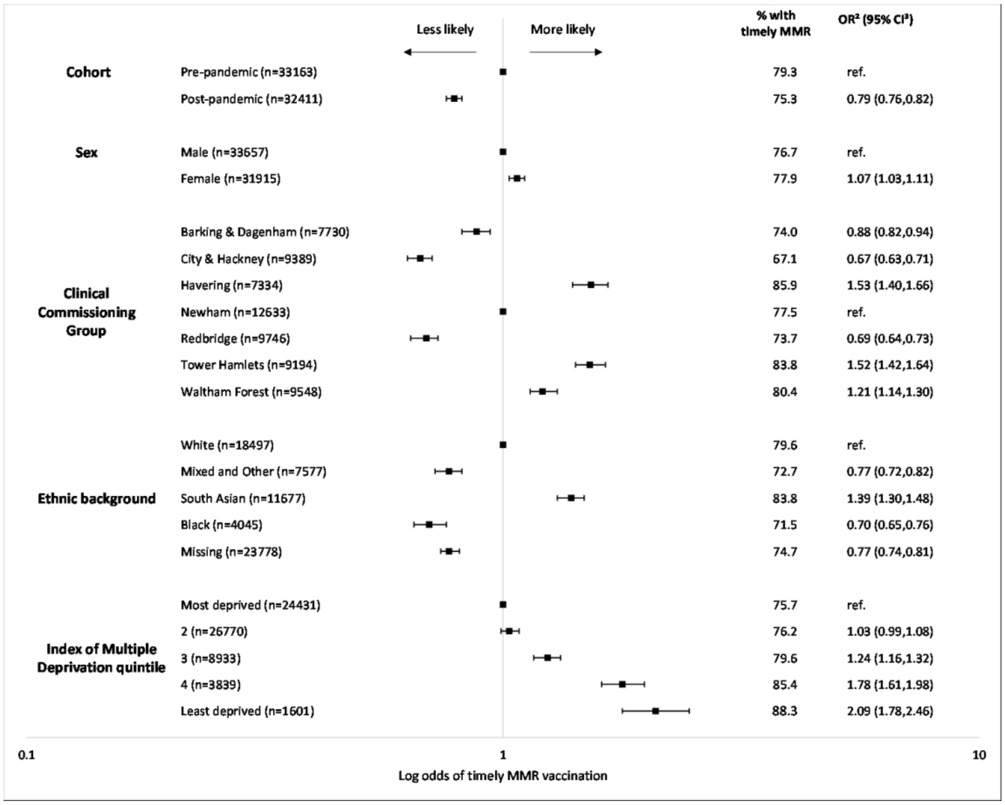

Figure 4 – Adjusted log odds of timely MMR vaccination[1]

318x254mm (118 x 118 DPI)

COVID-19 and childhood MMR immunisation study protocol

Preliminary title: Assessing the impact of the COVID-19 pandemic on timeliness of MMR uptake in east London: cross-sectional analysis of primary care electronic health records

Writing group: Nicola Firman, Milena Marszalek, Ana Gutierrez, Kate Homer, Crystal Williams, Gill Harper, Isabel Dostal, Zaheer Ahmed, John Robson, Carol Dezateux

Background/rationale

The COVID-19 pandemic has disrupted routine health care and services across the UK, through rising COVID infections as well as the introduction of social distancing measures and lockdowns.(1) The United Kingdom (UK) Joint Committee on Vaccination and Immunisation (JCVI) has emphasised the importance of children continuing to receive routine childhood vaccinations according to the national schedule throughout periods of lockdown.(2)

In the UK, childhood vaccine coverage is routinely assessed quarterly using the COVER (Cover of vaccination evaluated rapidly) programme for children who have reached their first, second, or fifth birthdays. Given the timing of data extractions for COVER, the UK Health Security Agency (HAS) has indicated that this system cannot provide reliable information of the impact of the pandemic on vaccine coverage for England. However, evidence suggests that vaccine coverage measured at 24 months among children scheduled to receive Measles, Mumps and Rubella (MMR) vaccination from March 2020 onwards in England was 90.3%, around 0.3% lower than 2019, with average levels in both years well below the WHO coverage target of 95%.(2, 3) This is broadly in line with data from approximately 38% of GP practices in England, which suggests that vaccination counts for MMR vaccine were 2.1% lower by the end of 2020 than for the equivalent period in 2019. Qualitative studies in the UK looking at attitudes towards routine vaccination during the pandemic report parental fear of attending routine vaccination appointments, reduced concern about catching vaccine-preventable diseases during lockdown, and routine appointment re-scheduling. These findings support emerging research that demonstrates disruption across routine child vaccination schedules.(4)

The measures currently used lack information on vaccination timeliness, impacts on existing vaccine coverage inequalities and do not include areas of historic MMR low uptake. This is important as it has been acknowledged that area-level averages conceal geographical clusters of susceptibility which fuel outbreaks.(5) A systematic review found that there is currently no published literature looking at both routine vaccination schedule disruption and consequential impacts on vaccination inequalities during pandemics.(6) More specific literature exists around uptake trends for particular vaccinations - Macdonald et al. demonstrated that MMR uptake during April 2020 dropped by 42·5% in London in comparison to the same time period during 2019.(7) Overall, MMR uptake decreased by 19.8% in England, before recovering over the following months.(8) Most recent local authority coverage data demonstrates that on London, MMR rates have fallen from 83.6% to 82.4% at 24 months from 2019-2021.(9) Areas previously demonstrating lower MMR uptake were worst affected, including London and the Midlands.(7) UK Cover of vaccination evaluated rapidly (COVER) reports look at vaccine coverage at 12, 24 months, and five years, but do not encompass timeliness measures.

These trends reflect pre-existing inequalities observed in MMR uptake in the UK.(10) Historically, the UK lost its measles 'elimination status' in 2019 due to consistently low MMR uptake.(11) Further reduction in MMR vaccinations will increase the risk of measles outbreaks, particularly in London where a significant proportion of children start school without the full protection offered by MMR vaccination.(12)

Objectives

This protocol sets out to understand the wider impact of COVID-19 on timeliness of MMR vaccinations in seven east London Clinical Commissioning Groups (CCGs). Specifically:

1. To understand the impact of COVID-19 and periods of lockdown on MMR vaccination timeliness
2. To describe geographical clustering of MMR vaccination timeliness
3. To investigate if COVID-19 has amplified inequalities in receipt of timely MMR vaccination

For peer review only

Methods

Study design

A cross-sectional study using primary care electronic health records (EHRs) from child patients registered at 285 general practices in seven geographically contiguous east London CCGs: Barking & Dagenham, City & Hackney, Havering, Newham, Redbridge, Tower Hamlets, and Waltham Forest.

Study sample

The study population will include all children eligible to receive their first MMR vaccination at age 12 to 18 months old, respectively, in the 19 months before and after 23rd March 2020. All children ever registered at one of the 285 general practices between September 2001 and October 2021 will be included.

Data

Data will be extracted from the North East London (NEL) Discovery Data Service (DDS) which receives primary care electronic health record data on a daily basis from all general practices in NEL, on 23rd November 2021. Events recorded up until 1st November 2021 will be included. All data will be extracted and managed according to UK NHS information governance requirements.

We will analyse person-level data for children born between 22nd August 2017 and 22nd September 2018 (pre-pandemic cohort) and 23rd March 2019 and 1st May 2020 (pandemic cohort). These children would be eligible to receive timely MMR vaccination between 12 and 18 months of age in the 19 months before and after England entered the first lockdown on 23rd March 2020, respectively. For each child, the person-level dataset will contain: a pseudonymised person identifier; calendar week, month and year of birth; ethnic background; sex; 2011 lower super output area (LSOA) of the child's address; CCG; MMR clinical code (Read or SNOMED); and date of MMR vaccination. To avoid disclosure of identifiable data, a proxy date of birth will be derived by assigning the week commencing date using the calendar week and year of birth. Consequently, date of birth could be up to six days earlier than the child's actual date of birth. We will merge 2015 Index of Multiple Deprivation (IMD) score and rank(17) into the datafile using LSOA as the linkage field, and categorise IMD rank into quintiles from most to least deprived.

Following guidance from the Green Book, MMR vaccinations given before a child's first birthday will be ignored.(18) Where available, subsequent MMR records will be treated as the child's first vaccination, otherwise early MMR vaccinations will be retained in the dataset and later considered as "not timely".

The following records will be excluded from analyses:

- Records with an MMR clinical code but no date
- Exact duplicate records
- Duplicate records where the date is the same but MMR clinical code differs
- Latter of multiple MMR events

Statistical methods

We will identify timely MMR vaccination among children aged between 12 and 18 months when they received their MMR vaccination. We will explore variation in the proportion of children receiving timely MMR vaccination by cohort, sex, CCG, ethnic background and IMD quintile, and investigate differences in the proportion of children receiving timely MMR vaccination in each cohort, by these covariates.

To describe geographical clustering of MMR vaccination timeliness we will plot in choropleth maps the proportion of children receiving timely MMR vaccination in each cohort, by LSOA.

We will conduct binary logistic regression to estimate the odds (odds ratio [OR] and 95% CI) of timely MMR vaccination after adjustment for covariates and, to assess whether COVID-19 has widened inequalities in timely vaccination, explore an interaction between cohort and IMD quintile.

For peer review only

Ethics

This protocol describes analyses of deidentified data for audit purposes and therefore no REC approval is required.

Strengths and limitations

Strengths of this study include:

- Use of person-level data to comprehensively document the impact of COVID-19 on timely MMR vaccine uptake and coverage
- EHR from seven geographically contiguous CCGs, serving a whole population with a large sample size, including children who have left/deregistered from a practice in NEL, or died
- Access to historic data "pre-pandemic" cohort to use as control group
- Implementation of robust statistical methods

Limitations of this study include:

- Use of calendar week of birth to generate a proxy date of birth
- Primary purpose of EHRs is not for research – so it is possible there will be some data quality issues
    - Large proportions of children with missing ethnic background recorded in their EHR

Funding

This research was funded by a grant from Barts Charity ref: MGU0419. This work was supported by Health Data Research UK (award reference: GPPB1C2), which is funded by the UK Medical Research Council, Engineering and Physical Sciences Research Council, Economic and Social Research Council, Department of Health and Social Care (England), Chief Scientist Office of the Scottish Government Health and Social Care Directorates, Health and Social Care Research and Development Division (Welsh Government), Public Health Agency (Northern Ireland), British Heart Foundation and Wellcome.

References

1. Saxena S, Skirrow H, Bedford H. Routine vaccination during covid-19 pandemic response. BMJ. 2020;369:m2392.
2. Public Health England. Impact of physical distancing measures due to COVID-19 pandemic in England on childhood vaccination counts up to week 41, and vaccine coverage up to August 2020. 2020. Contract No.: 20.
3. Public Health England. Impact of COVID-19 on childhood vaccination counts to week 47, and vaccine coverage to October 2020 in England: interim analyses. Public Health England; 2020 8 December 2020. Contract No.: 23.
4. O'Leary ST, Trefren L, Roth H, Moss A, Severson R, Kempe A. Number of Childhood and Adolescent Vaccinations Administered Before and After the COVID-19 Outbreak in Colorado. JAMA Pediatrics. 2020.
5. Spencer N, Nathawad R, Arpin E, Johnson S. Pandemics, epidemics and inequities in routine childhood vaccination coverage: a rapid review. BMJ Paediatrics Open. 2020;4(1).
6. McDonald HI, Tessier E, White JM, Woodruff M, Knowles C, Bates C, et al. Early impact of the coronavirus disease (COVID-19) pandemic and physical distancing measures on routine childhood vaccinations in England, January to April 2020. Euro Surveill. 2020;25(19).
7. Public Health England. Quarterly vaccination coverage statistics for children aged up to 5 years in the UK (COVER programme): April to June 2020. 2020. Contract No.: 18.
8. Bramer CA, Kimmins LM, Swanson R, Kuo J, Vranesich P, Jacques-Carroll LA, et al. Decline in Child Vaccination Coverage During the COVID-19 Pandemic — Michigan Care Improvement Registry, May 2016–May 2020. Morbidity and Mortality Weekly Report. 2020;69(20):630-1.
9. Chandir S, Siddiqi DA, Setayesh H, Khan AJ. Impact of COVID-19 lockdown on routine immunisation in Karachi, Pakistan. The Lancet Global Health. 2020;8(9):e1118-e20.

For peer review only

10. Bechini A, Garamella G, Giammarco B, Zanella B, Flori V, Bonanni P, et al. Paediatric activities and adherence to vaccinations during the COVID-19 epidemic period in Tuscany, Italy: a survey of paediatricians. J Prev Med Hyg. 2020;61(2):E125-E9.
11. Alsuhaibani M, Alaqeel A. Impact of the COVID-19 Pandemic on Routine Childhood Immunization in Saudi Arabia. Vaccines (Basel). 2020;8(4).
12. MacDonald NE, Comeau JL, Dube E, Bucci LM. COVID-19 and missed routine immunizations: designing for effective catch-up in Canada. Can J Public Health. 2020;111(4):469-72.
13. Bell SL, Clarke R, Paterson P, Mounier-Jack S. Parents' and guardians' views and experiences of accessing routine childhood vaccinations during the coronavirus (COVID-19) pandemic: A mixed-methods study in England 2020.
14. Keenan A, Ghebrehewet S, Vivancos R, Seddon D, MacPherson P, Hungerford D. Measles outbreaks in the UK, is it when and where, rather than if? A database cohort study of childhood population susceptibility in Liverpool, UK. BMJ Open. 2017;7(3):e014106.
15. National Audit Office. Investigation into pre-school vaccinations. 2019. Contract No.: 007836.
16. Tiley KS, White JM, Andrews N, Ramsay M, Edelstein M. Inequalities in childhood vaccination timing and completion in London. Vaccine. 2018;36(45):6726-35.
17. Department for Communities and Local Government. The English Indices of Deprivation 2015 - Frequently Asked Questions (FAQs). 2016.
18. Public Health England. Measles: the green book, chapter 21. In: Public Health England, editor. The Green Book2019.

For peer review only

STROBE 2007 (v4) Statement—Checklist of items that should be included in reports of *cross-sectional studies*

Section/Topic	Item #	Recommendation	Reported on page #
**Title and abstract**	1	(*a*) Indicate the study's design with a commonly used term in the title or the abstract	1
		(*b*) Provide in the abstract an informative and balanced summary of what was done and what was found	2
**Introduction**			
Background/rationale	2	Explain the scientific background and rationale for the investigation being reported	4
Objectives	3	State specific objectives, including any prespecified hypotheses	4
**Methods**			
Study design	4	Present key elements of study design early in the paper	4
Setting	5	Describe the setting, locations, and relevant dates, including periods of recruitment, exposure, follow-up, and data collection	4
Participants	6	(*a*) Give the eligibility criteria, and the sources and methods of selection of participants	4
Variables	7	Clearly define all outcomes, exposures, predictors, potential confounders, and effect modifiers. Give diagnostic criteria, if applicable	5
Data sources/ measurement	8*	For each variable of interest, give sources of data and details of methods of assessment (measurement). Describe comparability of assessment methods if there is more than one group	5
Bias	9	Describe any efforts to address potential sources of bias	5
Study size	10	Explain how the study size was arrived at	4-5
Quantitative variables	11	Explain how quantitative variables were handled in the analyses. If applicable, describe which groupings were chosen and why	5
Statistical methods	12	(*a*) Describe all statistical methods, including those used to control for confounding	5
		(*b*) Describe any methods used to examine subgroups and interactions	5
		(*c*) Explain how missing data were addressed	5
		(*d*) If applicable, describe analytical methods taking account of sampling strategy	n/a
		(*e*) Describe any sensitivity analyses	n/a
**Results**			

For peer review only

Participants	13*	(a) Report numbers of individuals at each stage of study—eg numbers potentially eligible, examined for eligibility, confirmed eligible, included in the study, completing follow-up, and analysed	5 and Figure 1
		(b) Give reasons for non-participation at each stage	5
		(c) Consider use of a flow diagram	Figure 1
Descriptive data	14*	(a) Give characteristics of study participants (eg demographic, clinical, social) and information on exposures and potential confounders	6
		(b) Indicate number of participants with missing data for each variable of interest	Table 1
Outcome data	15*	Report numbers of outcome events or summary measures	6 and Table 1
Main results	16	(*a*) Give unadjusted estimates and, if applicable, confounder-adjusted estimates and their precision (eg, 95% confidence interval). Make clear which confounders were adjusted for and why they were included	6 and Table S5 and Figure 4
		(*b*) Report category boundaries when continuous variables were categorized	n/a
		(*c*) If relevant, consider translating estimates of relative risk into absolute risk for a meaningful time period	n/a
Other analyses	17	Report other analyses done—eg analyses of subgroups and interactions, and sensitivity analyses	6
**Discussion**			
Key results	18	Summarise key results with reference to study objectives	6-7
Limitations	19	Discuss limitations of the study, taking into account sources of potential bias or imprecision. Discuss both direction and magnitude of any potential bias	7
Interpretation	20	Give a cautious overall interpretation of results considering objectives, limitations, multiplicity of analyses, results from similar studies, and other relevant evidence	7-8
Generalisability	21	Discuss the generalisability (external validity) of the study results	7-8
**Other information**			
Funding	22	Give the source of funding and the role of the funders for the present study and, if applicable, for the original study on which the present article is based	9

*Give information separately for cases and controls in case-control studies and, if applicable, for exposed and unexposed groups in cohort and cross-sectional studies.

Note: An Explanation and Elaboration article discusses each checklist item and gives methodological background and published examples of transparent reporting. The STROBE checklist is best used in conjunction with this article (freely available on the Web sites of PLoS Medicine at http://www.plosmedicine.org/, Annals of Internal Medicine at http://www.annals.org/, and Epidemiology at http://www.epidem.com/). Information on the STROBE Initiative is available at www.strobe-statement.org.

For peer review only

Impact of the COVID-19 pandemic on timeliness and equity of measles, mumps and rubella vaccinations in north east London: a longitudinal study using electronic health records

Nicola Firman[1*], Milena Marszalek[1*], Ana Gutierrez[1], Kate Homer[1], Crystal Williams[1], Gill Harper[1], Isabel Dostal[1], Zaheer Ahmed[1], John Robson[1], Carol Dezateux[1]

[1] Centre for Primary Care, Wolfson Institute of Population Health, Barts and the London School of Medicine and Dentistry, Queen Mary University of London, Yvonne Carter Building, 58 Turner Street, London E1 2AB

*Joint corresponding authors. Nicola Firman (nicola.firman@qmul.ac.uk) and Dr Milena Marszalek (m.marszalek@qmul.ac.uk). Centre for Primary Care, Wolfson Institute of Population Health, Barts and the London School of Medicine and Dentistry, Queen Mary University of London, Yvonne Carter Building, 58 Turner Street, London, E1 2AB

Supplementary tables and figures

For peer review only

Figure S1 – Identifying registrations at the time of first birthday

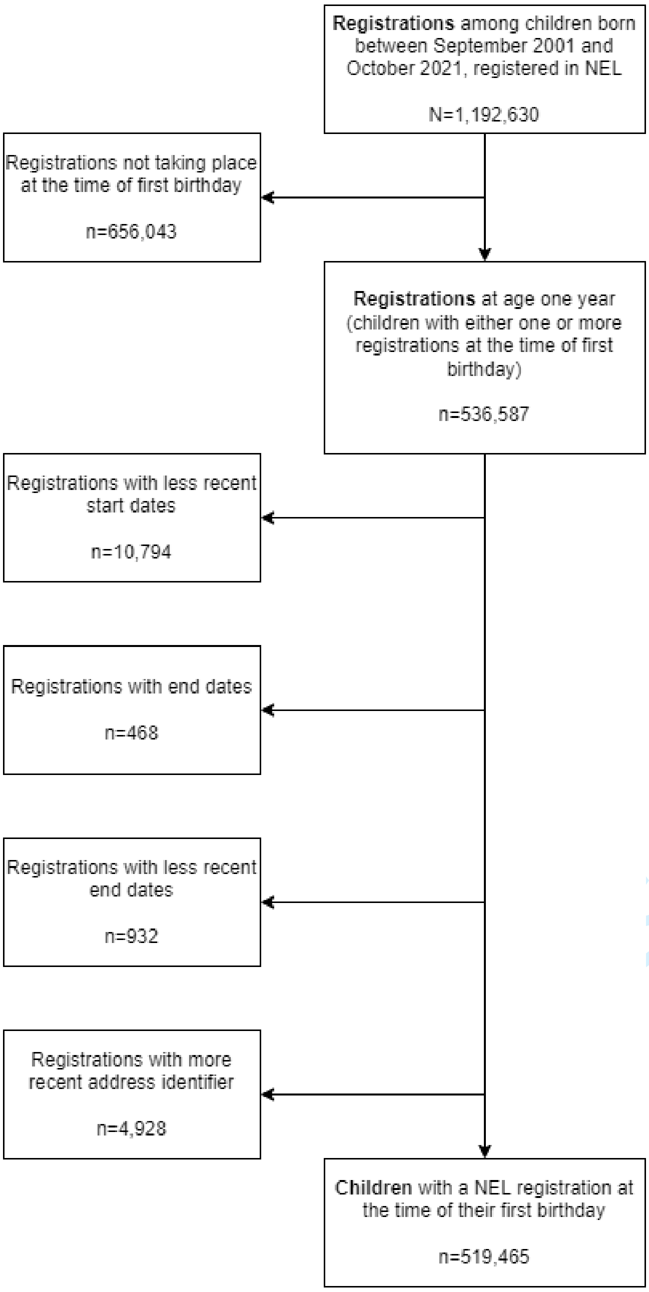

iew only

We excluded 656,043 (55.0% of 1,192,630) registrations, which were not current at 12 months of age. For children with multiple concurrent registrations at the time of their first birthday, we applied the following sequence of hierarchical rules to determine which registration should be retained:

Retain

1. *registration with the most recent start date*
2. *registration with a NULL end date (considered more recent/ongoing)*
3. *registration with the most recent end date*
4. *registration with the lowest address identification number (children may have multiple registrations starting and ending on the same date as a result of practice mergers and closures. The lower address identifier is assumed to relate to the original registration)*

Figure S2 – Processing the MMR data file

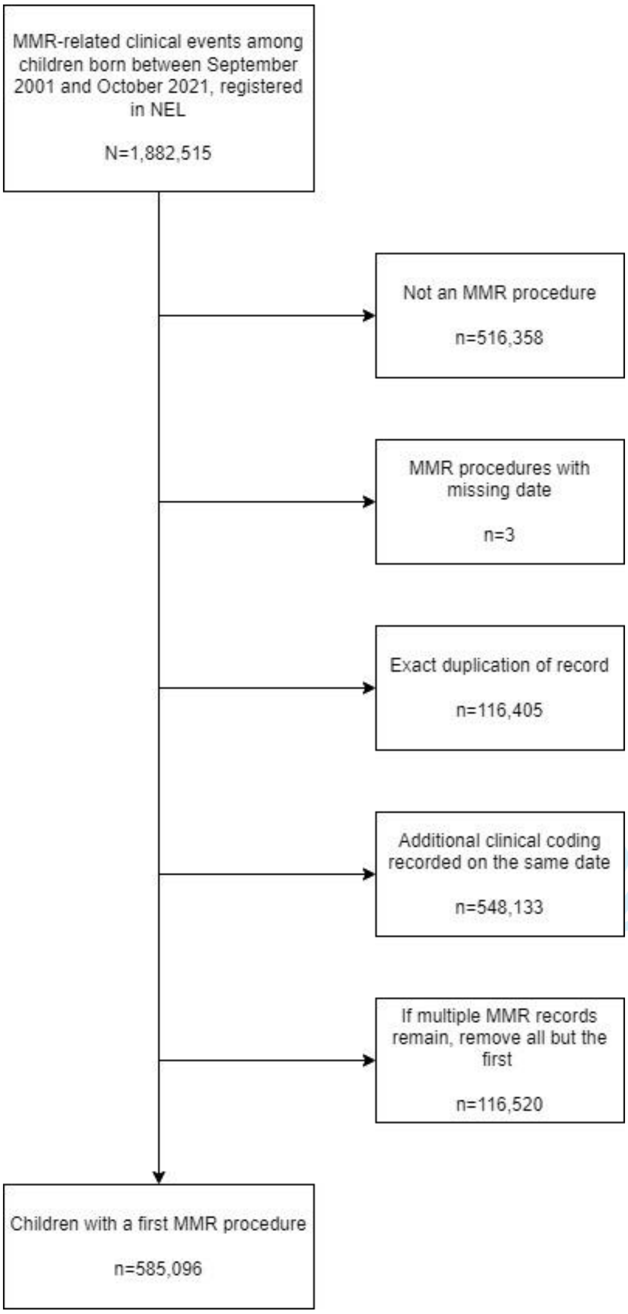

Data relating to MMR vaccinations were extracted separately. The data file was in long format where each row was a unique clinical code, therefore children often have multiple rows indicating various MMR administrative processes and procedures throughout their life. For the purposes of this study, it was necessary to identify the first MMR procedure for each child. Of 1,882,515 MMR records, 516,358 records were removed as they were not relating to MMR procedures (see figure S1 and table S1). Records without a date (n=3), as well as duplicate records were removed (n=664,538). Finally, all but the earliest event if a child had more than one MMR vaccination recorded were removed (n=116,520). MMR vaccinations given before the child's first birthday were excluded if the child received another dose after turning 12 months of age, as per guidance from The Green Book.[1] Where available, subsequent MMR records were treated as the child's first vaccination, otherwise early MMR vaccinations were retained in the dataset and later considered as "not timely".

[1] Public Health England. Measles: the green book, chapter 21. In: Public Health England, ed. The Green Book; 2019.

Table S1 - SNOMED clinical codes for MMR procedures

Code term	SNOMED Concept ID
Administration of vaccine product containing only Measles morbillivirus and Mumps orthorubulavirus and Rubella virus antigens (procedure)	38598009
Administration of vaccine product containing only Measles morbillivirus antigen (procedure)	47435007
Administration of vaccine product containing only Mumps orthorubulavirus antigen (procedure)	50583002
Administration of vaccine product containing only Rubella virus antigen (procedure)	82314000
Measles vaccination plus immunoglobulin (procedure)	170364006
Administration of vaccine product containing only Human alphaherpesvirus 3 and Measles morbillivirus and Mumps orthorubulavirus and Rubella virus antigens (procedure)	432636005
Administration of first dose of vaccine product containing only Human alphaherpesvirus 3 and Measles morbillivirus and Mumps orthorubulavirus and Rubella virus antigens (procedure)	871909005
Measles, mumps and rubella vaccination given (situation)	150971000119104
Measles mumps and rubella vaccination - first dose (procedure)	308081000000105
Measles mumps rubella catch-up vaccination (procedure)	505001000000109
Administration of vaccine product containing only live attenuated Measles morbillivirus and Mumps orthorubulavirus and Rubella virus antigens (procedure)	571591000119106
First measles mumps and rubella vaccination given by other healthcare provider (finding)	1037251000000102

We included clinical codes relating to administration of mono-components of the MMR vaccination. After removal of duplicate data entries and merging to the study cohort, 533 children had a clinical code for measles vaccination, and two for mumps vaccination, as opposed to a combined MMR vaccination.

The two datafiles were merged (table S2). 531,469 children with an MMR procedure were not matched to the study denominator because they were not eligible for a timely MMR vaccination in the 19 months before or after 23rd March 2020.

Table S2 – Proportion of children within each cohort who had a MMR procedure recorded in their electronic primary care record

	MMR procedure	No MMR procedure	Total
Pre-pandemic (%)	28,173 (84.8)	5,053 (15.2)	33,226 (100)
Pandemic (%)	25,454 (78.5)	6,992 (21.5)	32,446 (100)

For peer review only

Table S3 – Ethnic background distribution by Clinical Commissioning Group

	White (%)	Mixed and Other (%)	South Asian (%)	Black (%)	Missing (%)	Total (%)
**Barking & Dagenham**	2,136 (27.6)	567 (7.3)	1,164 (15.1)	772 (10.0)	3,096 (40.0)	7,735 (100)
**City & Hackney**	2,713 (28.9)	1,942 (20.7)	162 (1.7)	576 (6.1)	4,009 (42.6)	9,402 (100)
**Havering**	3,171 (43.2)	500 (6.8)	484 (6.6)	353 (4.8)	2,833 (38.6)	7,341 (100)
**Newham**	2,698 (21.3)	1,656 (13.1)	3,572 (28.2)	1,083 (8.6)	3,659 (28.9)	12,668 (100)
**Redbridge**	2,595 (26.6)	1,032 (10.6)	2,821 (28.9)	404 (4.1)	2,912 (29.8)	9,764 (100)
**Tower Hamlets**	1,223 (13.3)	585 (6.4)	2,419 (26.3)	261 (2.8)	4,715 (51.2)	9,203 (100)
**Waltham Forest**	3,981 (41.7)	1,297 (13.6)	1,061 (11.1)	597 (6.3)	2,623 (27.4)	9,559 (100)
**Total**	18,517 (28.2)	7,579 (11.5)	11,683 (17.8)	4,046 (6.2)	23,847 (36.3)	65,672 (100)

Table S4 – Index of Multiple Deprivation quintile associated with LSOAs with less than 60% of children receiving timely MMR vaccination in the pre-pandemic and pandemic cohorts

	**LSOAs with <60% timely vaccination in pre-pandemic cohort (*n*=90)**		**LSOAs with <60% timely vaccination in pandemic cohort (*n*=153)**		**All LSOAs (*n*=1,203)**	
	**n**	**%**	**n**	**%**	**n**	**%**
**Most deprived**	30	33.3	63	41.2	401	33.3
**2**	45	50.0	61	39.9	445	37.0
**3**	9	10.0	19	12.4	187	15.5
**4**	2	2.2	6	3.9	107	8.9
**Least deprived**	2	2.2	4	2.6	58	4.8
**Missing**	2	2.2	0	0.0	5	0.4
**Total**	90	100	153	100	1203	100

For peer review only

Table S5 – Unadjusted and adjusted odds of timely MMR vaccination

	Unadjusted		Adjusted[1] (*n*=65572[2])	
	OR[3]	95% CI[4]	OR[3]	95% CI[4]
**Cohort**				
Control (ref.)	1		1	
COVID-19	0.80	0.77,0.83	0.79	0.76,0.82
**Sex**				
Male (ref.)	1		1	
Female	1.07	1.03,1.11	1.07	1.03,1.11
**CCG[5]**				
Barking & Dagenham	0.84	0.78,0.89	0.88	0.82,0.94
City & Hackney	0.60	0.56,0.63	0.67	0.63,0.71
Havering	1.78	1.64,1.92	1.53	1.40,1.66
Newham (ref.)	1		1	
Redbridge	0.82	0.77,0.87	0.69	0.64,0.73
Tower Hamlets	1.52	1.42,1.63	1.52	1.42,1.64
Waltham Forest	1.21	1.13,1.29	1.21	1.14,1.30
**Ethnic background**				
White (ref.)	1		1	
Mixed and Other	0.68	0.64,0.73	0.77	0.72,0.82
South Asian	1.33	1.25,1.41	1.39	1.30,1.48
Black	0.64	0.59,0.69	0.70	0.65,0.76
Missing	0.75	0.72,0.79	0.77	0.74,0.81
**IMD[6] quintile**				
Most deprived (ref.)	1		1	
2	1.03	0.99,1.07	1.03	0.99,1.08
3	1.26	1.18,1.33	1.24	1.16,1.32
4	1.87	1.71,2.06	1.78	1.61,1.98
Least deprived	2.42	2.07,2.82	2.09	1.78,2.46

[1] Model mutual adjusting for cohort, sex, clinical commissioning group, ethnic background and Index of Multiple Deprivation quintile. [2] 98 children with missing Index of Multiple Deprivation quintile and two children with "Other" sex were excluded from the adjusted model. [3] Odds ratio. [4] 95% confidence interval. [5] Clinical Commissioning Group. [6] Index of Multiple Deprivation.

Table S6 – Measles and mumps cases recorded in primary care in the pre-pandemic and pandemic periods by Clinical Commissioning Group

	Pre-pandemic	Pandemic
**Measles**	326	20
**Mumps**	140	34

For peer review only

BMJ Open

Impact of the COVID-19 pandemic on timeliness and equity of measles, mumps and rubella vaccinations in north-east London: a longitudinal study using electronic health records

Journal:	*BMJ Open*
Manuscript ID	bmjopen-2022-066288.R1
Article Type:	Original research
Date Submitted by the Author:	17-Oct-2022
Complete List of Authors:	Firman, Nicola; Queen Mary University of London, Centre for Primary Care and Public Health<br>Marszalek, Milena; Queen Mary University of London, Centre for Primary Care and Public Health<br>Gutierrez, Ana; Queen Mary University of London, Centre for Primary Care and Public Health<br>Homer, Kate; Queen Mary University of London, Centre for Primary Care and Public Health<br>Williams, Crystal; Queen Mary University of London, Centre for Primary Care and Public Health<br>Harper, Gill; Queen Mary University of London, Centre for Primary Care and Public Health<br>Dostal, Isabel; Queen Mary University of London, Centre for Primary Care and Public Health<br>Ahmed, Zaheer; Queen Mary University of London, Centre for Primary Care and Public Health<br>Robson, John; Queen Mary University of London, Centre for Primary Care and Public Health<br>Dezateux, Carol; Queen Mary University of London, Centre for Primary Care and Public Health
**Primary Subject Heading**:	General practice / Family practice
Secondary Subject Heading:	Paediatrics, Public health, Epidemiology, Infectious diseases
Keywords:	COVID-19, Paediatric infectious disease & immunisation < PAEDIATRICS, PRIMARY CARE, Public health < INFECTIOUS DISEASES, PUBLIC HEALTH

SCHOLARONE™
Manuscripts

I, the Submitting Author has the right to grant and does grant on behalf of all authors of the Work (as defined in the below author licence), an exclusive licence and/or a non-exclusive licence for contributions from authors who are: i) UK Crown employees; ii) where BMJ has agreed a CC-BY licence shall apply, and/or iii) in accordance with the terms applicable for US Federal Government officers or employees acting as part of their official duties; on a worldwide, perpetual, irrevocable, royalty-free basis to BMJ Publishing Group Ltd ("BMJ") its licensees and where the relevant Journal is co-owned by BMJ to the co-owners of the Journal, to publish the Work in this journal and any other BMJ products and to exploit all rights, as set out in our licence.

The Submitting Author accepts and understands that any supply made under these terms is made by BMJ to the Submitting Author unless you are acting as an employee on behalf of your employer or a postgraduate student of an affiliated institution which is paying any applicable article publishing charge ("APC") for Open Access articles. Where the Submitting Author wishes to make the Work available on an Open Access basis (and intends to pay the relevant APC), the terms of reuse of such Open Access shall be governed by a Creative Commons licence – details of these licences and which Creative Commons licence will apply to this Work are set out in our licence referred to above.

Other than as permitted in any relevant BMJ Author's Self Archiving Policies, I confirm this Work has not been accepted for publication elsewhere, is not being considered for publication elsewhere and does not duplicate material already published. I confirm all authors consent to publication of this Work and authorise the granting of this licence.

For peer review only

Impact of the COVID-19 pandemic on timeliness and equity of measles, mumps and rubella vaccinations in north-east London: a longitudinal study using electronic health records

Nicola Firman[1] MSc, Health Data Scientist (0000-0001-5213-5044)*

Milena Marszalek[1], Clinical Research Fellow (0000-0001-5825-0609)*

Ana Gutierrez[1], Senior Primary Care Facilitator

Kate Homer[1], Senior Health Data Scientist

Crystal Williams[1] MSc, Senior Data Analyst

Gill Harper[1] PhD, Post-Doctoral Research Fellow in Health Data Science (0000-0002-3492-2076)

Isabel Dostal[1] MSc, Research Assistant

Zaheer Ahmed[1], Informatics Lead

John Robson[1] MD, Clinical Reader in Primary Care Research & Development (0000-0001-6889-0415)

Carol Dezateux[1] MD, Professor of Clinical Epidemiology and Health Data Science (0000-0001-9787-6276)

[1]Centre for Primary Care, Wolfson Institute of Population Health, Barts and the London School of Medicine and Dentistry, Queen Mary University of London, Yvonne Carter Building, 58 Turner Street, London, E1 2AB

*Correspondence to:
Nicola Firman
nicola.firman@qmul.ac.uk
Centre for Primary Care, Wolfson Institute of Population Health, Barts and the London School of Medicine and Dentistry, Queen Mary University of London, Yvonne Carter Building, 58 Turner Street, London, E1 2AB

or

Dr Milena Marszalek
m.marszalek@qmul.ac.uk
Centre for Primary Care, Wolfson Institute of Population Health, Barts and the London School of Medicine and Dentistry, Queen Mary University of London, Yvonne Carter Building, 58 Turner Street, London, E1 2AB

For peer review only

Abstract

Objectives

To quantify the effect of the COVID-19 pandemic on the timeliness of, and geographic and sociodemographic inequalities in, receipt of first Measles Mumps and Rubella (MMR) vaccination.

Design

Longitudinal study using primary care electronic health records.

Setting

285 general practices in north-east London.

Participants

Children born between 23/08/2017 and 22/09/2018 (pre-pandemic cohort) or between 23/03/2019 and 1/05/2020 (pandemic cohort).

Main outcome measure

Receipt of timely MMR vaccination between 12 and 18 months of age.

Methods

We used logistic regression to estimate the odds ratios (OR; 95% confidence intervals [CI]) of receipt of a timely vaccination adjusting for sex, deprivation, ethnic background, and Clinical Commissioning Group. We plotted choropleth maps of the proportion receiving timely vaccinations.

Results

Timely MMR receipt fell by 4.0% (95% CI: 3.4,4.6) from 79.2% (78.8,79.6) to 75.2% (74.7,75.7) in the pre-pandemic (n=33,226; 51.3% boys) and pandemic (n=32,446; 51.4%) cohorts, respectively. After adjustment, timely vaccination was less likely in the pandemic cohort (0.79; 0.76,0.82), children from Black (0.70; 0.65,0.76), Mixed/Other (0.77; 0.72,0.82) or with missing (0.77; 0.74,0.81) ethnic backgrounds, and more likely in girls (1.07; 1.03,1.11) and those from South Asian backgrounds (1.39; 1.30,1.48). Children living in the least deprived areas were more likely to receive a timely MMR (2.09; 1.78,2.46) but there was no interaction between cohorts and deprivation (Wald statistic: 3.44; p-value=0.49). The proportion of neighbourhoods where less than 60% of children received timely vaccination increased from 7.5% to 12.7% during the pandemic.

Conclusions

The COVID-19 pandemic was associated with a significant fall in timely MMR receipt and increased geographic clustering of measles susceptibility in an area of historically low and inequitable MMR coverage. Immediate action is needed to avert measles outbreaks and support primary care to deliver timely and equitable vaccinations.

For peer review only - http://bmjopen.bmj.com/site/about/guidelines.xhtml

Strengths and limitations of this study

- We used routine primary care electronic health records available in near real time for an entire population of children registered with all NHS general practices in one region of London.
- Coding of routine childhood vaccinations by primary care teams in north-east London is enabled by data entry templates with standardised coding enabling high quality recording of childhood vaccinations at the point of care.
- We used robust statistical methods to investigate inequalities in MMR timeliness and the impact of the COVID-19 pandemic.
- Ethnic background was not recorded in the primary care electronic health records of more than one third of children in our study sample.

For peer review only

For peer review only - http://bmjopen.bmj.com/site/about/guidelines.xhtml

Introduction

The COVID-19 pandemic disrupted routine healthcare and services across the United Kingdom (UK), through rising COVID infections as well as the introduction of social distancing measures and lockdowns.(1) The UK Joint Committee on Vaccination and Immunisation emphasised the importance of continued receipt of routine vaccinations throughout periods of lockdown.(2)

In the 12 months to March 2021, an average of 90.3% of children scheduled to receive a first Measles, Mumps and Rubella (MMR) vaccination had been vaccinated by 24 months of age in England. This was approximately 0.3% lower than for the same period to March 2020, with average levels in both years well below the World Health Organization (WHO) coverage target of 95%.(3)

These national averages conceal significant geographic inequity. The most recent annual local authority coverage data demonstrates that in London, MMR coverage fell from 83.6% to 82.4% at 24 months from 2019-2021.(3) These findings indicate significant disruption across routine child vaccination schedules which are worse in regions with historically low uptake,(4) reflecting pre-existing inequalities observed in MMR uptake in the UK(5) resulting in a measles outbreak and loss of measles 'elimination status' in 2019.(6)

Research investigating the impact of the COVID-19 pandemic on routine vaccination schedules found that MMR uptake during April 2020 dropped initially by 42.5% in London in comparison to the same time period during 2019.(7) In addition to reduced vaccination uptake, a recent systematic review has highlighted the consequential impacts on vaccination inequalities during pandemics,(8) identifying four studies which reported that inequalities in routine vaccination coverage worsened during the pandemic compared with pre-pandemic months. A study in Pakistan(9) identified greater reductions in routine vaccination coverage among children whose parents had lower education than children whose parents had received higher educational levels, whilst a Colombian study(10) found reduced vaccination coverage among children living in rural compared with urban areas. Studies taking place in the United States (US) identified widening inequalities in vaccination coverage during the pandemic by race(11) and Medicaid enrolment.(12)

The findings from this systematic review highlight the importance of understanding the impact of the COVID-19 pandemic on MMR in the UK. Further reductions in MMR uptake will increase the risk of future measles outbreaks, particularly in London where a significant proportion of children start school without the full protection offered by MMR vaccination.(13)

Methods currently used to assess vaccine coverage lack information on timeliness, as well as social, ethnic, and geographic inequalities. Because of their retrospective nature, these methods are not actionable. This is important as it has been acknowledged that regional averages conceal geographical clusters of susceptibility in smaller areas which fuel outbreaks.(14) We examined the impact of the COVID-19 pandemic on timeliness of the first MMR vaccination in north-east London. Specifically, we aimed to quantify the impact of COVID-19 on timeliness of the first MMR vaccination and investigate whether inequalities in receipt of timely MMR vaccination and its geographic clustering were amplified during the pandemic. We also aimed to report the number of measles and mumps cases recorded in primary care in north-east London occurring during the pandemic period and equivalent pre-pandemic period.

Methods

Study design and setting

We carried out a longitudinal study using primary care electronic health records (EHRs) from 285 general practices (GP) in seven geographically contiguous North East London (NEL) Clinical Commissioning Groups (CCGs): Barking & Dagenham, City & Hackney, Havering, Newham, Redbridge, Tower Hamlets, and Waltham

For peer review only - http://bmjopen.bmj.com/site/about/guidelines.xhtml

114 Forest. The study protocol can be found in supplementary file 1 and STrengthening the Reporting of OBservational studies in Epidemiology (STROBE) checklist in supplementary file 2.

116 ***Study population***

117 We defined two cohorts of children eligible to receive their first MMR vaccination between 12 and 18 months of age in the 19 months before and after 23rd March 2020 – the date at which the first national lockdown commenced in the UK. The pre-pandemic cohort comprised those born between 23rd August 2017 and 22nd September 2018, and the pandemic cohort those born between 23rd March 2019 and 1st May 2020.

121 ***Data sources***

122 Pseudonymised data were provided from the NEL Discovery Data Service which receives primary care EHR data on a daily basis from all general practices in NEL. Demographic and clinical data were extracted for 1,192,630 children born between September 2001 and October 2021, ever registered with a NEL general practice and including children who may have died or left the area. Data were extracted on 23rd November 2021 and included all clinical events up to 1st November 2021. All data were extracted and managed according to UK National Health Service (NHS) information governance requirements.(15)

128 ***Data processing***

129 We identified 519,465 children with a NEL GP registration at the time of their first birthday (supplementary file 3, figure S1) and retained only those eligible for the pandemic and pre-pandemic cohorts (figure 1).

131 We extracted sociodemographic and geographic data for each child, together with - for each child - all clinical events relating to MMR procedures. Documentation of the processing of MMR events can be found in supplementary file 3 (figure S2 and tables S1 and S2). As we did not have access to date of birth, a proxy date of birth was derived by assigning day of birth to first day of the week using the calendar week and year of birth.

136 ***Outcome of interest***

137 We defined timely MMR vaccination as receipt of the first MMR vaccination between 12 and 18 months of age, which is consistent with NHS England's definition of a timely MMR vaccination for the Quality and Outcomes Framework (QOF).(16) A vaccination considered not timely may have been given too early (before 12 months of age), late (after 18 months of age) or not recorded (never or not yet received).

141 Cases of measles and mumps were identified in primary care EHRs as events with relevant SNOMED clinical codes (see supplementary file 3, table S3). Only the first instance of each diagnosis code was retained.

143 ***Covariates of interest***

144 We merged 2019 Index of Multiple Deprivation (IMD) rank(17) into the datafile using the 2011 Lower Super Output Area (LSOA) - an area with an average population of 1500 people or 650 households - as the linkage field, and categorised IMD rank into the national quintiles from most to least deprived. Ethnic background was categorised using the NHS classification from information recorded in the primary care record. We grouped ethnic background into four mutually exclusive groups: White ('White British', 'White Irish', or 'any other White background'); Black ('Black African', 'Black Caribbean', or 'any other Black background'); South Asian ('Indian', 'Pakistani', 'Bangladeshi' or 'Sri Lankan'); and Mixed/Other ('any other ethnic background', 'mixed ethnicity', 'Chinese' or 'Asian other'). A Missing category was created where ethnicity was not coded in the primary care record.

For peer review only - http://bmjopen.bmj.com/site/about/guidelines.xhtml

Statistical analyses

We explored variation in the proportion of children receiving a timely MMR vaccination by cohort, sex, CCG, ethnic background and IMD quintile, and described the differences in the proportion of children receiving timely MMR vaccination in each cohort by these covariates.

For children with a GP recorded address with an associated LSOA in NEL, we plotted choropleth maps of the proportion of children receiving timely MMR vaccination in each cohort and the change in proportion between the two cohorts by LSOA, to visualise geographical clustering of MMR vaccination timeliness. LSOAs with fewer than ten eligible children in either the pre-pandemic or pandemic cohorts were suppressed.

We conducted binary logistic regression to estimate the adjusted odds (odds ratio [OR] and 95% confidence interval [CI]) of timely MMR vaccination after adjustment for covariates. We tested an interaction between cohort and IMD quintile to assess whether COVID-19 had widened inequalities in timely vaccination. All analyses were conducted using Stata (MP/15.0).

Patient and public involvement

We involved patients and the public in the communication of study results and dissemination within the local community using accepted principles from the UK Standards for Public Involvement.(18) The aim was to raise awareness of the importance of inequalities in timely childhood vaccinations. We established a patient advisory group, comprising six parents, to co-produce dissemination materials. The PPI group reflected on vaccination inequalities, the study design and how results were delivered. Participants expressed reservations about the categorisation of ethnic background and whether more granular categories could be used in future research. They discussed communication and visualisation of results. Dissemination of results is ongoing and informed by advice about accessing hard-to-reach and existing community groups.

Results

The pre-pandemic and pandemic cohorts comprised 33,226 (51.3% boys) and 32,446 (51.4% boys) children respectively (figure 1). The cohorts were similar with respect to demographic characteristics: the majority lived in the most deprived areas and were from diverse ethnic backgrounds (table 1 and supplementary file 3, table S4). Timely MMR receipt was 4.0% (95% CI: 3.4,4.6) lower in the pandemic compared with the pre-pandemic cohort.

For peer review only

For peer review only - http://bmjopen.bmj.com/site/about/guidelines.xhtml

Table 1: Sample characteristics

	Pre-pandemic cohort (*n*=33,226)			Pandemic cohort (*n*=32,446)			All (*n*=65,672)		
	n	**%**	**95% CI[1]**	***n***	**%**	**95% CI[1]**	***n***	**%**	**95% CI[1]**
**Sex**									
Male	17055	51.3	50.8,51.9	16665	51.4	50.8,49.2	33720	51.4	51.0,51.7
Female	16169	48.7	48.1,49.2	15781	48.6	48.1,49.2	31950	48.6	48.3,49.0
Other	2			0			2		
**CCG[2]**									
Barking & Dagenham	3916	11.8	11.4,12.1	3819	11.8	11.4,12.1	7735	11.8	11.5,12.0
City & Hackney	4771	14.4	14.0,14.7	4631	14.3	13.9,14.7	9402	14.3	14.1,14.6
Havering	3684	11.1	10.8,11.4	3657	11.3	10.9,11.6	7341	11.2	10.9,11.4
Newham	6458	19.4	19.0,19.9	6210	19.1	18.7,19.6	12668	19.3	19.0,19.6
Redbridge	4971	15.0	14.6,15.3	4793	14.8	14.4,15.2	9764	14.9	14.6,15.1
Tower Hamlets	4605	13.9	13.5,14.2	4598	14.2	13.8,14.6	9203	14.0	13.8,14.3
Waltham Forest	4821	14.5	14.1,14.9	4738	14.6	14.2,15.0	9559	14.6	14.3,14.8
**Ethnic background**									
White	9579	28.8	28.3,29.3	8938	27.6	27.1,28.0	18517	28.2	27.9,28.5
Mixed and Other	3813	11.5	11.1,11.8	3766	11.6	11.3,12.0	7579	11.5	11.3,11.8
South Asian	5881	17.7	17.3,18.1	5802	17.9	17.5,18.3	11683	17.8	17.5,18.1
Black	2054	6.2	5.9,6.4	1992	6.1	5.9,6.4	4046	6.2	6.0,6.3
Missing	11899	35.8	35.2,36.3	11948	36.8	36.3,37.4	23847	36.3	35.9,36.7
**IMD[3] quintile**									
Most deprived	12436	37.4	36.9,38.0	11995	37.0	36.4,37.5	24431	37.2	36.8,37.6
2	13464	40.5	40.0,41.1	13306	41.0	40.5,41.5	26770	40.8	40.4,41.1
3	4533	13.6	13.3,14.0	4400	13.6	13.2,13.9	8933	13.6	13.3,13.9
4	1883	5.7	5.4,5.9	1956	6.0	5.8,6.3	3839	5.9	5.7,6.0
Least deprived	847	2.6	2.4,2.7	754	2.3	2.2,2.5	1601	2.4	2.3,2.6
Missing	63	0.2	0.1,0.2	35	0.1	0.1,0.2	98	0.2	0.1,0.2
**Timely MMR vaccination[4]**									
Yes	26315	79.2	78.8,79.6	24402	75.2	74.7,75.7	50717	77.2	76.9,77.5
No	6911	20.8	20.4,21.2	8044	24.8	24.3,25.3	14955	22.8	22.5,23.1
Early	*180*	*0.5*	*0.5,0.6*	*120*	*0.4*	*0.3,0.4*	*300*	*0.5*	*0.4,0.5*
Late	*1678*	*5.1*	*4.8,5.3*	*932*	*2.9*	*2.7,3.1*	*2610*	*4.0*	*3.8,4.1*
Not yet received	*5053*	*15.2*	*14.8,15.6*	*6992*	*21.5*	*21.1,22.0*	*12045*	*18.3*	*18.0,18.6*

[1] 95% confidence interval. [2] Clinical Commissioning Group. [3] Index of Multiple Deprivation. [4] Receipt of first MMR vaccination between 12 and 18 months of age. Not timely is further broken down into three groups: early (before age 12 months), late (after age 18 months) and not yet received.

Children from White, Mixed/Other, and Black ethnic backgrounds had the lowest - and children from South Asian ethnic backgrounds the highest - percentage timely MMR receipt (table 2). There was a strong positive gradient in vaccination timeliness by IMD quintile: relative to those living in the least deprived quintile, the proportion of children receiving a timely MMR vaccination was 10.8% (8.6,13.0) and 14.3% lower (11.8,16.8) in the pre-pandemic and pandemic cohorts respectively.

For peer review only

Table 2: Proportion of children with timely MMR vaccination[1] in each cohort, and the percentage point difference between pre-pandemic and pandemic cohorts, by sociodemographic characteristics

	Pre-pandemic cohort (*n*=26,315)			Pandemic cohort (*n*=24,402)			Percentage point difference[2]	
	n	%	95% CI[3]	*n*	%	95% CI[3]	%	95% CI[3]
**Sex**								
Male	13362	78.3	77.7,79.0	12480	74.9	74.2,75.5	-3.4	-4.3,-2.5
Female	12953	80.1	79.5,80.7	11922	75.5	74.9,76.2	-4.6	-5.5,-3.7
**CCG[4]**								
Barking & Dagenham	2999	76.6	75.2,77.9	2725	71.3	69.9,72.8	-5.3	-7.3,-3.3
City & Hackney	3403	71.3	70.0,72.6	2897	62.6	61.2,63.9	-8.7	-10.6,-6.8
Havering	3205	87.0	85.9,88.0	3095	84.6	83.4,85.8	-2.4	-4.0,-0.8
Newham	5077	78.6	77.6,79.6	4717	76.0	74.9,77.0	-2.6	-4.1,-1.1
Redbridge	3713	74.7	73.5,75.9	3483	72.7	71.4,73.9	-2.0	-3.7,-0.3
Tower Hamlets	3977	86.4	85.3,87.3	3737	81.3	80.1,82.4	-5.1	-6.6,-3.6
Waltham Forest	3941	81.7	80.6,82.8	3748	79.1	77.9,80.2	-2.6	-4.2,-1.0
**Ethnic background**								
White	7865	82.1	81.3,82.9	6874	76.9	76.0,77.8	-5.2	-6.4,-4.0
Mixed and Other	2872	75.3	73.9,76.7	2641	70.1	68.6,71.6	-5.2	-7.2,-3.2
South Asian	4966	84.4	83.5,85.3	4824	83.1	82.2,84.1	-1.3	-2.6,0.0
Black	1517	73.9	71.9,75.7	1374	69.0	66.9,71.0	-4.9	-7.7,-2.1
Missing	9095	76.4	75.7,77.2	8689	72.7	71.9,73.5	-3.7	-4.8,-2.6
**IMD[5] quintile**								
Most deprived	9710	78.1	77.3,78.8	8779	73.2	72.4,74.0	-4.9	-6.0,-3.8
2	10523	78.2	77.5,78.8	9865	74.1	73.4,74.9	-4.1	-5.1,-3.1
3	3675	81.1	79.9,82.2	3437	78.1	76.9,79.3	-3.0	-4.7,-1.3
4	1628	86.5	84.8,87.9	1649	84.3	82.6,85.9	-2.2	-4.4,0.0
Least deprived	753	88.9	86.6,90.8	660	87.5	85.0,89.7	-1.4	-4.6,1.8
Missing	26	41.3	29.9,53.7	12	32.3	20.6,51.2	-9.0	-28.7,10.7

[1] Receipt of MMR vaccination between 12 and 18 months of age. [2] Proportion receiving timely MMR vaccination in pandemic cohort minus the proportion receiving timely MMR vaccination in the pre-pandemic cohort. [3] 95% confidence interval. [4] Clinical Commissioning Group. [5] Index of Multiple Deprivation.

The proportion of LSOAs where fewer than 60% of children received a timely MMR vaccination increased from 7.5% (90) to 12.7% (153) in the pandemic cohort (figures 2a and 2b). These were clustered in parts of City & Hackney, Newham, Redbridge, and Barking and Dagenham. Almost half of LSOAs where fewer than 60% of children received timely MMR vaccinations were assigned to the most deprived IMD quintile compared to one third in the pre-pandemic cohort (supplementary file 3, table S5). The proportion of children receiving a timely MMR vaccination fell during the pandemic period in 634 (52.7%) out of 1203 LSOAs (figure 3), and these were predominantly located in Tower Hamlets and City & Hackney. The proportion increased in 367 LSOAs (30.5%) and remained the same in 13 (1.1%).

After adjustment, timely MMR receipt was less likely in the pandemic cohort (0.79; 0.76,0.82), children from Black (0.70; 0.65,0.76), Mixed/Other (0.77; 0.72,0.82) or with Missing (0.77; 0.74,0.81) ethnic backgrounds, and more likely in girls (1.07; 1.03,1.11) and those from South Asian backgrounds (1.39; 1.30,1.48). Children living in the least deprived areas were more likely to receive a timely MMR (2.09; 1.78,2.46; Wald test statistic: 201.66; *p*-value<0.0001; figure 4 and supplementary file 3, table S6), but there was no interaction between

For peer review only

For peer review only - http://bmjopen.bmj.com/site/about/guidelines.xhtml

cohorts and deprivation (Wald statistic: 3.44; *p*-value=0.49). Relative to children registered with a general practice in Newham, timely MMR receipt was less likely among children in Barking & Dagenham (0.88; 0.82,0.94), City & Hackney (0.67; 0.63,0.71) or Redbridge (0.69; 0.64,0.73) and more likely among those registered to a general practice in Havering (1.53; 1.40,1.66), Tower Hamlets (1.52; 1.42,1.64) and Waltham Forest (1.21; 1.14,1.30).

In NEL, there were 20 measles and 34 mumps cases recorded in primary care during the pandemic period, compared to 325 and 140, respectively, in the equivalent pre-pandemic period (supplementary file 3, table S7).

Discussion

Summary of key findings

In the period preceding the COVID-19 pandemic in NEL, only 79% of children received their first MMR vaccination on time; this proportion fell by an average of 4% during the pandemic. The gap between the most and least deprived areas increased by 3.5% during the pandemic period. While this relative inequality did not appear to worsen during the pandemic, these average figures conceal marked inequity at a lower geographical level: the percentage of LSOAs, where fewer than 60% of children received their MMR on time increased from 7.5% to 12.7% in the pandemic, particularly in the most deprived LSOAs. Hence delayed receipt of MMR is geographically clustered in more deprived neighbourhoods, and this has worsened during the pandemic. These findings are in an area of London where no CCG has met the WHO MMR target of 95% coverage.(3) In the absence of national data, our analyses show for the first time how far this region of London is from achieving the new Quality and Outcomes Framework target for MMR timeliness of 90-95%.(16)

Strengths and limitations

We used routine primary care EHRs available in near real time for an entire population of children registered with all NHS general practices in one region of London. We have been able to demonstrate – in a geographically contiguous area – neighbourhoods with very high proportions of children who are not immunised promptly. These results further highlight the inequalities in vaccination timeliness and infection risks experienced by poorer children, their families and communities.

Coding of routine childhood vaccinations by primary care teams in NEL is enabled by data entry templates with standardised coding enabling high quality recording of childhood vaccinations at the point of care. We used robust statistical methods to investigate inequalities in MMR timeliness and the impact of the COVID pandemic.

Limitations include missing ethnic codes in 36% of the cohort. Our analyses suggest that those with missing ethnicity were less likely to receive a timely MMR vaccine during the pandemic and highlight the importance of improving routine recording of ethnicity in primary care. Whilst our study has focused on timeliness of the first MMR vaccination, it is important to recognise that two doses of MMR are essential for full protection.(19) Additional research investigating timeliness of the second MMR vaccination would further our understanding and improve identification of children with increased measles susceptibility.

Comparison with existing literature

Our findings align with trends indicating a global decrease in uptake of MMR vaccination, both in developing and developed countries.(10, 20, 21) Some studies reported a decline in uptake of more than 50% during the height of the first wave of the pandemic.(22-24) Globally, measles-containing vaccine coverage estimates were 7.9% lower than expected had there been no COVID-19 pandemic, affecting an estimated 8.9 million children.(25) In England, initial reductions in the number of children receiving their first MMR were followed by a short period of recovery, compared to the same period in 2019, despite continued physical distancing

For peer review only - http://bmjopen.bmj.com/site/about/guidelines.xhtml

measures remaining in place.(26) However this increase was short-lived, and the weekly count of children receiving their MMR vaccination in 2020 remained consistently lower or the same as in 2019 in for the rest of 2020. Our findings may be explained in part by evidence from qualitative research studies demonstrating that a transition to remote consultations during the pandemic caused some confusion for parents around attending services for routine vaccination.(4) Internationally, fear of COVID-19 exposure in healthcare settings was also found to play a large role in decreased vaccination uptake,(25, 27, 28) despite evidence that the risk to benefit ratio was in favour of continuing vaccination delivery during the pandemic.(29)

The link between childhood vaccination inequalities and ethnicity has been explored in other studies, demonstrating evidence of reduced timeliness in certain ethnic groups.(30) However, there is heterogeneity within these results according to geographical area of interest.(31) We found that children from Black and Mixed/Other ethnic backgrounds were less likely to receive timely vaccination, broadly consistent with findings from studies of COVID-19 vaccination uptake which have shown lowest uptake among people from Black ethnic backgrounds.(32, 33) Additional qualitative evidence suggest women from minority ethnic backgrounds were more likely to find it difficult to access and felt less safe accessing vaccinations for their babies during the COVID-19 pandemic.(34)

Relationships between socioeconomic deprivation and vaccination timeliness appear to be more consistent. A study in Scotland examining vaccination inequity and timeliness demonstrates that the most deprived decile experienced a nearly 50% increased risk of delayed vaccination relative to the least deprived decile for both doses of MMR in the years leading up to the pandemic.(35) Despite this, and in contrast to England, vaccination uptake rose significantly across the first lockdown period in Scotland, with 7,000 more children receiving timely routine vaccinations compared with the previous year.(36) The authors of this study speculate that greater flexibility in working patterns offered to many parents during the lockdown period may have increased the accessibility of vaccination appointments.

Our findings are consistent with existing evidence based on Cover of Vaccination Evaluated Rapidly (COVER) programme data which confirm that London has a longstanding and disproportionately lower MMR uptake relative to the rest of the UK.(3, 37) There is recognised variation between different CCGs in NEL, with lowest uptake in City & Hackney. While particular cultural beliefs held by the Charedi Jewish population in City & Hackney are known to influence uptake of the MMR vaccination,(38) recent evidence suggests that difficulties in accessing vaccination services are also an important factor in this community.(39) The factors responsible for differences between the other CCGs merit further investigation.

Mapping measles vaccinations and outbreaks geospatially enables more granular identification of neighbourhoods requiring focussed interventions.(40) Our choropleth maps demonstrate clustering of delayed MMR vaccination in more deprived neighbourhoods – these findings align with previous studies mapping measles outbreak susceptibility(5) and underscore the importance of actionable real-time information on vaccine timeliness to avert further outbreaks of measles. Our geographic analyses identified an increase in the proportion of children receiving a timely MMR vaccination during the pandemic period in almost one third of NEL LSOAs. This finding may reflect the innovative measures implemented in some London general practices throughout the pandemic, including vaccinating outside of practice buildings and drive-through services, which may have made routine vaccination more accessible for families.(41)

Our study did not identify an increase in inequity during the pandemic by an area-level measure of deprivation. This is in contrast to findings from Michigan, US, where the difference in proportion of Medicaid-enrolled children with up-to-date vaccination coverage compared with children not enrolled in Medicaid with up-to-date vaccination coverage increased during the pandemic.(12) This difference is likely to reflect differences in UK and US healthcare systems, as well as the use of an area-level indicator of derivation compared with an individual-level indicator in an area of London with high levels of area level deprivation.

Implications for research, policy and practice

For peer review only - http://bmjopen.bmj.com/site/about/guidelines.xhtml

Gaps in MMR vaccination coverage increase measles-susceptibility, and in 2019, there were 800,000 confirmed cases of measles globally.(42) Measles outbreaks have occurred in 2021 in at least half of the 26 countries that suspended their measles vaccination programs.(29, 42, 43) There is evidence that the introduction of social distancing measures, school closures and travel restrictions reduced exposure to vaccine-preventable childhood infections.(44) In an analysis of English hospital admissions there was a 90% and a 53% reduction in hospital admissions for measles and mumps, respectively, among children aged 0-14 years in the pandemic period compared to the preceding three year average, although this study was unable to examine infections managed in primary care.(44) In NEL, we identified 20 measles and 34 mumps cases in primary care during the pandemic period, compared to 325 and 140, respectively, in the equivalent pre-pandemic period.

With a reduction in infection and exposure to infection, measles vaccination may receive less priority in a health care system already facing multiple challenges.(45) Awareness and retention of existing WHO targets is critical to prevent measles outbreaks, especially given that measles is the most infectious virus, with a reproduction number of 12–18.(46) The need for targeted public health interventions around routine childhood vaccinations in the context of the pandemic has been recognised internationally,(47, 48) as well as in England where a recent campaign by NHS England is encouraging parents of 740,000 children who are not fully vaccinated against MMR to make appointments with their GP practice.(49) There is strong evidence to support the effectiveness of primary care led quality improvement programmes to improve vaccine uptake.(37) National measures to tackle these inequalities include NHS England's Quality and Outcomes Frameworks to incentivise timely routine childhood vaccinations in primary care.(50) In London a primary care led quality improvement programme has been launched to tackle inequalities in timeliness of routine preschool childhood vaccinations.(51)

Conclusion

Routine vaccination schedules have been disrupted during the COVID-19 pandemic. Our study adds important new evidence of the impact on timeliness of MMR vaccinations, and demonstrates unwarranted variation by neighbourhood, ethnicity and deprivation. This data provides further evidence to prioritise quality improvement and catch-up campaigns to achieve herd immunity and prevent measles outbreaks. It provides actionable information in populations and geographies experiencing significant health inequalities.

** ** **

Acknowledgements

We are grateful to colleagues within the Clinical Effectiveness Group for access to and expertise in using general practice data. We are also grateful to Professor Bianca de Stavola (Great Ormond Street Institute of Child Health) for her statistical guidance and to Professor Helen Bedford (Great Ormond Street Institute of Child Health) for her clinical expertise and guidance. This work uses data provided by patients and collected by the NHS as part of their care and support. We would also like to acknowledge our PPI participants for their invaluable insights into patient perspectives around dissemination of our research to the wider community.

Contributors

CD and JR obtained funding for the study. NF and CD conceptualised and designed the analyses. AG, ID and ZA provided guidance for specifying clinical codes. CW and KH extracted data. MM carried out the literature search. NF conducted the analyses, generated tables and figures and drafted the initial manuscript. GH produced the choropleth maps. All authors contributed to the interpretation of analyses and reviewed and revised the manuscript. All authors were involved in writing the paper and had final approval of the submitted and published manuscript. The corresponding authors attest that all listed authors meet authorship criteria and that no others meeting the criteria have been omitted. CD is the guarantor and accepts full responsibility for the conduct of the study, had access to the data, and controlled the decision to publish.

For peer review only

Funding

This research was funded by a grant from Barts Charity ref: MGU0419. This work was supported by Health Data Research UK, which is funded by the UK Medical Research Council, Engineering and Physical Sciences Research Council, Economic and Social Research Council, Department of Health and Social Care (England), Chief Scientist Office of the Scottish Government Health and Social Care Directorates, Health and Social Care Research and Development Division (Welsh Government), Public Health Agency (Northern Ireland), British Heart Foundation and Wellcome. MM is supported by a locally funded National Institute for Health Research Academic Clinical Fellowship. The funders had no role in study design, data collection and analysis, decision to publish, or preparation of the manuscript.

Competing interests

All authors have completed the ICMJE uniform disclosure form at http://www.icmje.org/disclosure-of-interest/ and declare: no support from any organisation for the submitted work; no financial relationships with any organisations that might have an interest in the submitted work in the previous three years; no other relationships or activities that could appear to have influenced the submitted work.

Ethics approval

This study was approved by the Discovery board for service evaluation (measuring what standard of care this service achieved) and analysed routinely acquired de-identified data: hence no research ethics committee approval was required by the Health Research Authority. Access to general practice data is enabled by data sharing agreements between the Discovery Data Service and general practice data controllers. The Discovery Programme Board has approved data access by the REAL Child Health programme.

Data availability statement

Access to general practice data is enabled by data sharing agreements between the Discovery Data Service and general practice data controllers. The Discovery Programme Board has approved data access by the REAL Child Health programme team for research on the condition that it is not onwardly shared.

Transparency statement

The corresponding author affirms that the manuscript is an honest, accurate, and transparent account of the study being reported; that no important aspects of the study have been omitted; and that any discrepancies from the study as planned (and, if relevant, registered) have been explained.

Copyright/license for publication

The corresponding author has the right to grant on behalf of all authors and does grant on behalf of all authors, a worldwide licence to the Publishers and its licensees in perpetuity, in all forms, formats and media (whether known now or created in the future), to i) publish, reproduce, distribute, display and store the Contribution, ii) translate the Contribution into other languages, create adaptations, reprints, include within collections and create summaries, extracts and/or, abstracts of the Contribution, iii) create any other derivative work(s) based on the Contribution, iv) to exploit all subsidiary rights in the Contribution, v) the inclusion of electronic links from the Contribution to third party material where-ever it may be located; and, vi) licence any third party to do any or all of the above.

References

For peer review only - http://bmjopen.bmj.com/site/about/guidelines.xhtml

1. Saxena S, Skirrow H, Bedford H. Routine vaccination during covid-19 pandemic response. Bmj. 2020;369:m2392.
2. Public Health England. Impact of physical distancing measures due to COVID-19 pandemic in England on childhood vaccination counts up to week 41, and vaccine coverage up to August 2020. 2020. Contract No.: 20.
3. NHS Digital. Childhood Vaccination Coverage Statistics - 2020-21 2022 [Available from: https://digital.nhs.uk/data-and-information/publications/statistical/nhs-immunisation-statistics/england---2020-21.
4. Bell S, Clarke R, Paterson P, Mounier-Jack S. Parents' and guardians' views and experiences of accessing routine childhood vaccinations during the coronavirus (COVID-19) pandemic: A mixed methods study in England. PLOS ONE. 2021;15(12):e0244049.
5. Keenan A, Ghebrehewet S, Vivancos R, Seddon D, MacPherson P, Hungerford D. Measles outbreaks in the UK, is it when and where, rather than if? A database cohort study of childhood population susceptibility in Liverpool, UK. BMJ Open. 2017;7(3):e014106.
6. National Audit Office. Investigation into pre-school vaccinations: National Audit Office; 2019 [Available from: https://www.nao.org.uk/report/investigation-into-pre-school-vaccinations/.
7. McDonald HI, Tessier E, White JM, Woodruff M, Knowles C, Bates C, et al. Early impact of the coronavirus disease (COVID-19) pandemic and physical distancing measures on routine childhood vaccinations in England, January to April 2020. Euro Surveill. 2020;25(19).
8. Spencer N, Markham W, Johnson S, Arpin E, Nathawad R, Gunnlaugsson G, et al. The Impact of COVID-19 Pandemic on Inequity in Routine Childhood Vaccination Coverage: A Systematic Review. Vaccines (Basel). 2022;10(7).
9. Chandir S, Siddiqi DA, Setayesh H, Khan AJ. Impact of COVID-19 lockdown on routine immunisation in Karachi, Pakistan. The Lancet Global Health. 2020;8(9):e1118-e20.
10. Moreno-Montoya J, Ballesteros SM, Rojas Sotelo JC, Bocanegra Cervera CL, Barrera-López P, De la Hoz-Valle JA. Impact of the COVID-19 pandemic on routine childhood immunisation in Colombia. Arch Dis Child. 2022;107(3):e4.
11. Ackerson BK, Sy LS, Glenn SC, Qian L, Park CH, Riewerts RJ, et al. Pediatric Vaccination During the COVID-19 Pandemic. Pediatrics. 2021;148(1).
12. Bramer CA, Kimmins LM, Swanson R, Kuo J, Vranesich P, Jacques-Carroll LA, et al. Decline in child vaccination coverage during the COVID-19 pandemic - Michigan Care Improvement Registry, May 2016-May 2020. Am J Transplant. 2020;20(7):1930-1.
13. Tiley KS, White JM, Andrews N, Ramsay M, Edelstein M. Inequalities in childhood vaccination timing and completion in London. Vaccine. 2018;36(45):6726-35.
14. O'Leary ST, Trefren L, Roth H, Moss A, Severson R, Kempe A. Number of Childhood and Adolescent Vaccinations Administered Before and After the COVID-19 Outbreak in Colorado. JAMA Pediatrics. 2020:305–7.
15. NHS England. About information governance: NHS; 2022 [Available from: https://www.england.nhs.uk/ig/about/.
16. British Medical Association, National Health Service. Quality and Outcomes Framework guidance for 2021/22 2021 [Available from: https://www.england.nhs.uk/wp-content/uploads/2021/03/B0456-update-on-quality-outcomes-framework-changes-for-21-22-.pdf.
17. Ministry of Housing Communities & Local Government. The English Indices of Deprivation 2019 - Frequently Asked Questions (FAQs) 2016 [Available from: https://assets.publishing.service.gov.uk/government/uploads/system/uploads/attachment_data/file/853811/IoD2019_FAQ_v4.pdf.
18. NIHR Centre for Engagement and Dissemination. UK Standards for Public Involvement 2022 [Available from: https://sites.google.com/nihr.ac.uk/pi-standards/standards?authuser=0.
19. Public Health England. Measles: the green book, chapter 21. In: Public Health England, editor. The Green Book2019.

For peer review only - http://bmjopen.bmj.com/site/about/guidelines.xhtml

20. Jain R, Chopra A, Falézan C, Patel M, Dupas P. COVID-19 related immunization disruptions in Rajasthan, India: A retrospective observational study. Vaccine. 2021;39(31):4343-50.
21. Aizawa Y, Katsuta T, Sakiyama H, Tanaka-Taya K, Moriuchi H, Saitoh A. Changes in childhood vaccination during the coronavirus disease 2019 pandemic in Japan. Vaccine. 2021;39(29):4006-12.
22. Silveira MF, Tonial CT, Goretti KMA, Teixeira AMS, Hallal PC, Maria BMA, et al. Missed childhood immunizations during the COVID-19 pandemic in Brazil: Analyses of routine statistics and of a national household survey. Vaccine. 2021;39(25):3404-9.
23. Shen AK, Bramer CA, Kimmins LM, Swanson R, Vranesich P, Orenstein W. Vaccine Coverage Across the Life Course in Michigan During the COVID-19 Pandemic: January–September 2020. Am J Public Health. 2021;111(11):2027-35.
24. Bhadoria SS, Shukla DK, Joshi A. COVID-19 Pandemic: A Speed Breaker for Routine Immunisation. Journal of Clinical and Diagnostic Research. 2021;15(4):LC32-LC5.
25. Causey K, Fullman N, Sorensen RJD, Galles NC, Zheng P, Aravkin A, et al. Estimating global and regional disruptions to routine childhood vaccine coverage during the COVID-19 pandemic in 2020: a modelling study. Lancet. 2021;398(10299):522-34.
26. Public Health England. Impact of COVID-19 on childhood vaccination counts to week 4 in 2021, and vaccine coverage to Decemeber 2020 in England: interim analyses. Public Health England; 2021. Contract No.: 3.
27. Piché-Renaud PP, Ji C, Farrar DS, Friedman JN, Science M, Kitai I, et al. Impact of the COVID-19 pandemic on the provision of routine childhood immunizations in Ontario, Canada. Vaccine. 2021;39(31):4373-82.
28. Huss G, Magendie C, Pettoello-Mantovani M, Jaeger-Roman E. Implications of the COVID-19 Pandemic for Pediatric Primary Care Practice in Europe. The Journal of Pediatrics. 2021;233:290-1.e2.
29. Shet A, Dhaliwal B, Banerjee P, DeLuca A, Carr K, Britto C, et al. Childhood immunisations in India during the COVID-19 pandemic. BMJ Paediatrics Open. 2021;5(1):e001061.
30. Bielecki K, Kirolos A, Willocks LJ, Pollock KG, Gorman DR. Low uptake of nasal influenza vaccine in Polish and other ethnic minority children in Edinburgh, Scotland. Vaccine. 2019;37(5):693-7.
31. Baker D, Garrow A, Shiels C. Inequalities in immunisation and breast feeding in an ethnically diverse urban area: cross-sectional study in Manchester, UK. J Epidemiol Community Health. 2011;65(4):346-52.
32. Gaughan CH, Razieh C, Khunti K, Banerjee A, Chudasama YV, Davies MJ, et al. COVID-19 vaccination uptake amongst ethnic minority communities in England: a linked study exploring the drivers of differential vaccination rates. Journal of Public Health. 2022;fdab400.
33. Hoang U, de Lusignan S, Joy M, Sherlock J, Williams J, Bankhead C, et al. National rates and disparities in childhood vaccination and vaccine-preventable disease during the COVID-19 pandemic: English sentinel network retrospective database study. Arch Dis Child. 2022.
34. Skirrow H, Barnett S, Bell S, Mounier-Jack S, Kampmann B, Holder B. Women's views and experiences of accessing pertussis vaccination in pregnancy and infant vaccinations during the COVID-19 pandemic: A multi-methods study in the UK. Vaccine. 2022;40(34):4942-54.
35. Haider EA, Willocks LJ, Anderson N. Identifying inequalities in childhood immunisation uptake and timeliness in southeast Scotland, 2008-2018: A retrospective cohort study. Vaccine. 2019;37(37):5614-24.
36. McQuaid F, Mulholland R, Sangpang Rai Y, Agrawal U, Bedford H, Cameron JC, et al. Uptake of infant and preschool immunisations in Scotland and England during the COVID-19 pandemic: An observational study of routinely collected data. PLOS Medicine. 2022;19(2):e1003916.
37. Cockman P, Dawson L, Mathur R, Hull S. Improving MMR vaccination rates: herd immunity is a realistic goal. BMJ. 2011;343:d5703.
38. Henderson L, Millett C, Thorogood N. Perceptions of childhood immunization in a minority community: qualitative study. Journal of the Royal Society of Medicine. 2008;101(5):244-51.

For peer review only - http://bmjopen.bmj.com/site/about/guidelines.xhtml

39. Letley L, Rew V, Ahmed R, Habersaat KB, Paterson P, Chantler T, et al. Tailoring immunisation programmes: Using behavioural insights to identify barriers and enablers to childhood immunisations in a Jewish community in London, UK. Vaccine. 2018;36(31):4687-92.
40. Durrheim DN, Andrus JK, Tabassum S, Bashour H, Githanga D, Pfaff G. A dangerous measles future looms beyond the COVID-19 pandemic. Nat Med. 2021;27(3):360-1.
41. Skirrow H, Flynn C, Heller A, Heffernan C, Mounier-Jack S, Chantler T. Delivering routine immunisations in London during the COVID-19 pandemic: lessons for future vaccine delivery. A mixed-methods study. BJGP Open. 2021;5(4):BJGPO.2021.0021.
42. Feldman AG, O'Leary ST, Danziger-Isakov L. The Risk of Resurgence in Vaccine-Preventable Infections Due to Coronavirus Disease 2019-Related Gaps in Immunization. Clin Infect Dis. 2021;73(10):1920-3.
43. Olusanya OA, Bednarczyk RA, Davis RL, Shaban-Nejad A. Addressing Parental Vaccine Hesitancy and Other Barriers to Childhood/Adolescent Vaccination Uptake During the Coronavirus (COVID-19) Pandemic. Front Immunol. 2021;12:663074.
44. Kadambari S, Goldacre R, Morris E, Goldacre MJ, Pollard AJ. Indirect effects of the covid-19 pandemic on childhood infection in England: population based observational study. BMJ. 2022;376:e067519.
45. Bedford H, Donovan H. We need to increase MMR vaccine uptake urgently. BMJ. 2022;376:o818.
46. Guerra FM, Bolotin S, Lim G, Heffernan J, Deeks SL, Li Y, et al. The basic reproduction number (R0) of measles: a systematic review. The Lancet Infectious Diseases. 2017;17(12):e420-e8.
47. MacDonald NE, Comeau JL, Dube E, Bucci LM. COVID-19 and missed routine immunizations: designing for effective catch-up in Canada. Can J Public Health. 2020;111(4):469-72.
48. World Health Organization. Guiding principles for immunization activities during the COVID-19 pandemic: interim guidance, 26 March 2020 Geneva: World Health Organization; 2020 [updated 2020. Available from: https://apps.who.int/iris/handle/10665/331590.
49. NHS England. News: NHS urges parents to book children in for essential MMR vaccination: NHS; 2022 [Available from: https://www.england.nhs.uk/2022/09/nhs-urges-parents-to-book-children-in-for-essential-mmr-vaccination/.
50. British Medical Association, NHS England. Update to the GP contract agreement 2020/21 - 2023/24 2020 [Available from: https://www.england.nhs.uk/wp-content/uploads/2020/03/update-to-the-gp-contract-agreement-v2-updated.pdf.
51. Clinical Effectiveness Group Queen Mary University of London. Preventing future measles outbreaks: Queen Mary University of London; 2021 [Available from: https://www.qmul.ac.uk/blizard/ceg/about-us/case-studies/preventing-future-measles-outbreaks/.

For peer review only - http://bmjopen.bmj.com/site/about/guidelines.xhtml

Figure 1: Study sample

Figure 2a: Proportion of children receiving timely MMR vaccination in the pre-pandemic cohort, by 2011 Lower Super Output Area[1]

[1] Lower super output area of the child's home address, as recorded in their general practice electronic health record.

Figure 2b: Proportion of children receiving timely MMR vaccination in the pandemic cohort, by 2011 Lower Super Output Area[1]

[1] Lower super output area of the child's home address, as recorded in their general practice electronic health record.

Figure 3: Change[1] in the proportion of children receiving timely MMR vaccination between pre-pandemic and pandemic cohorts, by 2011 Lower Super Output Area[2]

[1] Proportion receiving timely MMR vaccination in pandemic cohort minus the proportion receiving timely MMR vaccination in the pre-pandemic cohort. [2] Lower super output area of the child's home address, as recorded in their general practice electronic health record.

Figure 4: Adjusted log odds of timely MMR vaccination[1]

[1] Model mutual adjusting for cohort, sex, Clinical Commissioning Group, ethnic background and Index of Multiple Deprivation quintile. [2] Odds ratio. [3] 95% confidence interval.

Supplementary file 1 – Study protocol

Supplementary file 2 – STROBE checklist

Supplementary file 3 – Additional tables and figures

For peer review only

For peer review only - http://bmjopen.bmj.com/site/about/guidelines.xhtml

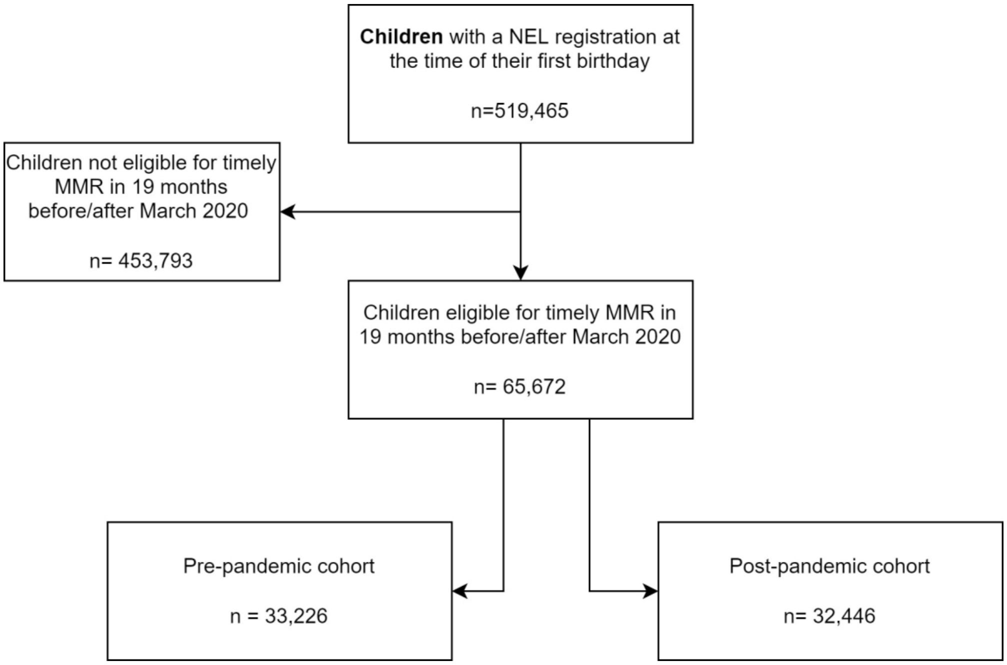

Figure 1 – Study sample

614x403mm (72 x 72 DPI)

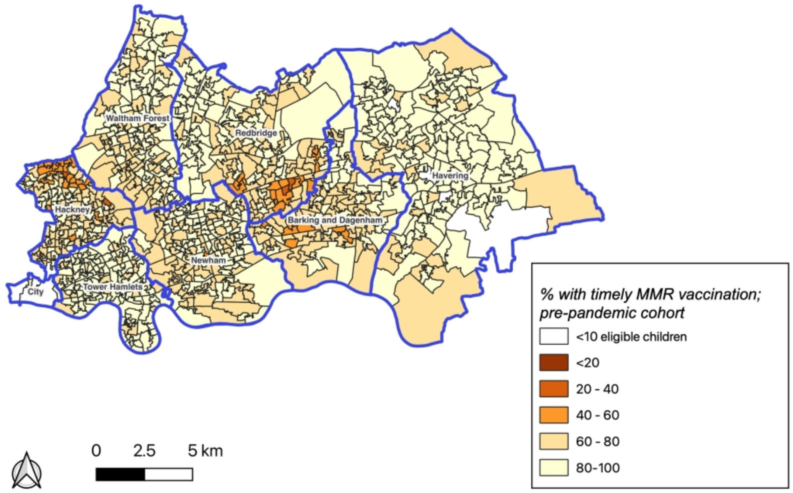

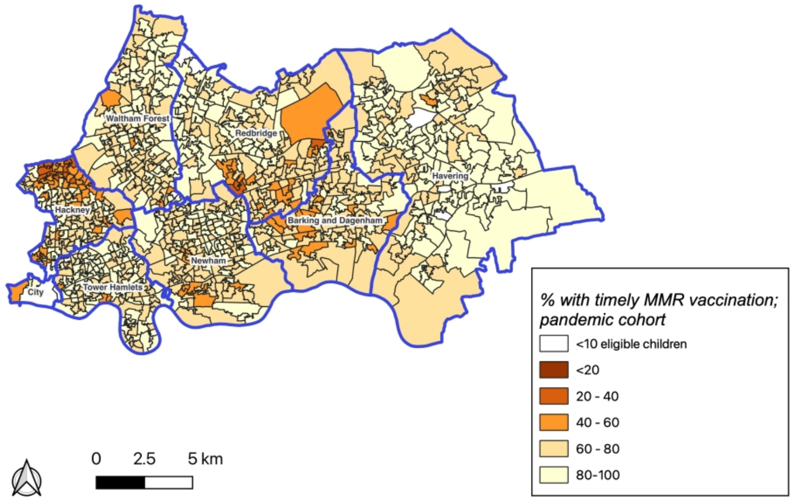

Figure 2a – Proportion of children receiving timely MMR vaccination in the pre-pandemic cohort, by 2011 Lower Super Output Area[1]

Figure 2b – Proportion of children receiving timely MMR vaccination in the pandemic cohort, by 2011 Lower Super Output Area[1]

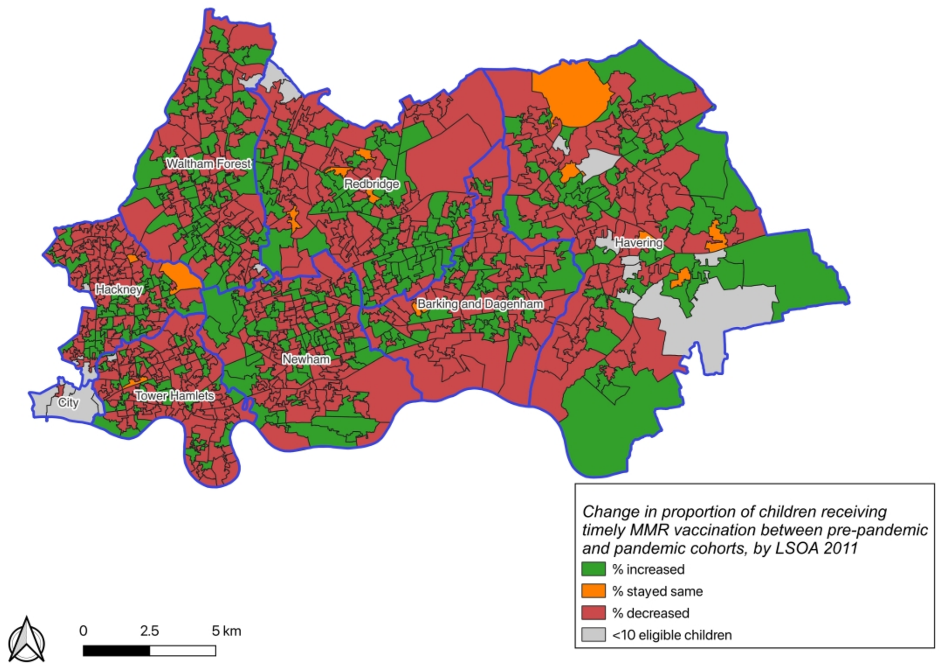

Figure 3 – Change[1] in the proportion of children receiving timely MMR vaccination between pre-pandemic and pandemic cohorts, by 2011 Lower Super Output Area[2]

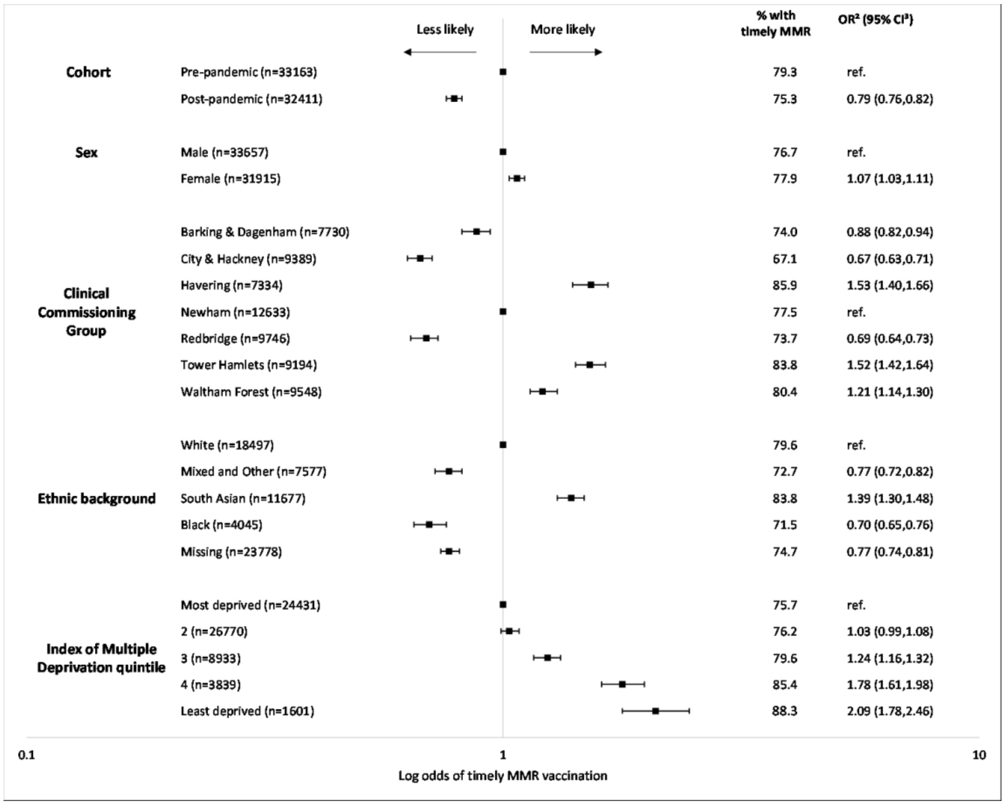

Figure 4 – Adjusted log odds of timely MMR vaccination[1]

318x254mm (118 x 118 DPI)

COVID-19 and childhood MMR immunisation study protocol

Preliminary title: Assessing the impact of the COVID-19 pandemic on timeliness of MMR uptake in east London: cross-sectional analysis of primary care electronic health records

Writing group: Nicola Firman, Milena Marszalek, Ana Gutierrez, Kate Homer, Crystal Williams, Gill Harper, Isabel Dostal, Zaheer Ahmed, John Robson, Carol Dezateux

Background/rationale

The COVID-19 pandemic has disrupted routine health care and services across the UK, through rising COVID infections as well as the introduction of social distancing measures and lockdowns.(1) The United Kingdom (UK) Joint Committee on Vaccination and Immunisation (JCVI) has emphasised the importance of children continuing to receive routine childhood vaccinations according to the national schedule throughout periods of lockdown.(2)

In the UK, childhood vaccine coverage is routinely assessed quarterly using the COVER (Cover of vaccination evaluated rapidly) programme for children who have reached their first, second, or fifth birthdays. Given the timing of data extractions for COVER, the UK Health Security Agency (HAS) has indicated that this system cannot provide reliable information of the impact of the pandemic on vaccine coverage for England. However, evidence suggests that vaccine coverage measured at 24 months among children scheduled to receive Measles, Mumps and Rubella (MMR) vaccination from March 2020 onwards in England was 90.3%, around 0.3% lower than 2019, with average levels in both years well below the WHO coverage target of 95%.(2, 3) This is broadly in line with data from approximately 38% of GP practices in England, which suggests that vaccination counts for MMR vaccine were 2.1% lower by the end of 2020 than for the equivalent period in 2019. Qualitative studies in the UK looking at attitudes towards routine vaccination during the pandemic report parental fear of attending routine vaccination appointments, reduced concern about catching vaccine-preventable diseases during lockdown, and routine appointment re-scheduling. These findings support emerging research that demonstrates disruption across routine child vaccination schedules.(4)

The measures currently used lack information on vaccination timeliness, impacts on existing vaccine coverage inequalities and do not include areas of historic MMR low uptake. This is important as it has been acknowledged that area-level averages conceal geographical clusters of susceptibility which fuel outbreaks.(5) A systematic review found that there is currently no published literature looking at both routine vaccination schedule disruption and consequential impacts on vaccination inequalities during pandemics.(6) More specific literature exists around uptake trends for particular vaccinations - Macdonald et al. demonstrated that MMR uptake during April 2020 dropped by 42·5% in London in comparison to the same time period during 2019.(7) Overall, MMR uptake decreased by 19.8% in England, before recovering over the following months.(8) Most recent local authority coverage data demonstrates that on London, MMR rates have fallen from 83.6% to 82.4% at 24 months from 2019-2021.(9) Areas previously demonstrating lower MMR uptake were worst affected, including London and the Midlands.(7) UK Cover of vaccination evaluated rapidly (COVER) reports look at vaccine coverage at 12, 24 months, and five years, but do not encompass timeliness measures.

These trends reflect pre-existing inequalities observed in MMR uptake in the UK.(10) Historically, the UK lost its measles ‘elimination status’ in 2019 due to consistently low MMR uptake.(11) Further reduction in MMR vaccinations will increase the risk of measles outbreaks, particularly in London where a significant proportion of children start school without the full protection offered by MMR vaccination.(12)

Objectives

This protocol sets out to understand the wider impact of COVID-19 on timeliness of MMR vaccinations in seven east London Clinical Commissioning Groups (CCGs). Specifically:

1. To understand the impact of COVID-19 and periods of lockdown on MMR vaccination timeliness
2. To describe geographical clustering of MMR vaccination timeliness
3. To investigate if COVID-19 has amplified inequalities in receipt of timely MMR vaccination

For peer review only

Methods

Study design

A cross-sectional study using primary care electronic health records (EHRs) from child patients registered at 285 general practices in seven geographically contiguous east London CCGs: Barking & Dagenham, City & Hackney, Havering, Newham, Redbridge, Tower Hamlets, and Waltham Forest.

Study sample

The study population will include all children eligible to receive their first MMR vaccination at age 12 to 18 months old, respectively, in the 19 months before and after 23rd March 2020. All children ever registered at one of the 285 general practices between September 2001 and October 2021 will be included.

Data

Data will be extracted from the North East London (NEL) Discovery Data Service (DDS) which receives primary care electronic health record data on a daily basis from all general practices in NEL, on 23rd November 2021. Events recorded up until 1st November 2021 will be included. All data will be extracted and managed according to UK NHS information governance requirements.

We will analyse person-level data for children born between 22nd August 2017 and 22nd September 2018 (pre-pandemic cohort) and 23rd March 2019 and 1st May 2020 (pandemic cohort). These children would be eligible to receive timely MMR vaccination between 12 and 18 months of age in the 19 months before and after England entered the first lockdown on 23rd March 2020, respectively. For each child, the person-level dataset will contain: a pseudonymised person identifier; calendar week, month and year of birth; ethnic background; sex; 2011 lower super output area (LSOA) of the child's address; CCG; MMR clinical code (Read or SNOMED); and date of MMR vaccination. To avoid disclosure of identifiable data, a proxy date of birth will be derived by assigning the week commencing date using the calendar week and year of birth. Consequently, date of birth could be up to six days earlier than the child's actual date of birth. We will merge 2015 Index of Multiple Deprivation (IMD) score and rank(13) into the datafile using LSOA as the linkage field, and categorise IMD rank into quintiles from most to least deprived.

Following guidance from the Green Book, MMR vaccinations given before a child's first birthday will be ignored.(14) Where available, subsequent MMR records will be treated as the child's first vaccination, otherwise early MMR vaccinations will be retained in the dataset and later considered as "not timely".

The following records will be excluded from analyses:

- Records with an MMR clinical code but no date
- Exact duplicate records
- Duplicate records where the date is the same but MMR clinical code differs
- Latter of multiple MMR events

Statistical methods

We will identify timely MMR vaccination among children aged between 12 and 18 months when they received their MMR vaccination. We will explore variation in the proportion of children receiving timely MMR vaccination by cohort, sex, CCG, ethnic background and IMD quintile, and investigate differences in the proportion of children receiving timely MMR vaccination in each cohort, by these covariates.

To describe geographical clustering of MMR vaccination timeliness we will plot in choropleth maps the proportion of children receiving timely MMR vaccination in each cohort, by LSOA.

We will conduct binary logistic regression to estimate the odds (odds ratio [OR] and 95% CI) of timely MMR vaccination after adjustment for covariates and, to assess whether COVID-19 has widened inequalities in timely vaccination, explore an interaction between cohort and IMD quintile.

For peer review only

Ethics

This study was approved by the Discovery board for service evaluation (measuring what standard of care this service achieved) and analysed routinely acquired de-identified data: hence no research ethics committee approval was required by the Health Research Authority. Access to general practice data is enabled by data sharing agreements between the Discovery Data Service and general practice data controllers. The Discovery Programme Board has approved data access by the REAL Child Health programme.

Strengths and limitations

Strengths of this study include:

- Use of person-level data to comprehensively document the impact of COVID-19 on timely MMR vaccine uptake and coverage
- EHR from seven geographically contiguous CCGs, serving a whole population with a large sample size, including children who have left/deregistered from a practice in NEL, or died
- Access to historic data "pre-pandemic" cohort to use as control group
- Implementation of robust statistical methods

Limitations of this study include:

- Use of calendar week of birth to generate a proxy date of birth
- Primary purpose of EHRs is not for research – so it is possible there will be some data quality issues
  - Large proportions of children with missing ethnic background recorded in their EHR

Funding

This research was funded by a grant from Barts Charity ref: MGU0419. This work was supported by Health Data Research UK (award reference: GPPB1C2), which is funded by the UK Medical Research Council, Engineering and Physical Sciences Research Council, Economic and Social Research Council, Department of Health and Social Care (England), Chief Scientist Office of the Scottish Government Health and Social Care Directorates, Health and Social Care Research and Development Division (Welsh Government), Public Health Agency (Northern Ireland), British Heart Foundation and Wellcome.

References

1. Saxena S, Skirrow H, Bedford H. Routine vaccination during covid-19 pandemic response. Bmj. 2020;369:m2392.
2. Public Health England. Impact of physical distancing measures due to COVID-19 pandemic in England on childhood vaccination counts up to week 41, and vaccine coverage up to August 2020. 2020. Contract No.: 20.
3. Public Health England. Impact of COVID-19 on childhood vaccination counts to week 47, and vaccine coverage to October 2020 in England: interim analyses. Public Health England; 2020 8 December 2020. Contract No.: 23.
4. Bell SL, Clarke R, Paterson P, Mounier-Jack S. Parents' and guardians' views and experiences of accessing routine childhood vaccinations during the coronavirus (COVID-19) pandemic: A mixed-methods study in England 2020.
5. O'Leary ST, Trefren L, Roth H, Moss A, Severson R, Kempe A. Number of Childhood and Adolescent Vaccinations Administered Before and After the COVID-19 Outbreak in Colorado. JAMA pediatrics. 2020.
6. Spencer N, Nathawad R, Arpin E, Johnson S. Pandemics, epidemics and inequities in routine childhood vaccination coverage: a rapid review. BMJ Paediatrics Open. 2020;4(1).
7. McDonald HI, Tessier E, White JM, Woodruff M, Knowles C, Bates C, et al. Early impact of the coronavirus disease (COVID-19) pandemic and physical distancing measures on routine childhood vaccinations in England, January to April 2020. Euro Surveill. 2020;25(19).

For peer review only

8. Skirrow H, Flynn C, Heller A, Heffernan C, Mounier-Jack S, Chantler T. Delivering routine immunisations in London during the COVID-19 pandemic: lessons for future vaccine delivery. A mixed-methods study. BJGP Open. 2021;5(4):BJGPO.2021.0021.
9. Childhood Vaccination Coverage Statistics [Internet]. National Statistics. 2021. Available from: https://app.powerbi.com/view?r=eyJrIjoiZTI3NWZhNzItMTIyZS00OWM2LTg0MzMtOGY5YTJjMGY0MjI1IiwidCI6IjUwZjYwNzFmLWJiZmUtNDAxYS04ODAzLTY3Mzc0OGU2MjllMiIsImMiOjh9.
10. Keenan A, Ghebrehewet S, Vivancos R, Seddon D, MacPherson P, Hungerford D. Measles outbreaks in the UK, is it when and where, rather than if? A database cohort study of childhood population susceptibility in Liverpool, UK. BMJ Open. 2017;7(3):e014106.
11. National Audit Office. Investigation into pre-school vaccinations. 2019. Contract No.: 007836.
12. Tiley KS, White JM, Andrews N, Ramsay M, Edelstein M. Inequalities in childhood vaccination timing and completion in London. Vaccine. 2018;36(45):6726-35.
13. Department for Communities and Local Government. The English Indices of Deprivation 2015 - Frequently Asked Questions (FAQs). 2016.
14. Public Health England. Measles: the green book, chapter 21. In: Public Health England, editor. The Green Book2019.

For peer review only

STROBE 2007 (v4) Statement—Checklist of items that should be included in reports of *cross-sectional studies*

Section/Topic	Item #	Recommendation	Reported on page #/in section
**Title and abstract**	1	(*a*) Indicate the study's design with a commonly used term in the title or the abstract	Title
		(*b*) Provide in the abstract an informative and balanced summary of what was done and what was found	Abstract
**Introduction**			
Background/rationale	2	Explain the scientific background and rationale for the investigation being reported	Introduction
Objectives	3	State specific objectives, including any prespecified hypotheses	Introduction
**Methods**			
Study design	4	Present key elements of study design early in the paper	Methods – study design and setting
Setting	5	Describe the setting, locations, and relevant dates, including periods of recruitment, exposure, follow-up, and data collection	Methods – study design and setting
Participants	6	(*a*) Give the eligibility criteria, and the sources and methods of selection of participants	Methods – study population
Variables	7	Clearly define all outcomes, exposures, predictors, potential confounders, and effect modifiers. Give diagnostic criteria, if applicable	Methods – outcome of interest
Data sources/ measurement	8*	For each variable of interest, give sources of data and details of methods of assessment (measurement). Describe comparability of assessment methods if there is more than one group	Methods – covariates of interest
Bias	9	Describe any efforts to address potential sources of bias	Methods – statistical analyses
Study size	10	Explain how the study size was arrived at	Methods – data processing and Supplementary file 3 Figure S2
Quantitative variables	11	Explain how quantitative variables were handled in the analyses. If applicable, describe which groupings were chosen and why	Methods – covariates of interest
Statistical methods	12	(*a*) Describe all statistical methods, including those used to control for confounding	Methods – statistical analyses
		(*b*) Describe any methods used to examine subgroups and interactions	Methods – statistical analyses
		(*c*) Explain how missing data were addressed	Methods – covariates of interest and statistical analyses

For peer review only

		(*d*) If applicable, describe analytical methods taking account of sampling strategy	n/a
		(*e*) Describe any sensitivity analyses	n/a
**Results**			
Participants	13*	(a) Report numbers of individuals at each stage of study—eg numbers potentially eligible, examined for eligibility, confirmed eligible, included in the study, completing follow-up, and analysed	Results and Supplementary file 3 Figure S2
		(b) Give reasons for non-participation at each stage	Supplementary file 3 Figure S2
		(c) Consider use of a flow diagram	Supplementary file 3 Figure S2
Descriptive data	14*	(a) Give characteristics of study participants (eg demographic, clinical, social) and information on exposures and potential confounders	Table 1
		(b) Indicate number of participants with missing data for each variable of interest	Table 1
Outcome data	15*	Report numbers of outcome events or summary measures	Table 2
Main results	16	(*a*) Give unadjusted estimates and, if applicable, confounder-adjusted estimates and their precision (eg, 95% confidence interval). Make clear which confounders were adjusted for and why they were included	Supplementary file 3 Table S5 and Figure 4
		(*b*) Report category boundaries when continuous variables were categorized	n/a
		(*c*) If relevant, consider translating estimates of relative risk into absolute risk for a meaningful time period	n/a
Other analyses	17	Report other analyses done—eg analyses of subgroups and interactions, and sensitivity analyses	Results
**Discussion**			
Key results	18	Summarise key results with reference to study objectives	Discussion – summary of key findings
Limitations	19	Discuss limitations of the study, taking into account sources of potential bias or imprecision. Discuss both direction and magnitude of any potential bias	Discussion – strengths and limitations
Interpretation	20	Give a cautious overall interpretation of results considering objectives, limitations, multiplicity of analyses, results from similar studies, and other relevant evidence	Discussion – implications for research, policy and practice
Generalisability	21	Discuss the generalisability (external validity) of the study results	Discussion – comparison with existing literature
**Other information**			
Funding	22	Give the source of funding and the role of the funders for the present study and, if applicable, for the original study on which the present article is based	Funding

*Give information separately for cases and controls in case-control studies and, if applicable, for exposed and unexposed groups in cohort and cross-sectional studies.

For peer review only

Note: An Explanation and Elaboration article discusses each checklist item and gives methodological background and published examples of transparent reporting. The STROBE checklist is best used in conjunction with this article (freely available on the Web sites of PLoS Medicine at http://www.plosmedicine.org/, Annals of Internal Medicine at http://www.annals.org/, and Epidemiology at http://www.epidem.com/). Information on the STROBE Initiative is available at www.strobe-statement.org.

For peer review only

Supplementary tables and figures

Figure S1 – Identifying registrations at the time of first birthday

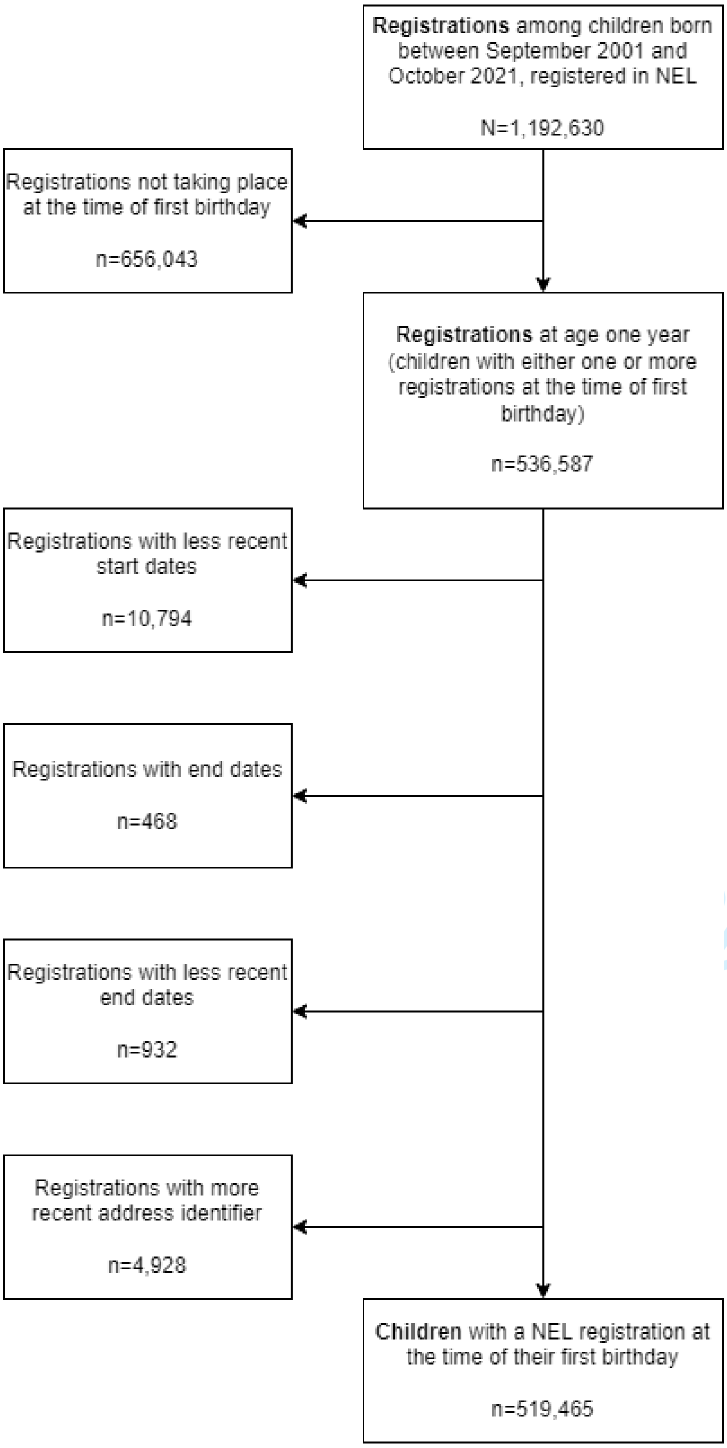

We excluded 656,043 (55.0% of 1,192,630) registrations, which were not current at 12 months of age. For children with multiple concurrent registrations at the time of their first birthday, we applied the following sequence of hierarchical rules to determine which registration should be retained:

Retain

1. *registration with the most recent start date*
2. *registration with a NULL end date (considered more recent/ongoing)*
3. *registration with the most recent end date*
4. *registration with the lowest address identification number (children may have multiple registrations starting and ending on the same date as a result of practice mergers and closures. The lower address identifier is assumed to relate to the original registration)*

Figure S2 – Processing the MMR data file

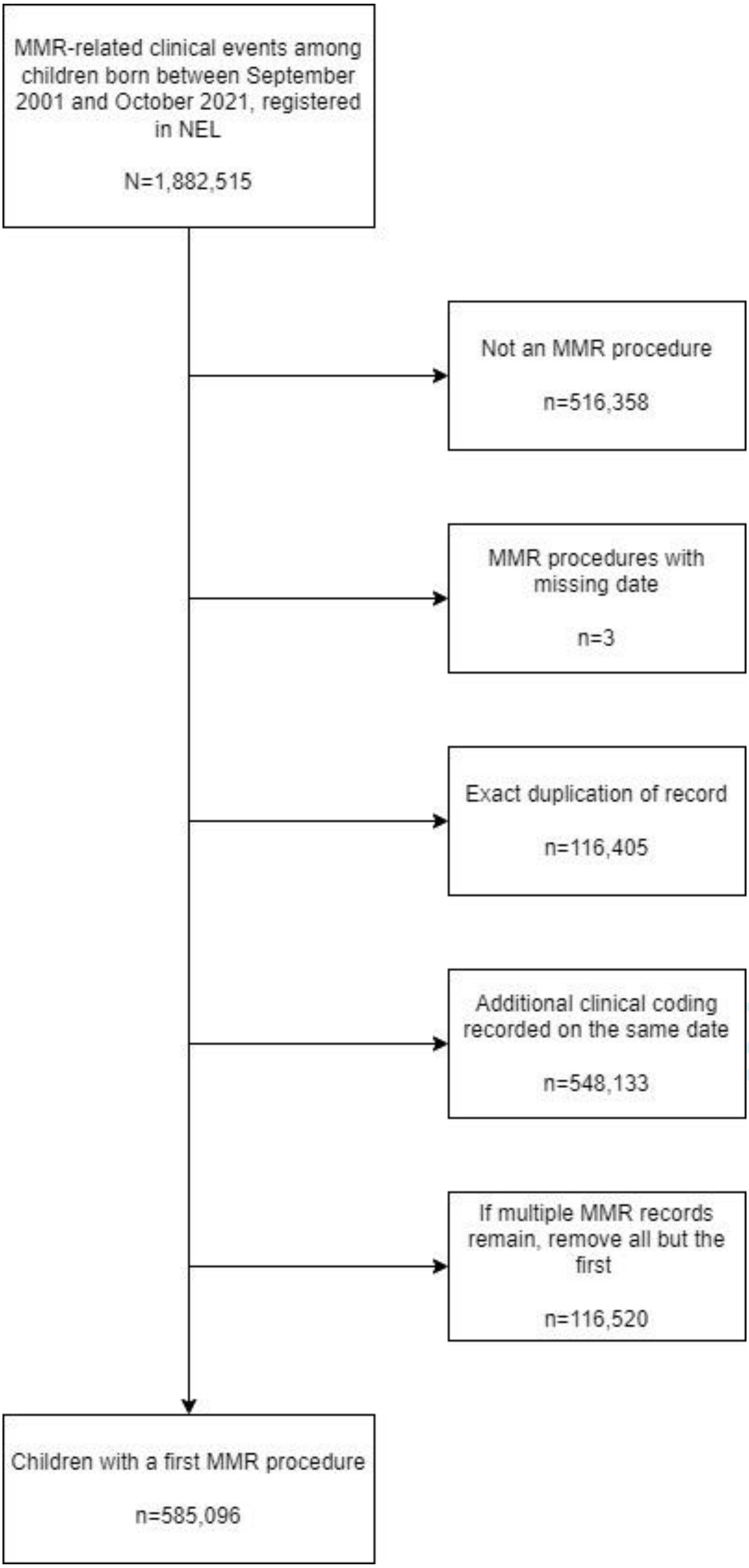

*Data relating to MMR vaccinations were extracted separately. The data file was in long format where each row was a unique clinical code, therefore children often have multiple rows indicating various MMR administrative processes and procedures throughout their life, including vaccination invitations, declines, procedures, contraindications, appointment non-attendance, as well as clinical codes relating to the second MMR vaccination. For the purposes of this study, it was necessary to identify the **first MMR procedure** for each child. Of 1,882,515 events, 516,358 recorded events were removed as they were not considered to be a first MMR procedure (see table S1). Records without a date (n=3), as well as duplicate records were removed (n=664,538). Finally, all but the earliest event if a child had more than one MMR vaccination recorded were removed (n=116,520). MMR vaccinations given before the child's first birthday were excluded if the child received another dose after turning 12 months of age, as per guidance from The Green Book.[1] Where available, subsequent MMR records were treated as the child's first vaccination, otherwise early MMR vaccinations were retained in the dataset and later considered as "not timely".*

[1] Public Health England. Measles: the green book, chapter 21. In: Public Health England, ed. The Green Book; 2019.

Table S1 - SNOMED clinical codes for first MMR procedures

Events recorded in the primary care electronic heath record using another clinical coding system (e.g. Read v2 or EMIS local codes) have been mapped to relevant SNOMED codes within the Discovery Data Service. This ensures that searching the database using SNOMED codes captured all events regardless of the clinical coding system used.

SNOMED concept ID	Other code	Clinical coding scheme	Code description
38598009	38598009	SNOMED	Measles-mumps-rubella vaccination (procedure)
	65M1.	Read v2	Measles/mumps/rubella vaccn.
	^ESCT1405772	EMIS local	Administration of measles and mumps and rubella vaccine
47435007	47435007	SNOMED	Measles vaccination (procedure)
	65A..	Read v2	Measles vaccination
	65A1.	Read v2	Measles vaccination
	ZV042	Read v2	[V]Measles vaccination
	^ESCT1405845	EMIS local	Administration of measles vaccine
50583002	50583002	SNOMED	Mumps vaccination (procedure)
	65F5.	Read v2	Mumps vaccination
	ZV046	Read v2	[V]Mumps vaccination
	^ESCT1405876	EMIS local	Administration of mumps vaccine
82314000	65B..	Read v2	Rubella vaccination
	ZV043	Read v2	[V]Rubella vaccination
	^ESCT1406118	EMIS local	Administration of rubella vaccine
170364006	65A2.	Read v2	Measles vaccin.+immunoglobulin
432636005	^ESCT1408534	EMIS local	Administration of measles and mumps and rubella and varicella virus vaccine
871909005	^ESCT1397548	EMIS local	Administration of first dose of measles and mumps and rubella and varicella virus vaccine
150971000119104	ZV064	Read v2	[V]Measles-mumps-rubella (MMR) vaccination
308081000000105	65M10	Read v2	First MMR (measles mumps and rubella) vaccination
	Xaeec	Read v3	First MMR (measles mumps and rubella) vaccination
	^ESCTME809974	EMIS local	Measles mumps and rubella vaccination - first dose
505001000000109	9ki1.	Read v2	MMR catch-up vaccination - enhanced services administration
	XaQPr	Read v3	Measles mumps rubella catch-up vaccination
571591000119106	^ESCT1409651	EMIS local	Administration of live attenuated measles mumps and rubella vaccine
1037251000000100	65M11	Read v2	First MMR vaccination given by other healthcare provider
	Xaeeq	Read v3	First MMR vaccination given by other healthcare provider

We included clinical codes relating to administration of mono-components of the first MMR vaccination. After removal of duplicate data entries and merging to the study cohort, 533 children had a clinical code for measles vaccination, and two for mumps vaccination, as opposed to a combined MMR vaccination.

For peer review only

Table S2 – Proportion of children within each cohort who had a MMR procedure recorded in their electronic primary care record

The two datafiles (registrations and MMR procedures) were merged. 531,469 children with an MMR procedure were not matched to the study denominator because they were not eligible for a timely MMR vaccination in the 19 months before or after 23rd March 2020.

	MMR procedure	No MMR procedure	Total
Pre-pandemic (%)	28,173 (84.8)	5,053 (15.2)	33,226 (100)
Pandemic (%)	25,454 (78.5)	6,992 (21.5)	32,446 (100)

Table S3 – SNOMED clinical codes for measles and mumps diagnoses

Code term	SNOMED Concept ID
Measles (disorder)	14189004
Mumps (disorder)	36989005

Table S4 – Ethnic background distribution by Clinical Commissioning Group

	White (%)	Mixed and Other (%)	South Asian (%)	Black (%)	Missing (%)	Total (%)
**Barking & Dagenham**	2,136 (27.6)	567 (7.3)	1,164 (15.1)	772 (10.0)	3,096 (40.0)	7,735 (100)
**City & Hackney**	2,713 (28.9)	1,942 (20.7)	162 (1.7)	576 (6.1)	4,009 (42.6)	9,402 (100)
**Havering**	3,171 (43.2)	500 (6.8)	484 (6.6)	353 (4.8)	2,833 (38.6)	7,341 (100)
**Newham**	2,698 (21.3)	1,656 (13.1)	3,572 (28.2)	1,083 (8.6)	3,659 (28.9)	12,668 (100)
**Redbridge**	2,595 (26.6)	1,032 (10.6)	2,821 (28.9)	404 (4.1)	2,912 (29.8)	9,764 (100)
**Tower Hamlets**	1,223 (13.3)	585 (6.4)	2,419 (26.3)	261 (2.8)	4,715 (51.2)	9,203 (100)
**Waltham Forest**	3,981 (41.7)	1,297 (13.6)	1,061 (11.1)	597 (6.3)	2,623 (27.4)	9,559 (100)
**Total**	18,517 (28.2)	7,579 (11.5)	11,683 (17.8)	4,046 (6.2)	23,847 (36.3)	65,672 (100)

For peer review only

Table S5 – Index of Multiple Deprivation quintile associated with LSOAs with less than 60% of children receiving timely MMR vaccination in the pre-pandemic and pandemic cohorts

	LSOAs with <60% timely vaccination in pre-pandemic cohort (*n*=90)		LSOAs with <60% timely vaccination in pandemic cohort (*n*=153)		All LSOAs (*n*=1,203)	
	**n**	**%**	**n**	**%**	**n**	**%**
**Most deprived**	30	33.3	63	41.2	401	33.3
**2**	45	50.0	61	39.9	445	37.0
**3**	9	10.0	19	12.4	187	15.5
**4**	2	2.2	6	3.9	107	8.9
**Least deprived**	2	2.2	4	2.6	58	4.8
**Missing**	2	2.2	0	0.0	5	0.4
**Total**	90	100	153	100	1203	100

Table S6 – Unadjusted and adjusted odds of timely MMR vaccination

	Unadjusted		Adjusted[1] (*n*=65572[2])	
	OR[3]	95% CI[4]	OR[3]	95% CI[4]
**Cohort**				
Control (ref.)	1		1	
COVID-19	0.80	0.77,0.83	0.79	0.76,0.82
**Sex**				
Male (ref.)	1		1	
Female	1.07	1.03,1.11	1.07	1.03,1.11
**CCG[5]**				
Barking & Dagenham	0.84	0.78,0.89	0.88	0.82,0.94
City & Hackney	0.60	0.56,0.63	0.67	0.63,0.71
Havering	1.78	1.64,1.92	1.53	1.40,1.66
Newham (ref.)	1		1	
Redbridge	0.82	0.77,0.87	0.69	0.64,0.73
Tower Hamlets	1.52	1.42,1.63	1.52	1.42,1.64
Waltham Forest	1.21	1.13,1.29	1.21	1.14,1.30
**Ethnic background**				
White (ref.)	1		1	
Mixed and Other	0.68	0.64,0.73	0.77	0.72,0.82
South Asian	1.33	1.25,1.41	1.39	1.30,1.48
Black	0.64	0.59,0.69	0.70	0.65,0.76
Missing	0.75	0.72,0.79	0.77	0.74,0.81
**IMD[6] quintile**				
Most deprived (ref.)	1		1	
2	1.03	0.99,1.07	1.03	0.99,1.08
3	1.26	1.18,1.33	1.24	1.16,1.32
4	1.87	1.71,2.06	1.78	1.61,1.98
Least deprived	2.42	2.07,2.82	2.09	1.78,2.46

[1] Model mutual adjusting for cohort, sex, clinical commissioning group, ethnic background and Index of Multiple Deprivation quintile. [2] 98 children with missing Index of Multiple Deprivation quintile and two children with "Other" sex were excluded from the adjusted model. [3] Odds ratio. [4] 95% confidence interval. [5] Clinical Commissioning Group. [6] Index of Multiple Deprivation.

For peer review only

Table S7 – Measles and mumps cases recorded in primary care in the pre-pandemic and pandemic periods by Clinical Commissioning Group

	Pre-pandemic	Pandemic
**Measles**	325	20
**Mumps**	140	34

For peer review only
